# The mTOR inhibitor rapamycin suppresses trigeminal neuropathic pain and p-MKK4/p-p38 mitogen-activated protein kinase-mediated microglial activation in the trigeminal nucleus caudalis of mice with infraorbital nerve injury

**DOI:** 10.3389/fnmol.2023.1172366

**Published:** 2023-04-14

**Authors:** Ji-Hee Yeo, Dae-Hyun Roh

**Affiliations:** Department of Oral Physiology, College of Dentistry, Kyung Hee University, Seoul, Republic of Korea

**Keywords:** rapamycin (CID: 5284616), mTOR – mammalian target of rapamycin, trigeminal neuropathic pain, p38 MAPK, microglia

## Abstract

Neuropathic pain caused by trigeminal nerve injury is a typical refractory orofacial chronic pain accompanied by the development of hyperalgesia and allodynia. We previously demonstrated that the mammalian target of rapamycin (mTOR) inhibitor rapamycin suppressed orofacial formalin injection-induced nociception; however, the underlying mechanism is unclear, and it is unknown whether it can reduce trigeminal neuropathic pain. In mice, left infraorbital nerve and partial nerve ligation (ION-pNL) was performed using a silk suture (8–0). Fourteen days after surgery, neuropathic pain behavior was examined on a whisker pad and rapamycin (0.1, 0.3, and 1.0 mg/kg) was administered intraperitoneally. Mechanical and cold sensitivities in the orofacial region were quantified using von Frey filaments and acetone solution, respectively. Changes in mTOR and related proteins, such as p-MKK3/6, p-MKK4, p-JNK, p-ERK, p-p38 MAPK, GFAP, and Iba-1, in the trigeminal nucleus caudalis (TNC) or the trigeminal ganglia (TG) tissues were examined *via* western blot analysis or immunohistochemistry. Mice demonstrated significant mechanical and cold allodynia 2 weeks following ION-pNL injury, both of which were significantly reduced 1 h after the administration of high-dose rapamycin (1.0 mg/kg). In the TG tissue, ION-pNL surgery or rapamycin treatment did not change p-mTOR and p-4EBP1, but rapamycin reduced the increase of p-S6 and S6 induced by ION-pNL. In the TNC tissue, neither ION-pNL surgery nor rapamycin treatment altered p-mTOR, p-S6, and p-4EBP1 expressions, whereas rapamycin significantly decreased the ION-pNL-induced increase in Iba-1 expression. In addition, rapamycin suppressed the increase in p-p38 MAPK and p-MKK4 expressions but not p-MKK3/6 expression. Moreover, p-p38 MAPK-positive cells were colocalized with increased Iba-1 in the TNC. Our findings indicate that rapamycin treatment reduces both mechanical and cold orofacial allodynia in mice with trigeminal neuropathic pain, which is closely associated with the modulation of p-MKK4/p-p38 MAPK-mediated microglial activation in the TNC.

## 1. Introduction

Orofacial pain disorders are associated with a wide range of conditions, including trigeminal neuralgia, temporomandibular joint disorders, periodontal pain, and atypical facial pain. Neuropathic pain in the orofacial area markedly reduces the quality of life by disrupting food intake, face washing, and tooth brushing. Thus, orofacial pain may be particularly distressing to a patient because of the psychological and emotional context of this body area. In addition, neuropathic pain related to trigeminal nerve injury is commonly observed in clinical trials and persists for an extended period of time, even after healing of overt tissue damage (Costigan et al., 2009). Therefore, to develop appropriate therapeutic strategies, it is essential to identify the mechanism responsible for trigeminal neuropathic pain as well as understand the underlying mechanisms of existing therapeutic drugs.

Mammalian target of rapamycin (mTOR) is a highly conserved serine/threonine protein kinase that is widespread throughout the nervous system. It is closely associated with pain sensation and regulates changes in synaptic plasticity (Jaworski and Sheng, 2006). Increased mTOR signaling has been reported to induce neuronal hyperexcitability (Asante et al., 2009; Lasarge and Danzer, 2014), which may be the basis for neuropathic pain (Carlton et al., 2009). Moreover, protein translation triggered by mTORC1 signaling is necessary for the initiation and maintenance of chronic pain (Lutz et al., 2015). Thus, several studies have indicated that the mTOR signaling pathway may play an important role in the development and progression of chronic pain in various neuropathic pain models (Obara et al., 2011; Li et al., 2015; Tateda et al., 2017; Ma et al., 2020). Other studies have shown that rapamycin, a representative mTOR inhibitor, performs both neuroprotective and neuroregenerative functions in trauma and various diseases of the central nervous system (CNS) (Cawley et al., 1998; Ravikumar et al., 2004; Ma et al., 2010; Malagelada et al., 2010; Kanno et al., 2012). Previously, we demonstrated that rapamycin reduced nociceptive responses in the first and second phases of the mouse orofacial formalin test. In addition, this antinociceptive effect of rapamycin was associated with the inhibition of p38 mitogen-activated protein kinase (MAPK) in the trigeminal nucleus caudalis (TNC) (Yeo et al., 2021). However, whether rapamycin reduces allodynic behavior in a trigeminal neuropathic pain mouse model and its underlying mechanisms remain to be elucidated.

Both astrocytes and microglia play important roles in the modulation of synaptic function and neuronal excitability. They may contribute to the formation and development of neuron–glia crosstalk and central sensitization that are responsible for the chronic state of neuropathic pain (Watkins et al., 2001). A variety of MAPKs, including extracellular signal-regulated kinase (ERK) and Jun N-terminal kinase (JNK), are involved in these processes, with p38 MAPK providing a link between extracellular stimuli and intracellular responses (Pearson et al., 2001). In addition, activated microglia produce inflammatory mediators, such as interleukin (IL)-1β, IL-6, and tumor necrosis factor (TNF)-α, which are also produced in the spinal cord following peripheral nerve injury. Most of these inflammatory mediators are implicated in pain facilitation or hyperalgesia in animals and humans (Sweitzer et al., 2001; Watkins et al., 2001; Winkelstein et al., 2001; Milligan et al., 2003; Tsuda et al., 2005). In particular, p38 MAPK is known to regulate the synthesis of various inflammatory mediators through transcriptional regulation (Ji and Woolf, 2001; Kumar et al., 2003). Recently, mTORC1 activation was shown to increase the synthesis of MKK6 protein and attenuate the activation of the p38 MAPK-p53 signaling pathway, leading to a decrease in the number and activity of intestinal stem cells (He et al., 2020). This suggests that p38 MAPK is a downstream modulator of mTORC1 signaling.

In the present study, we sought to determine (1) whether rapamycin treatment produces a dose-dependent antiallodynic effect in the orofacial region in an infraorbital nerve-injured mouse model; (2) whether changes in mTOR signaling-related proteins in the TNC and trigeminal ganglion (TG) are involved in this antiallodynic effect; (3) whether the activation of glial cells (astrocytes or microglia) and phosphorylation of ERK, JNK, and p38 MAPK in the TNC are affected by rapamycin treatment; and (4) whether the upstream activators of MAPKs (e.g., MKK3/6 or MKK4) mediate the action of rapamycin.

## 2. Materials and methods

### 2.1. Animals

Male C57BL/6 mice (25–30 g; DBL, Seoul, Korea) were housed in colony cages with free access to food and water and maintained in temperature- and light-controlled rooms (23°C ± 2°C, 12/12-h light/dark cycle with lights on at 07,00) for at least 1 week prior to the experiments. The protocols for the animal experiments were reviewed and approved by the Kyung Hee University Institutional Animal Care and Use Committee (KHUASP(SE)-16–014) and conformed to the National Institutes of Health guidelines (NIH publication No. 86–23, revised 1985).

### 2.2. Surgical procedures

**Figure 1:**
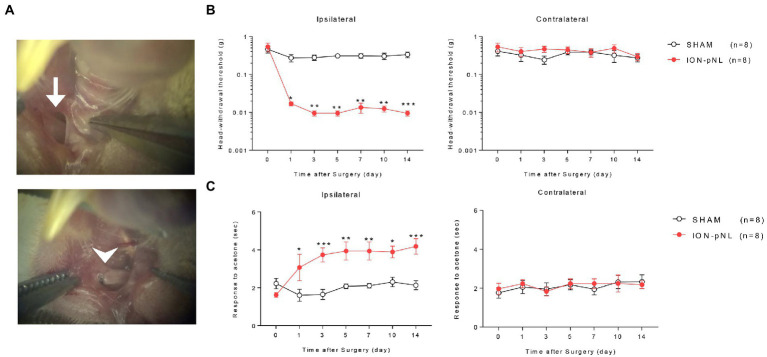
Development of a mouse model for chronic mechanical and cold allodynia after infraorbital nerve injury. Images in the left panel show the infraorbital nerve and partial ligation (ION-pNL) surgical approach **(A)**. The left ION was exposed (arrow) and tightly ligated with 8–0 silk at approximately 2/3 the nerve diameter (arrowhead). Infraorbital nerve injury group (ION-pNL) showing a significant decrease in 50% head withdrawal threshold from day 1 to 14 only on the ipsilateral side following infraorbital nerve injury **(B)**. *p < 0.05, **p < 0.01, ***p < 0.001 compared with the sham surgery group (SHAM). In addition, the rubbing response time to acetone application was significantly increased in ION-pNL mice from day 1 to 14 following infraorbital nerve injury **(C)**. *p < 0.05, **p < 0.01, ***p < 0.001 as compared with SHAM, *n* = 8 per group.

As described previously (Zhang et al., 2017), the mice were anesthetized with 3% isoflurane in a mixture of N_2_O/O_2_ gas. Partial ligation of the left infraorbital nerve was performed using an intraoral approach. The oral cavity was exposed and a longitudinal incision (4 mm) on the buccal mucosa at the level of the maxillary first molar was introduced to expose the left infraorbital nerve (ION). The ION was carefully isolated using fine forceps without damaging nearby facial nerve branches. Approximately 2/3 of the nerve diameter was tightly ligated with an 8–0 silk suture (Figure 1A). After checking for hemostasis, the incision was closed with a 5–0 silk suture. The sham procedure consisted of exposing the ION, with care taken to avoid stretching the nerve or damaging the epineurium.

### 2.3. Rapamycin treatment

Rapamycin (LC laboratories, Woburn, MA) was dissolved in dimethyl sulfoxide (DMSO) to prepare a 100 mg/mL stock solution and dissolved in physiological saline at concentrations of 0.1, 0.3, and 1.0 mg/kg immediately before injection. The mice were weighed and rapamycin was injected intraperitoneally. The doses of rapamycin were selected based on a previous study (Yeo et al., 2021). The final vehicle (VEH) was adjusted to 0.2% DMSO in saline.

### 2.4. Pain behavioral tests

All behavioral experiments were conducted in a quiet room. Mice were acclimatized to the handling and testing equipments 30 min before the experiments. To evaluate mechanical allodynia, the body of the mouse was gently wrapped and restrained with a soft cloth, and von Frey filaments (North Coast Medical, Morgan Hill, CA, USA) were used to measure the mechanical withdrawal threshold in both whisker pads of the face. An ascending series of von Frey filaments that delivered approximately logarithmic incremental bending forces (0.008, 0.02, 0.04, 0.07, 0.16, 0.4, and 0.6 g) was used to evaluate mechanical allodynia. Starting with the monofilament with the lowest force of 0.008 g, each monofilament was applied six times to the left and right whisker pads before applying the next higher force monofilament. The monofilament that produced a response of face wiping or rubbing with the forelimb in three out of the six applications was defined as the 50% mechanical withdrawal threshold.

For the cold behavioral test, mice were placed into an acrylic cylinder (25 × 25 cm) with each other and 10 μL of 100% acetone was topically applied to the right whisker pad skin using a customized 27-gauge needle attached to a PE 20 tube. Immediately after acetone administration, the animals were returned to the acrylic cylinder and nociceptive behavior was recorded during a 1-min period. Nociceptive behavior was evaluated as asymmetric orofacial wiping or rubbing on the whisker pad, executed with the ipsilateral forelimb (Shimada and LaMotte, 2008). Scratching the face with the hindlimb was considered an itch response and was excluded from the data. For each mouse, the time spent wiping or rubbing was monitored using a stopwatch.

### 2.5. Western blot assay

Mice were deeply anesthetized with 3% isoflurane in a mixture of N_2_O/O_2_ gas and euthanized on day 14 post ION-pNL surgery or 1 h following rapamycin injection. Western blot analysis was performed as described in our previous report (Yoon et al., 2017). The TNCs and TGs were dissected, quickly frozen in liquid nitrogen, and stored at −80°C until processed. TNC and TG tissues were homogenized *via* sonication in RIPA lysis buffer (50 mM Tris–HCl, pH 7.4, 1% NP-40, 0.25% sodium deoxycholate, 150 mM NaCl, 1 mM EDTA) containing protease and phosphatase inhibitor cocktails (Sigma, St. Louis, MO). The sonicated TNC and TG tissues were incubated at 4°C for 1 h on a rotator and centrifuged for 15 min at 15,000 rpm at 4°C. The protein concentration of the resulting supernatant was measured using the Lowry protein assay (Bio-Rad, Hercules, CA). Protein samples (25 μg) were separated on an SDS-PAGE gel and transferred to a polyvinylidene fluoride (PVDF) membrane using a Transblot SD apparatus (Bio-Rad). The membranes were blocked with 5% skim milk at room temperature (RT) for 30 min and incubated at 4°C overnight with primary antibodies, followed by incubation with horseradish peroxidase-conjugated secondary antibodies at RT for 1 h. Primary antibody concentrations were as follows: rabbit anti-phospho-mTOR (1:1000, Cell Signaling Technology, Beverly, MA), rabbit anti-mTOR (1:1000, Cell Signaling), rabbit anti-phospho-S6 (1:2000, Cell Signaling), rabbit anti-S6 (1:2000, Cell Signaling), rabbit anti-phospho-4EBP1 (1:2000, Cell Signaling), rabbit anti-4EBP1 (1:2000, Cell Signaling), rabbit anti-phospho-JNK (p-JNK, 1:1000, Cell Signaling), rabbit anti-JNK (1:1000, Cell Signaling), rabbit anti-phospho-p44/42 MAPK (p-ERK1/2, 1:1000, Cell Signaling), rabbit anti-ERK1/2 (1:1000, Cell Signaling), rabbit anti-phospho-p38 MAPK (p-p38, 1:1000, Cell Signaling), rabbit anti-p38 (1:2000, Cell Signaling), rabbit anti-phospho-MKK3/6 (1:1000, Cell signaling), rabbit anti-MKK3 (1:1000, Cell Signaling), rabbit anti-MKK6 (1:1000, Cell Signaling), rabbit anti-phospho-MKK4 (1:1000, Cell Signaling), and rabbit anti-MKK4 (1:1000, Cell Signaling). β-actin was used as the loading control (1,10,000, Sigma). The bands were detected and visualized using an enhanced chemiluminescence system (Amersham Pharmacia Biotech, Little Chalfont, UK), and band intensity was measured and analyzed using ImageJ 1.50i software (National Institutes of Health, Bethesda, MD).

### 2.6. Immunohistochemistry

Immunohistochemistry was performed on TNC and TG tissues as previously described (Roh and Yoon, 2014; Yoon et al., 2015). On day 14 post ION-pNL surgery or 1 h following rapamycin injection, the mice were deeply anesthetized with 5% isoflurane and perfused transcardially through the ascending aorta with 0.1 M phosphate-buffered saline (PBS, 50 mL pH 7.4), followed by 10% neutral-buffered formalin (100 mL, Sigma). After perfusion, the brainstem was removed immediately, stored overnight at 4°C in the same fixative, and placed into a cryoprotectant solution (30% sucrose in PBS) for at least two nights at 4°C before sectioning. Serial transverse sections were cut from the TNC and TG tissues (30-μm- and 10-μm-thick sections, respectively) using a cryostat (Leica Microsystems, Wetzlar, Germany) and collected in PBS. After preblocking with 5% normal donkey serum plus 0.3% Triton X-100 in PBS at RT for 1 h, the free-floating TNC sections were incubated overnight at 4°C with primary antibodies against phospho-p38 (1:1000, Cell Signaling), NeuN (1:1000, Milipore, Burlington, Massachusetts), GFAP (1:1000, Cell Signaling), Iba-1 (1:500, Wako, Osaka, Japan), and p-S6 (1:500, Cell Signaling) in a diluent (1% normal donkey serum plus 0.3% Triton X-100 in PBS). Following several washes with PBS, the tissue sections were incubated with Cy3-conjugated (1:500, Jackson ImmunoResearch, West Grove, PA) and Alexa Fluor 488-conjugated (1:500, Jackson ImmunoResearch) secondary antibodies for 4 h at RT. After several washes with PBS, the tissue sections were mounted onto slides under a cover slip.

### 2.7. Image analysis

The TNC and TG tissues were scanned using an ECLIPSE 80i (Nikon Corp., Kanagawa, Japan) fluorescent microscope, and individual sections were digitized with 4,096 gray levels using a cooled CCD camera (Cool Snap ES model, Nihon Roper, Tokyo, Japan) connected to a computer-assisted image analysis system (MetaMorph; Universal Imaging, Westchester, PA). As previously described (Yeo et al., 2021), for quantitative analysis in the TNC region, six nonadjacent tissue sections per mouse were randomly selected and analyzed with a computer-assisted image analysis system (MetaMorph version 7.7.2.0). To analyze the p-p38 images, cells that were at least 80% brighter than the average level of each image were counted. For GFAP and Iba-1 immunoreactivity (Sorkin et al., 2009), the positive pixel areas that had a brightness level of 80% within the range of intensity levels were measured. The average percentage threshold area of immunoreactivity per section from each animal was obtained, and these values were averaged across each group and presented as group data. All analytical procedures described above were blindly performed without the knowledge of the experimental conditions.

### 2.8. Statistical analysis

All values are expressed as mean ± S.E.M. Data analysis and statistical comparisons were calculated using GraphPad Prism version 6.0 (GraphPad Software, San Diego, CA). For multiple comparisons of the pain behavior tests, two-way repeated measures analysis of variance (ANOVA) followed by Bonferroni’s test were used. Data from western blot and immunohistochemical assays were analyzed using one-way ANOVA followed by Bonferroni’s *post hoc* test. A *p* value of <0.05 was considered statistically significant.

## 3. Results

### 3.1. Infraorbital nerve partial ligation causes orofacial neuropathic pain in mice

Head withdrawal response was examined using von Frey filaments to evaluate the mechanical nociceptive threshold in ION-pNL mice. The head withdrawal threshold for ipsilateral side stimuli was significantly decreased in ION-pNL mice compared with that in sham surgery animals (SHAM) from 1 to 14 days following surgery (*p* = 0.0128, *p* = 0.0078, *p* = 0.0022, *p* = 0.0026, *p* = 0.0031, *p* = 0.0007, respectively), whereas the head withdrawal threshold for contralateral side stimuli was unchanged (Figure 1B). The wiping or rubbing response to acetone stimuli on the ipsilateral whisker pad significantly increased in the ION-pNL group compared with the SHAM group from 1 to 14 days following surgery (*p* = 0.0303, *p* = 0.0005, *p* = 0.0024, *p* = 0.0028, *p* = 0.0146, *p* = 0.0006, respectively), whereas nociceptive responses to cold stimuli on the contralateral whisker pad were unchanged (Figure 1C).

### 3.2. Rapamycin reduces mechanical and cold allodynia in a mouse model of orofacial neuropathic pain

**Figure 2:**
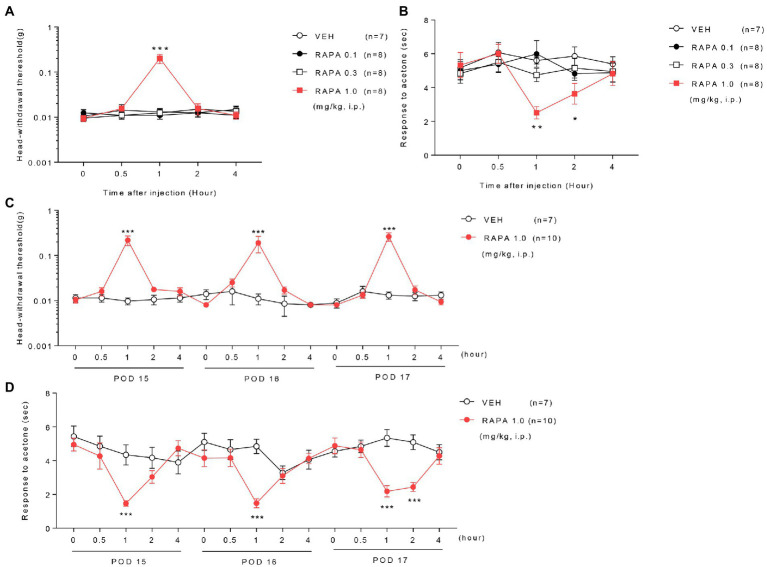
Effect of rapamycin on mechanical and cold allodynia in infraorbital nerve-injured mice. Low or medium doses of rapamycin (RAPA, 0.1 or 0.3 mg/kg) did not relieve mechanical and cold allodynia, whereas high-dose rapamycin (1 mg/kg) significantly ameliorated both mechanical **(A)** and cold allodynia **(B)** 1–2 h after injection compared with those in the vehicle (VEH)-treated group on postoperative day (POD) 14 in ION-pNL mice **(A,B)**. *p < 0.05, **p < 0.01, ***p < 0.001 as compared with the VEH group, *n* = 7–8 per group. Daily treatments with high-dose rapamycin for 3 days (POD 15–17) produced a transient antiallodynic effect in both mechanical and cold allodynia tests, whereas both the maximal effect at 1 h post injection each day and basal response values before the next injection were unchanged **(C,D)**. *p < 0.05, **p < 0.01, ***p < 0.001 as compared with the VEH group, *n* = 7 for RAPA group and *n* = 10 for VEH group.

Treatment with low (0.1 mg/kg) or medium doses (0.3 mg/kg) of rapamycin (RAPA) did not relieve mechanical and cold allodynia compared with the vehicle (VEH)-treated group on postoperative day (POD) 14 in ION-pNL mice (Figures 2A,B). In contrast, high-dose rapamycin (1 mg/kg) significantly relieved mechanical allodynia at 1 h (*p* < 0.0001) and cold allodynia at 1–2 h after injection (*p* = 0.0012, *p* = 0.0398, Figures 2A,B). Daily treatment with rapamycin for 3 days (POD 15–17) produced a transient antiallodynic effect in both mechanical and cold allodynia tests (*p* values <0.0001), but the maximal effect of rapamycin at 1 h post injection on each of the 3 days and the basal response values before the next injection (Figures 2C,D) remained unchanged.

### 3.3. Rapamycin does not change the expression of mTOR signaling proteins in the TNC

**Figure 3:**
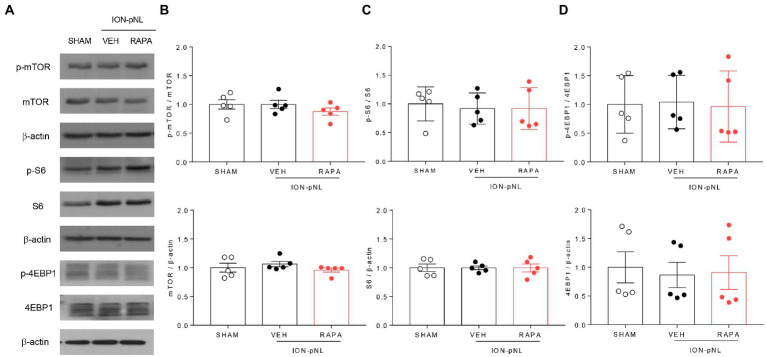
Expression of mTOR signaling-related proteins in the trigeminal nucleus caudalis (TNC). mTOR, S6, 4EBP1, and their phosphorylated forms (p-mTOR, p-S6, and p-4EBP1) were all unchanged in the ION-pNL group compared with the SHAM group **(A–D)**. High-dose rapamycin treatment (RAPA) produced no significant change in the expression of each protein **(A–D)** compared with the VEH group, *n* = 5 per group.

At 1 h after injection of rapamycin or vehicle, the expression of mTOR, S6, 4EBP1, and their phosphorylated forms (p-mTOR, p-S6, and p-4EBP1, respectively) was measured in the TNC tissue. mTOR, S6, 4EBP1, and their phosphorylated forms were all unchanged in the ION-pNL group compared with those in the SHAM group, and no significant change was observed in the expression levels of all these proteins in the rapamycin-treated group (Figures 3A–D).

### 3.4. Rapamycin reduces the expression of S6 and p-S6 proteins in the TG

**Figure 4:**
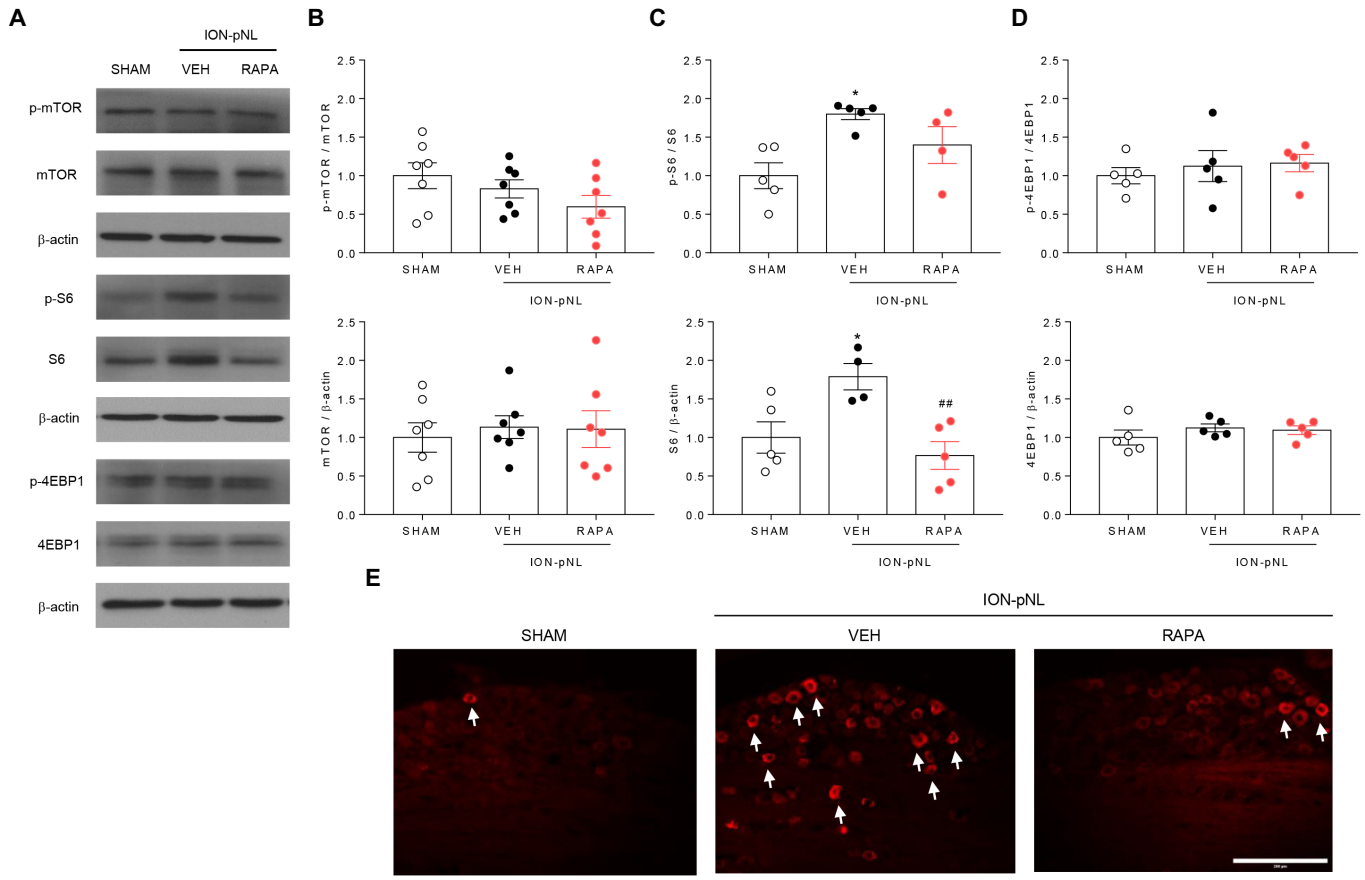
Expression of p-mTOR signaling-related proteins in the trigeminal ganglion (TG). The expression of mTOR, 4EBP1, and their phosphorylated forms (p-mTOR and p-4EBP1) did not change in the ION-pNL group compared with the SHAM group, and the RAPA group also showed no significant change compared with the VEH-treated group **(A,B,D)**. Conversely, the expressions of S6 and p-S6 were significantly increased in the ION-pNL group compared with the SHAM group (*p < 0.05), and rapamycin reduced the increased expressions of S6 and p-S6 in the TG **(A,C)**. ##*p* < 0.01 compared with VEH. Immunohistochemistry also showed an increase in the expression of p-S6 in the ipsilateral TG of the ION-pNL group compared with the SHAM group, and this increased p-S6 expression was reduced by rapamycin treatment **(E)**, The arrows indicate representative p-S6-immunoreactive cells. *n* = 4–7 per group. Scale bar = 200 μm.

We investigated whether rapamycin treatment itself induced changes in mTOR and its downstream proteins in the TG. The expression of p-mTOR, mTOR, p-4EBP1, and 4EBP1 did not change in the ION-pNL group compared with the SHAM group, and rapamycin had no effect on their expression compared with the VEH-treated group (Figures 4A,B,D). However, the expression of S6 and p-S6 was markedly increased in the ION-pNL group (*p* = 0.0442 and *p* = 0.0122, respectively), and rapamycin restored the increased expression of S6 and p-S6 in the TG 14 days after ION-pNL (*p* = 0.0097, Figure 4C). Similar to the western blot results, immunohistochemistry showed a significant increase in the expression of p-S6 in the ipsilateral TG of the ION-pNL group compared with the SHAM group, and this increased p-S6 expression was reduced by rapamycin treatment (Figure 4E).

### 3.5. Rapamycin attenuates the ION-pNL-induced increase in Iba-1 expression in the TNC

**Figure 5:**
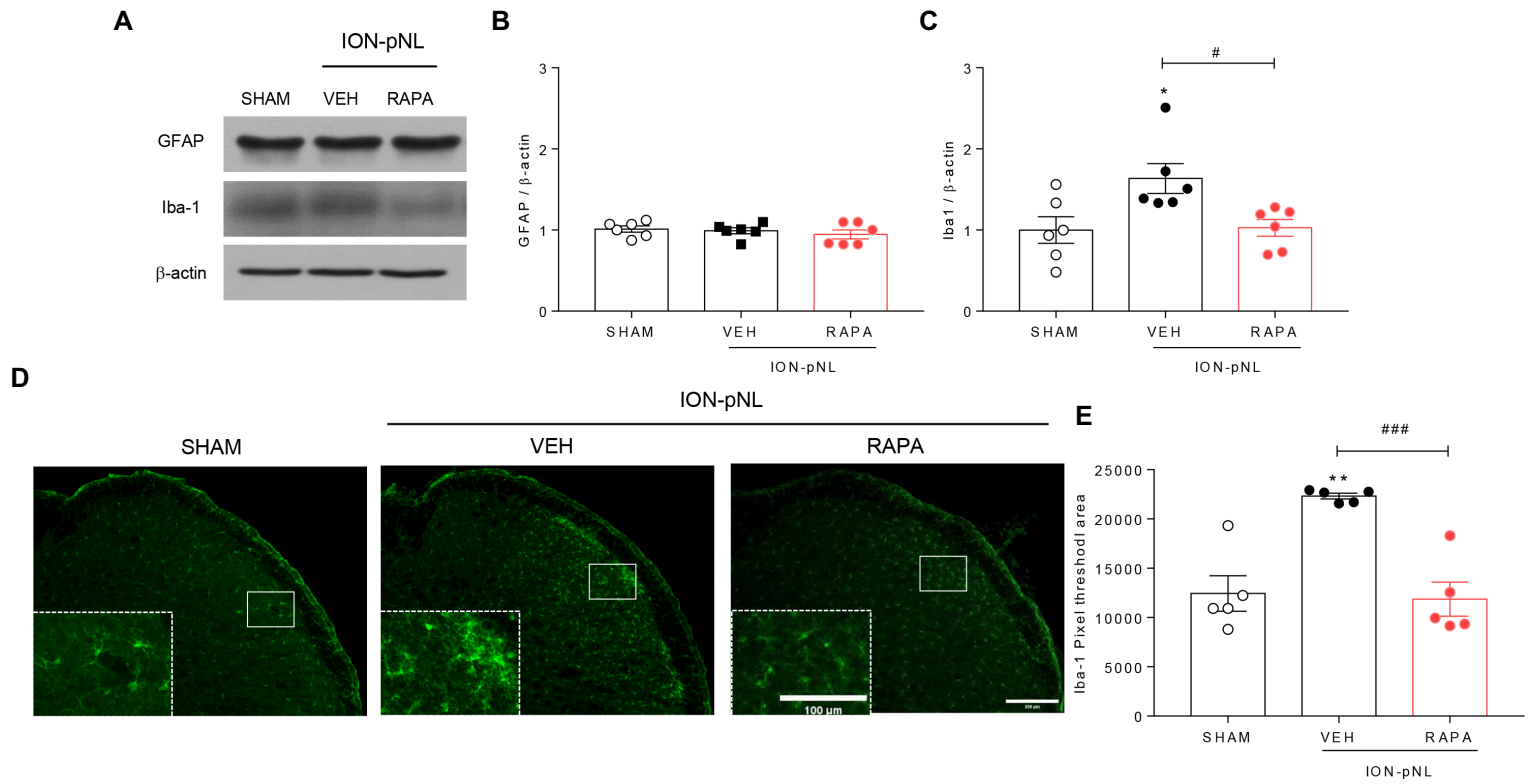
Effect of rapamycin on GFAP and Iba-1 expression in the TNC. Western blot analysis showing that the expression level of GFAP, an astrocyte marker, did not change in the ION-pNL group compared with the SHAM group, and the RAPA group also showed no significant change compared with the VEH-treated group **(A,B)**. In contrast, Iba-1, a microglia marker, was significantly increased in the ION-pNL group compared with the SHAM group (*p < 0.05), and increased Iba-1 was abrogated by rapamycin treatment **(A,C)**. #*p* < 0.05 compared with VEH. Immunohistochemistry revealing increased Iba-1 expression in the ipsilateral TNC of the ION-pNL group **(D,E)**. **p < 0.01 compared with SHAM. In addition, rapamycin treatment restored the increased Iba-1-immunoreactive pixel area in the TNC of the ION-pNL group **(D,E)**. ###*p* < 0.001 compared with VEH, *n* = 5–6 per group. Scale bar = 200 μm, 100 μm in enlarged picture.

In the SHAM and ION-pNL groups, changes in GFAP and Iba-1 expressions in the TNC were examined. Western blot analysis revealed that the expression of GFAP, an astrocyte marker, was unchanged in the ION-pNL group compared with the SHAM group, and the rapamycin-treated group also showed no significant change compared with the VEH-treated group (Figures 5A,B). In contrast, Iba-1, a microglia marker, was significantly increased in the ION-pNL group (*p* = 0.0325), and this increase was subsequently attenuated by rapamycin treatment (*p* = 0.0419, Figures 5A,C). Immunohistochemistry revealed that the expression of Iba-1 was significantly increased in the ipsilateral TNC of the ION-pNL group compared with the SHAM group (*p* = 0.0013). In addition, rapamycin treatment restored the increased Iba-1-immunoreactive pixel area in the TNC of the ION-pNL group (*p* = 0.0008, Figures 5D,E).

### 3.6. Rapamycin reduces p-p38 MAPK expression but not p-JNK or p-ERK expression in the TNC

**Figure 6:**
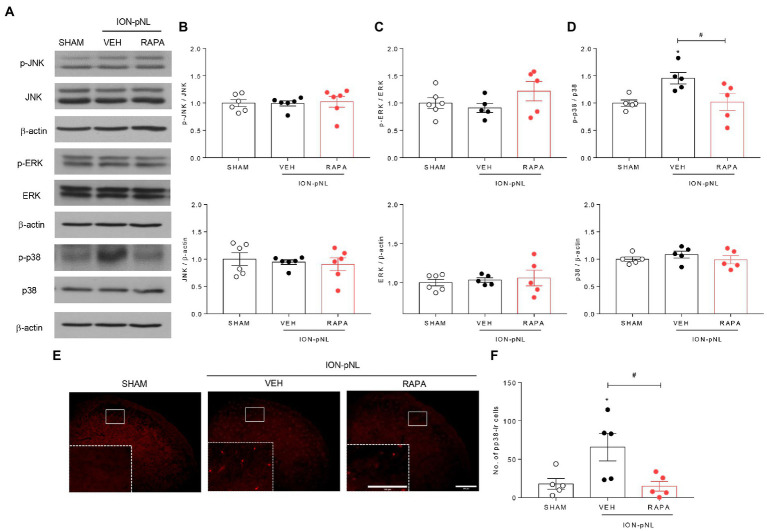
Effect of rapamycin on MAPK family expression in the TNC. Western blot analysis showed that both p-JNK and p-ERK expression did not change in the ION-pNL group compared with the SHAM group, and RAPA also showed no effect on p-JNK and p-ERK expression compared with the VEH-treated group **(A–C)**. On the other hand, the expression of p-p38 significantly increased in the TNC of the ION-pNL group (*p < 0.05 compared with SHAM), and RAPA completely reduced the increased p-p38 expression compared with VEH-treated mice **(A,D)**. #*p* < 0.05 compared with the VEH group, *n* = 5 per group. Immunohistochemistry revealed an increase in p-p38 expression induced by ION-pNL, which was also significantly reduced in the RAPA group **(E,F)**. *p < 0.05 and #*p* < 0.05 compared with the SHAM or VEH group, respectively, *n* = 5 per group. Scale bar = 200 μm, 100 μm in enlarged picture.

The effect of intraperitoneal rapamycin treatment on p-JNK, p-ERK, and p-p38 MAPK expression in the ipsilateral TNC region is shown in Figure 6. Western blot analysis revealed that both p-JNK and p-ERK expressions were unchanged in the ION-pNL group compared with the SHAM group, and rapamycin treatment also had no effect on p-JNK and p-ERK expressions compared with the VEH-treated group (Figures 6A–C). In contrast, the expression of p-p38 was significantly increased in the TNC of the ION-pNL group (*p* = 0.0351), and rapamycin completely reduced the increased p-p38 expression compared with VEH-treated mice (*p* = 0.0135, Figures 6A,D). This inhibitory effect of rapamycin on the increase in p-p38 MAPK expression induced by ION-pNL was also confirmed in subsequent immunohistochemistry studies. Similar to the results presented in Figures 6A,D, the increase in p-p38 expression induced by ION-pNL (*p* = 0.0410) was significantly decreased in the rapamycin-treated group (*p* = 0.0293, Figures 6E,F).

### 3.7. P-p38 MAPK is colocalized with Iba-1 but not NeuN or GFAP in the TNC

**Figure 7:**
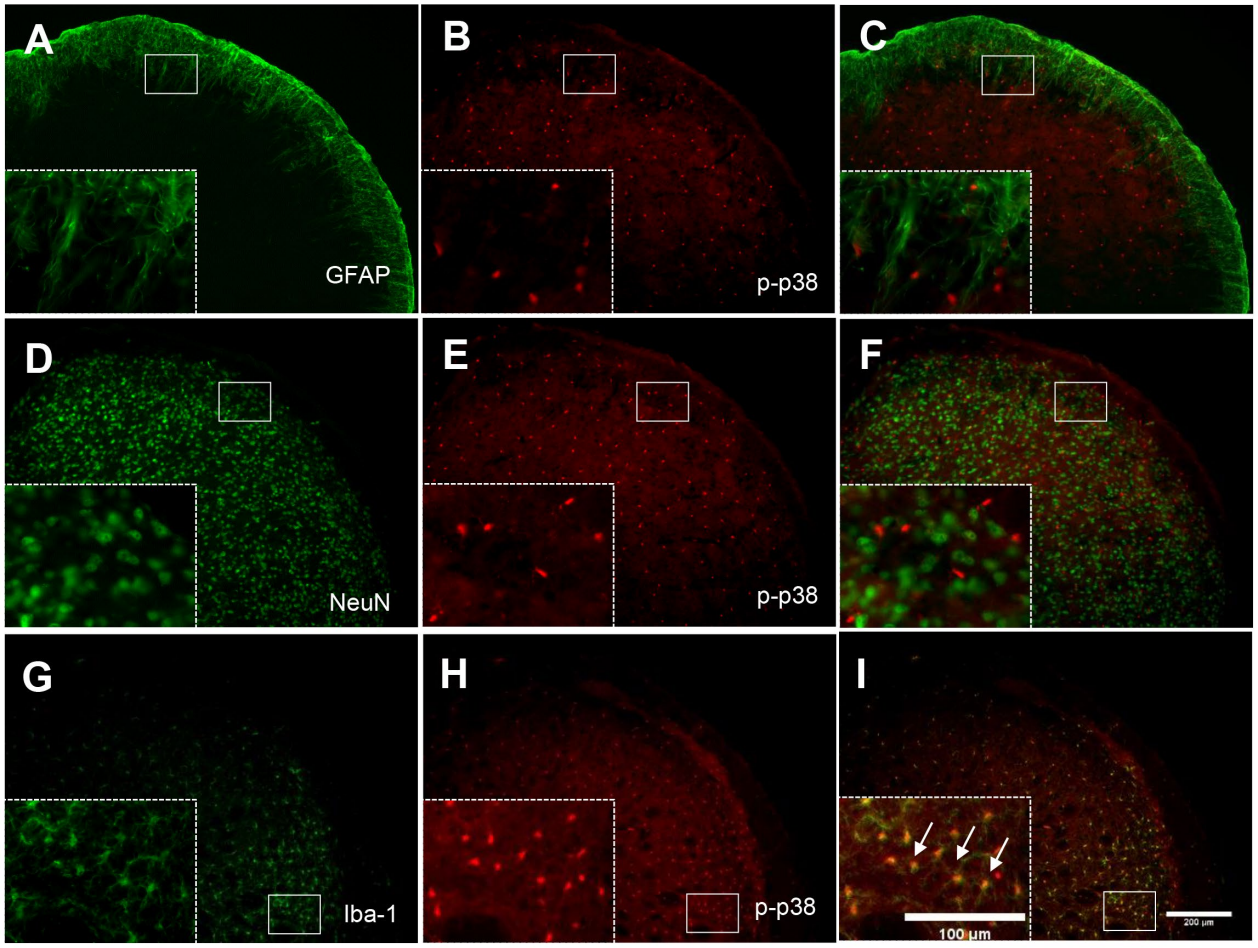
Colocalization of GFAP, NeuN, or Iba-1 with p-p38 in the TNC. Neither GFAP **(A–C)** nor NeuN **(D–F)** was colocalized with p-p38 in the TNC. In contrast, Iba-1 immunoreactivity was visualized in p-p38-positive cells in the TNC of the ION-pNL group **(G–I)**. The rectangular areas are magnified from each panel **(A–I)** and arrows indicate representative p-p38-immunoreactive cells contained for Iba-1. Scale bar = 200 μm, 100 μm in enlarged picture.

To determine the cellular distribution of p-p38 MAPK, double immunohistochemical staining of p-p38 MAPK with different cell markers (GFAP, NeuN, and Iba-1) was performed. As shown in Figure 7, p-p38-immunoreactive cells were primarily colocalized with the microglial marker Iba-1 (Figures 7G–I) but not with the astrocytic marker GFAP (Figures 7A–C) or neuronal marker NeuN (Figures 7D–F). These results indicate that the increase in p-p38 MAPK is primarily induced in the TNC microglia in ION-pNL mice.

### 3.8. P-MKK4 is involved in the phosphorylation of p38 MAPK

**Figure 8:**
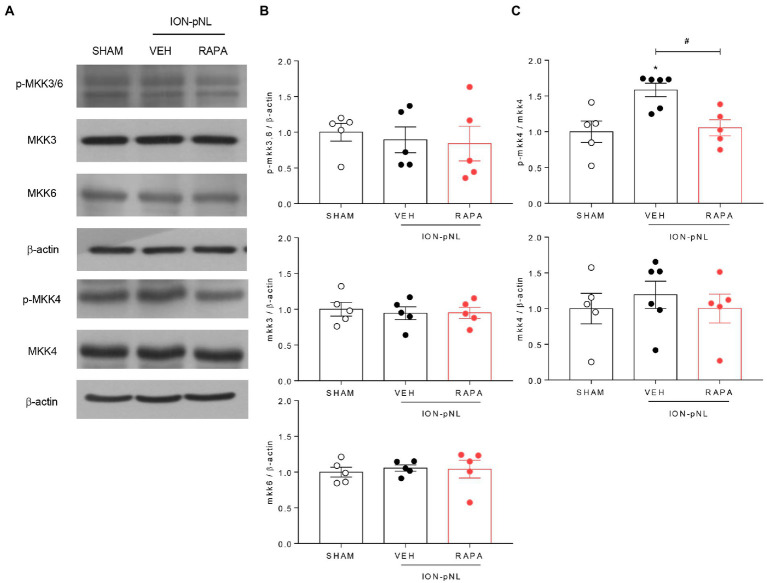
Effect of rapamycin on MKK3, MKK6, and MKK4 in the TNC. Both p-MKK3 and p-MKK6 expressions were unchanged in the ION-pNL group compared with the SHAM group, and RAPA had no effect on p-MKK3 and p-MKK6 expressions compared with the VEH-treated group **(A,B)**. On the other hand, p-MKK4 expression significantly increased in the ION-pNL group **(A,C)**, and RAPA restored the increased p-MKK4 expression after ION-pNL **(A,C)**. *p < 0.05 and #*p* < 0.05 compared with the SHAM group and VEH group, respectively, *n* = 5–6 per group.

The dual specificity kinases MKK3 and MKK6 appear to be primarily responsible for the activation of p38 in most cells; MKK4 also activates p38 in some instances. To determine which MKKs regulate p38 under trigeminal neuropathic conditions, changes in MKK3/6 and MKK4 in the ipsilateral TNC were evaluated *via* western blot analysis. The expression of MKK3, MKK6, and p-MKK3/6 did not change in the ION-pNL group compared with the SHAM group (Figures 8A,B). In contrast, the expression of p-MKK4 was significantly increased in the ION-pNL group (*p* = 0.0106), and this increase was completely restored in the RAPA group (*p* = 0.0202, Figures 8A,C).

## 4. Discussion

In the present study, we demonstrated that rapamycin exerts significant antiallodynic effects in a mouse model of chronic trigeminal neuropathic pain induced by ION injury. Rapamycin was initially discovered as an antifungal metabolite produced by *Streptomyces hygroscopicus* in a soil sample from Easter Island. Subsequently, it was found to exert immunosuppressive and antiproliferative properties in mammalian cells (Li et al., 2014). Rapamycin has also been reported as a potent inhibitor of S6K1, a serine/threonine kinase activated by a variety of agonists (Price et al., 1992) and an important mediator of PI3 kinase signaling (Chung et al., 1994). Recent studies have indicated that rapamycin can be used to treat cancer and various other diseases, such as diabetes, obesity, neurological diseases, and certain genetic disorders (Guertin and Sabatini, 2005; Shillingford et al., 2006; Qian et al., 2008; Li et al., 2014). Because rapamycin modulates protein synthesis and protein translation processes, including autophagy, its potential for chronic pain control has been documented in several animal models (Weragoda et al., 2004; Jimenez-Diaz et al., 2008). For example, Duan et al. (2018) reported that systemic injection of rapamycin (5 mg/kg) inhibited mechanical allodynia resulting from oxaliplatin-induced neuropathic pain. Wang et al. (2016) also reported that intrathecal rapamycin treatment (10 μg) improved mechanical hyperalgesia caused by spinal cord injury in rats. Similar to these studies, we demonstrated that intraperitoneal treatment with high-dose rapamycin (1 mg/kg) exerted significant antiallodynic effects in orofacial pain assessments of mechanical and cold stimulation. The maximum impact of rapamycin on mechanical and cold allodynia occurred within 1 h post injection and gradually returned to baseline levels over a 1-h observation period (Figures 2A,B). Following repeated administration of rapamycin for 3 days, a similar pattern of antiallodynic effects was observed in mechanical and cold allodynia tests. Of note, the maximal effect of rapamycin neither increased nor decreased 1 h after injection, and no change in baseline pain response was observed (Figures 2C–D). These results indicate that the antiallodynic effect of rapamycin is transient and short-lived, and tolerance does not occur, even when rapamycin is repeatedly administered daily for 3 days.

The mTOR signaling pathway regulates cell growth, proliferation, and synaptic plasticity by controlling protein synthesis downstream of multiple stimuli, such as nutrients, energy metabolism, and growth factors (Hay and Sonenberg, 2004; Ma and Blenis, 2009). Recent studies have shown that mTOR signaling plays an important role in the development and progression of chronic pain (Li et al., 2015; Lutz et al., 2015; Cho et al., 2018). A number of studies have demonstrated that rapamycin inhibits the activation of the downstream targets of mTORC1, such as p-S6 and p-4EBP1, and ameliorates the increased mechanical hypersensitivity associated with local inflammation or neuropathic pain (Price et al., 2007; Jimenez-Diaz et al., 2008; Geranton et al., 2009; Norsted Gregory et al., 2010). However, in the present study, ION-pNL surgery did not alter the level of p-mTOR or the phosphorylation of its downstream targets (p-S6 and p-4EBP1) in the TNC. Moreover, in the ION-pNL group, no changes in the expression of p-mTOR or its downstream effectors (p-S6 and p-4EBP1) were detected 1 h after rapamycin treatment. These findings are interesting because several studies previously reported that mTOR signaling-related proteins are upregulated in the spinal cord dorsal horn or dorsal root ganglion under chronic pain conditions. Ma et al. (2020) reported that increased phosphorylation of mTOR, S6, and 4EBP1 was observed at 7 days after surgery in the spinal cord of mice with chronic constriction injury (CCI)-induced neuropathy. In addition, Liang et al. (2013) showed that the levels of p-mTOR and p-S6K1 were significantly increased in the ipsilateral L4/5 spinal cord at 2 h, 1 day, 3 days, and 7 days after intraplantar complete Freund’s adjuvant injection (chronic inflammatory pain model). However, they did not verify the changes in p-mTOR-related proteins 14 days after nerve injury or inflammation. Furthermore, Liang et al. (2013) found that spinal nerve ligation (SNL) did not alter p-mTOR or p-S6K1 levels in the ipsilateral L5 spinal cord within the first 2 weeks after surgery. These findings indicate that whether mTOR and its downstream signaling proteins are activated in the spinal cord after peripheral nerve injury remains controversial and also suggest that unlike peripheral neuropathy pain in the lower limbs, trigeminal neuropathy pain may not mediate changes in mTOR signaling in the TNC.

We further investigated whether rapamycin treatment itself induced changes in mTOR and its downstream proteins in the TG. We confirmed that the expressions of p-mTOR, mTOR, p-4EBP1, and 4EBP1 did not change in the ION-pNL group compared with the SHAM group, and rapamycin had no effect on their expression compared with the VEH-treated group. However, the expression of S6 and p-S6 markedly increased in the ION-pNL group, and rapamycin restored this increased expression of S6 and p-S6 in the TG 14 days after ION-pNL (Figure 4C). These findings open up two possibilities: (1) the injected dose of rapamycin normally acts on the mTOR downstream signaling pathway in mice with trigeminal neuropathic pain and (2) p-mTOR expression levels in the TG or TNC are already at their peak in SHAM mice. Because of the latter possibility, both p-mTOR and mTOR expressions might not be upregulated in ION-pNL mice. In addition, it raises another question as to how changes in S6 and p-S6 expressions in the TG directly or indirectly affect microglia activation together with p38 MAPK phosphorylation in the TNC. Further in-depth studies are required to answer these questions.

Glial cells, including astrocytes and microglia, play important and unique roles in the regulation of synaptic function and nerve excitability, which contribute to the formation and development of neuropathic pain (Watkins et al., 2001). Activated glial cells cause hypertrophy with thick branches and enlarged cell bodies. An increase in the number of activated microglia and astrocytes in the superficial dorsal horn was observed in several neuropathic pain models (Gwak and Hulsebosch, 2009; Vallejo et al., 2010; Tenorio et al., 2013). In particular, several animal models of trigeminal nerve injury revealed that activation of glial cells in the TNC results in the development of trigeminal neuropathic pain. Piao et al. (2006) found that microglia in the superficial laminae of the medullary dorsal horn (MDH) are activated and maintained for 14 days following inferior alveolar nerve and mental nerve transection (IAMNT) in rats. Xu et al. (2008) also demonstrated that ION-pNL in mice induces mechanical allodynia with activation of microglia in the ipsilateral MDH. Although rapamycin did not alter glial expression in the TNC after orofacial formalin injection in our previous study, we found that Iba-1 protein levels in the TNC were significantly increased in the ION-pNL group compared with the sham surgery group and that rapamycin treatment reduced this increased Iba-1 expression in the TNC (Figures 5A,C–E). In contrast, the expression of GFAP-immunoreactive cells did not change 14 days after ION-pNL surgery, and treatment with rapamycin did not affect GFAP expression (Figures 5A,B). These results suggest that the antiallodynic effect of high-dose rapamycin (1 mg/kg) is mediated by activated microglia but not astrocytes in the TNC.

On the other hand, a previous study showed that microglia in the TNC were activated on day 1 and restored on day 8 after ION partial ligation, whereas GFAP expression was increased on day 8 (Xu et al., 2008). They suggested that microglia are activated and recovered earlier than astrocytes following trigeminal nerve injury. These results were different from those of our present study, but Xu et al. did not assess the changes in Iba-1 or GFAP expression at 14 days after ION injury. In addition, Xu et al. directly exposed the right ION 1–2 mm rostral to the infraorbital fissure on the maxillary bone, which was different from our intraoral approach. In this regard, although several studies have reported increases in Iba-1 or GFAP expression in the ipsilateral spinal dorsal horn or TNC after nerve injury, time-dependent discrepancies between animal models still remain (Echeverry et al., 2008, 2011). Tateda et al. (2017) reported that microglia were activated and persisted up to 42 days after spinal cord injury (SCI), whereas GFAP expression was not elevated. In addition, it has been reported that both microglia and astrocytes in the superficial layer of the MDH were activated and maintained in a similar state for 14 days after IAMNT in rats (Piao et al., 2006). These results, along with our study results, suggest that there are time-dependent differences in the expression of each type of glial cell, possibly due to (1) differences in nerve damage sites (e.g., sciatic nerve injury, ION injury, IAMNT, and SCI), (2) differences in the species (rat vs. mouse) or strains of animals used, and (3) differences in surgical methods used in the study (e.g., infraorbital fissure approach vs. intraoral approach).

Spinal microgliosis is a characteristic of microglial activation following peripheral nerve injury (Zhang et al., 2007; Echeverry et al., 2011). Gliosis is a nonspecific reactive change of glial cells in response to injury and often involves the proliferation or hypertrophy of glial cells. Following peripheral nerve injury, in addition to undergoing morphological changes, spinal microglia begin to proliferate within 2 to 3 days and reach maximal levels in 4 to 7 days (Echeverry et al., 2008). Importantly, microglia activation *via* p38 phosphorylation is common following activation of cell-surface receptors on microglia, such as CX3CR1, P2X4, P2X7, P2Y12, and TLR4 (Ji and Suter, 2007; Kobayashi et al., 2008; Trang et al., 2009; Clark et al., 2010; Ji et al., 2013). In addition, activation of p38 MAPK induces the synthesis and release of various inflammatory mediators and promotes the development of neuropathic pain (Ji et al., 2013). Recently, Tateda et al. (2017) also reported that Iba-1-stained microglia in the lumbar spinal cord were significantly decreased in rapamycin-treated mice. Overall, these results suggest that rapamycin treatment plays an important role in inhibiting microglia proliferation, which is closely associated with the regulation of p-p38 MAPK.

The MAPK family includes three major members, i.e., ERK, p38, and JNK, which represent three different signaling cascades. The MAPKs provide a link between extracellular stimuli and intracellular responses (Pearson et al., 2001). MAPKs are involved in various aspects of cell signaling and gene expression in the CNS (Koistinaho and Koistinaho, 2002; Qu et al., 2016). Several studies have demonstrated that MAPK pathways play essential roles in inflammation and tissue remodeling (Khan et al., 2012; Newton and Dixit, 2012). In particular, p38 MAPK is involved in response to various stressful stimuli, including lipopolysaccharide, ultraviolet (UV) light, and inflammatory cytokines (Han et al., 1994; Lee et al., 1994; Gong et al., 2010). p38 MAPK signaling plays a pivotal role in the inflammatory response and is involved in the generation of hyperalgesia and allodynia after peripheral nerve injury (Ji and Suter, 2007; Terayama et al., 2011). Zhang et al. (2017) reported that although there was no change in JNK expression, activated p38 MAPK (p-p38) increased in the TG in unilateral partial ION ligation mice, and this p38 MAPK activation was associated with CXCL13/CXCR5 activation. In addition, Lim et al. (2007) demonstrated that intracisternal administration with PD98059 or SB203580, an MEK inhibitor and p38 MAPK inhibitor, respectively, significantly inhibited infraorbital nerve CCI (ION-CCI)-induced mechanical allodynia in the orofacial area. Of note, microglia p38 MAPK was activated in the MDH following trigeminal nerve injury, and minocycline, a microglia inhibitor, inhibited tactile hypersensitivity and p38 MAPK activation in hyperactive MDH microglia (Piao et al., 2006). In our previous study, we demonstrated that p38 MAPK expression was activated in the TNC, which may be an important factor involved in the processing of acute orofacial inflammatory pain. In the present study, we also observed that p-p38 MAPK protein expression in the TNC, but not p-JNK and p-ERK expression, increased 14 days after ION-pNL injury, and this increased p-p38 returned to baseline 1 h following rapamycin injection (Figure 6). Moreover, our immunohistochemical studies showed that p-p38 MAPK-immunoreactive cells in the TNC only colocalized with Iba-1 and not with NeuN or GFAP (Figure 7). Collectively, these results suggest that an increase in TNC p-p38 MAPK upon microglial activation (as indicated by increased Iba-1 expression) contributes to the development of trigeminal neuropathic pain, which can be transiently inhibited by a single injection of rapamycin.

In general, MAPKs require dual phosphorylation on threonine and tyrosine residues to become activated, which is conducted by the MAPKs (MKK or MEK) (Han et al., 1996). p38 MAPK can also be phosphorylated and activated by several MAPKs, including MKK3, MKK6, and MKK4 (Derijard et al., 1995; Han et al., 1996; Raingeaud et al., 1996). Ma et al. (2007) described a distinct role of MKK3-mediated p38 signaling in renal apoptosis and early inflammatory response in obstructed kidneys. Xie et al. (2019) reported that the inhibition of the MKK3/6-p38 signaling pathway significantly reduced esophageal cancer cell growth and patient-derived esophageal tumor growth *in vivo*. Sorkin et al. (2009) also reported that MKK3 is required for the normal development of chronic pain behavior and phosphorylation of spinal cord p38. On the other hand, our western blot data revealed that increased phosphorylation of MKK4 in the TNC but not MKK3/6 was induced by ION-pNL surgery, and this increased p-MKK4 was reduced in rapamycin-treated mice (Figures 8A,C). Thus, it is important to address whether MKK3 and MKK6 are essential for tumor necrosis factor-stimulated p38 MAPK activation. UV-stimulated p38 MAPK activation can be mediated by MKK3, MKK4, and MKK6 (Brancho et al., 2003). Chen et al. (2009) also reported GluR6-containing kainate receptors that modulate p38 MAPK activation through a signaling cascade including MLK3, MKK3/MKK6, and MKK4 in rat hippocampal CA1 during brain ischemia injury. Moreover, Guan et al. (1998) demonstrated that SEK1/MKK4 can act as an upstream kinase pathway that activates both p38 MAPK and JNK/SAPK and induce the upregulation of cyclooxygenase-2 expression and prostaglandin E2 synthesis. This discrepancy between studies, including our results, appears to be due to differences in specific p38 isoforms. MKK3 and MKK6 phosphorylate most p38 isoforms *in vitro*, whereas selective activation and substrate specificity were observed *in vivo* (Enslen et al., 1998). MKK4 has been reported to specifically phosphorylate p38α and p38δ (Jiang et al., 1997; Han et al., 2020). Therefore, we believe that MKK4 plays a role in the regulation of p38 MAPK, especially the p38α and p38δ isoforms, in the TNC microglia, which contributes to the induction of trigeminal neuropathic pain. In addition, the antiallodynic effect of rapamycin may be mediated by p38 MAPK inhibition *via* the regulation of MKK4 but not MKK3/6. However, the underlying mechanisms through which rapamycin treatment can directly or indirectly modulate MKK4 phosphorylation warrant further study.

Several studies have reported that sex differences appear in several rodent models of chronic pain conditions and that females may be more sensitive to nociceptive stimuli. For example, tactile allodynia was more prominent and/or more persistent in female rodents in several neuropathic pain models, such as CCI (Vacca et al., 2014), partial SNL (Coyle et al., 1995), and complete SNL (Taiwo et al., 2005). In addition, several studies have shown that male and female rodents have similar pain expression patterns, but the underlying mechanisms may be different (Sorge et al., 2015; Mapplebeck et al., 2018; Inyang et al., 2019). These gender differences may be due to differences in the immune system, especially in females who need more immune flexibility to allow pregnancy. While microglia are well known to play an important role in the development of chronic pain in males, several studies have shown that T lymphocytes infiltrating the spinal cord can mediate neuropathic pain in females (Sorge et al., 2015). Taves et al. (2016) reported that spinal inhibition of p38 MAPK with the highly selective p38 inhibitor skepinone reduces inflammatory and neuropathic pain in male mice but not in female mice. Because our present study is also closely associated with microglia activation, including p38 MAPK phosphorylation in the TNC, it is important to address whether rapamycin can reduce orofacial mechanical and cold allodynia in female mice with trigeminal neuropathic pain. In this regard, we attempted experiments to validate the analgesic efficacy of rapamycin in an orofacial neuropathic pain model in female mice. However, the facial mechanical allodynia test was not possible because female C57BL/6 mice are more aggressive than male mice and too sensitive to perform stimulus-evoked response tests. This can be due to hormonal influences of the estrous cycle of females. This may be a limitation of orofacial pain research, but the issue can be addressed in future studies by developing a new protocol to detect orofacial pain in female mice.

In conclusion, we demonstrated that rapamycin treatment reduced both mechanical and cold facial allodynia in mice with trigeminal neuropathic pain. Both p-p38 MAPK and p-MKK4 were significantly increased in ION-pNL mice and downregulated following rapamycin treatment. This increased p-p38 expression colocalized with microglia but not neurons or astrocytes. These findings suggest that the systemic injection of rapamycin reduces orofacial neuropathic pain and that this antiallodynic effect is closely associated with the inhibition of p-MKK4/p-p38 MAPK-mediated microglial activation in the TNC.

## Data availability statement

The original contributions presented in the study are included in the article/Supplementary material, further inquiries can be directed to the corresponding author.

## Ethics statement

The animal study was reviewed and approved by The Kyung Hee University Institutional Animal Care and Use Committee.

## Author contributions

J-HY and D-HR performed all experiments, designed the study, analyzed the data, and drafted the manuscript. D-HR completed the manuscript. All authors contributed to the article and approved the submitted version.

## Funding

This work was supported by the National Research Foundation of Korea (NRF) grants funded by the Korean government (MSIP) (no. NRF-2022R1F1A1073652, 2021R1I1A1A0105813912).

## Conflict of interest

The authors declare that the research was conducted in the absence of any commercial or financial relationships that could be construed as a potential conflict of interest.

## Publisher’s note

All claims expressed in this article are solely those of the authors and do not necessarily represent those of their affiliated organizations, or those of the publisher, the editors and the reviewers. Any product that may be evaluated in this article, or claim that may be made by its manufacturer, is not guaranteed or endorsed by the publisher.
