# Supplementary figures and images for: The mTOR inhibitor rapamycin suppresses trigeminal neuropathic pain and p-MKK4/p-p38 mitogen-activated protein kinase-mediated microglial activation in the trigeminal nucleus caudalis of mice with infraorbital nerve injury
Source: Front Mol Neurosci. 2023 Apr 14;16:1172366. doi: 10.3389/fnmol.2023.1172366 (PMC10140572; doi:10.3389/fnmol.2023.1172366)

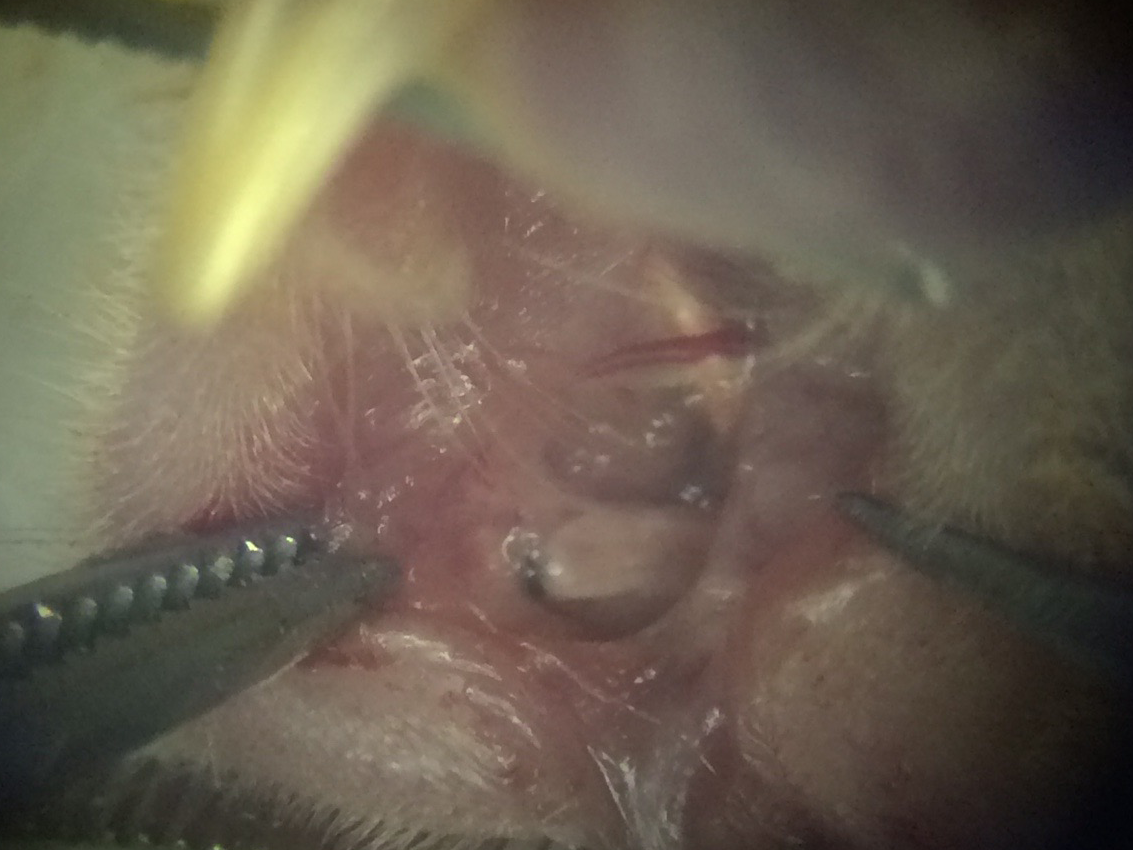

Supplement: Supplementary file 1 [file Data_Sheet_1_v1.ZIP › Figure 1/Figure 1B.post-surgery.tif]

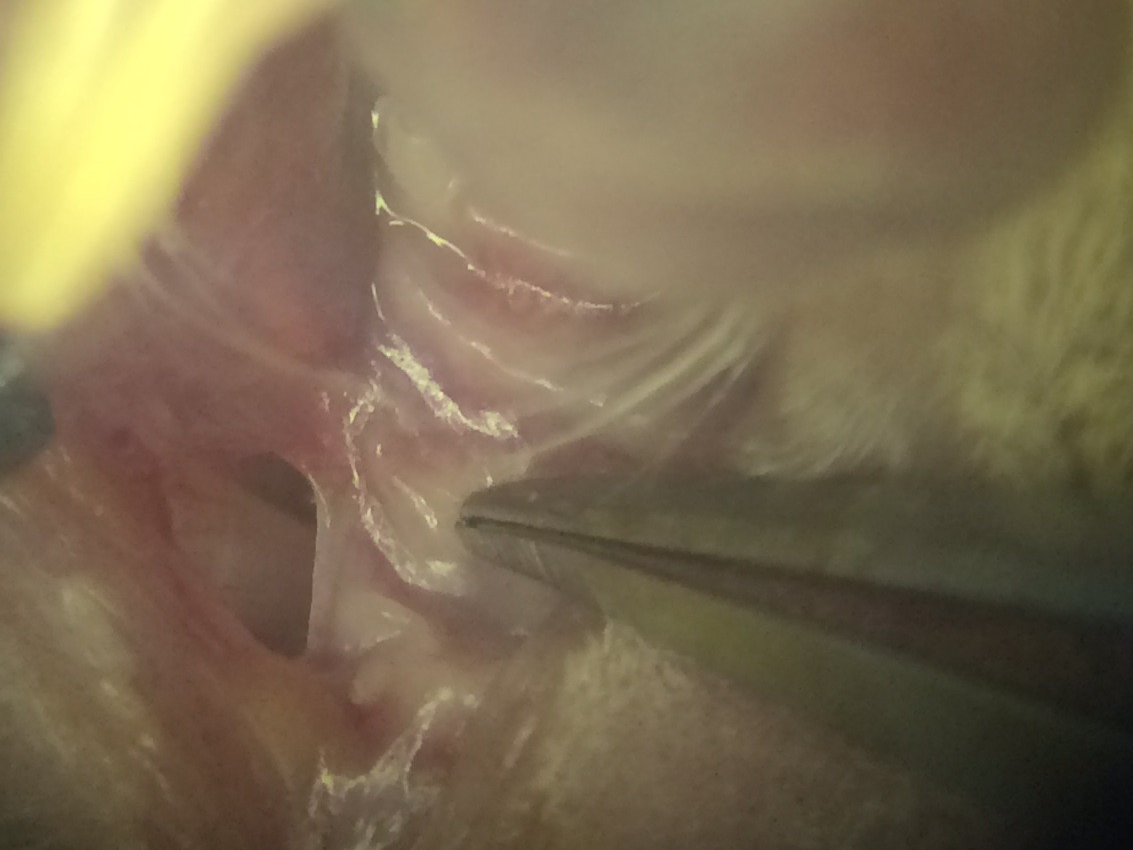

Supplement: Supplementary file 1 [file Data_Sheet_1_v1.ZIP › Figure 1/Figure 1A.pre-surgery.tif]

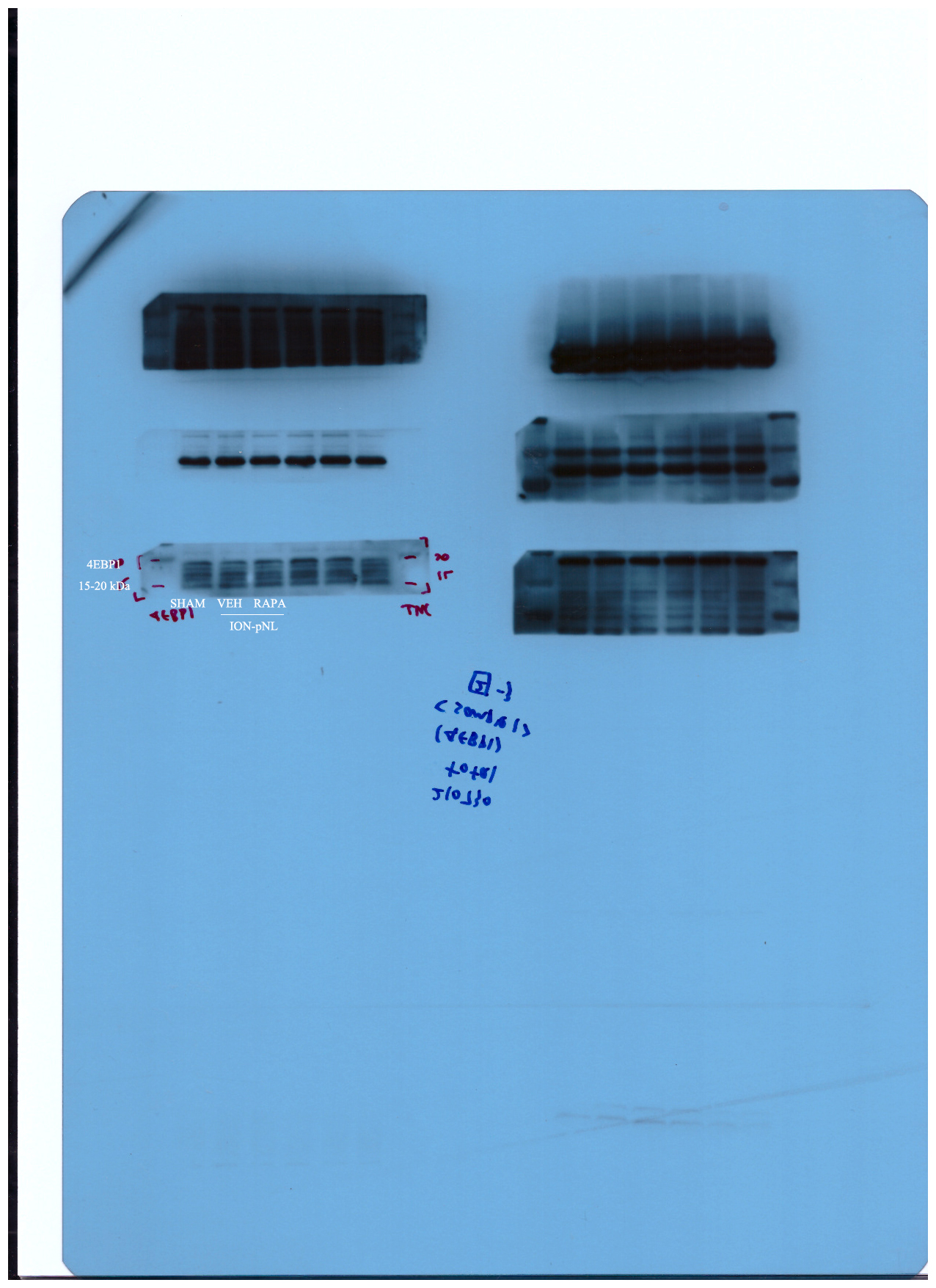

Supplement: Supplementary file 3 [file Data_Sheet_3_v1.ZIP › Figure 3/Figure 3A,D_4EBP1.jpg]

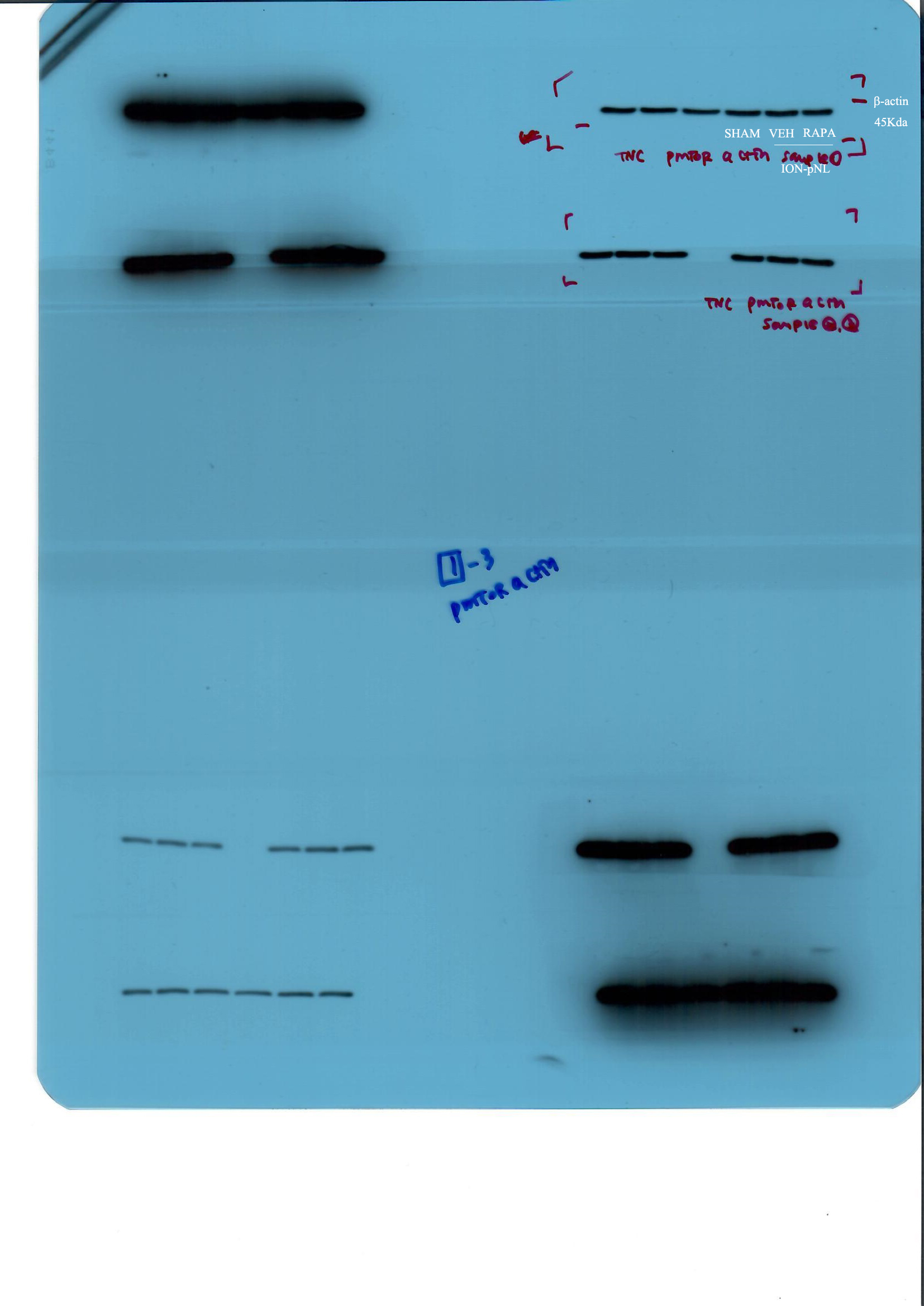

Supplement: Supplementary file 3 [file Data_Sheet_3_v1.ZIP › Figure 3/Figure 3A,B_actin of mTOR,p-mTOR.jpg]

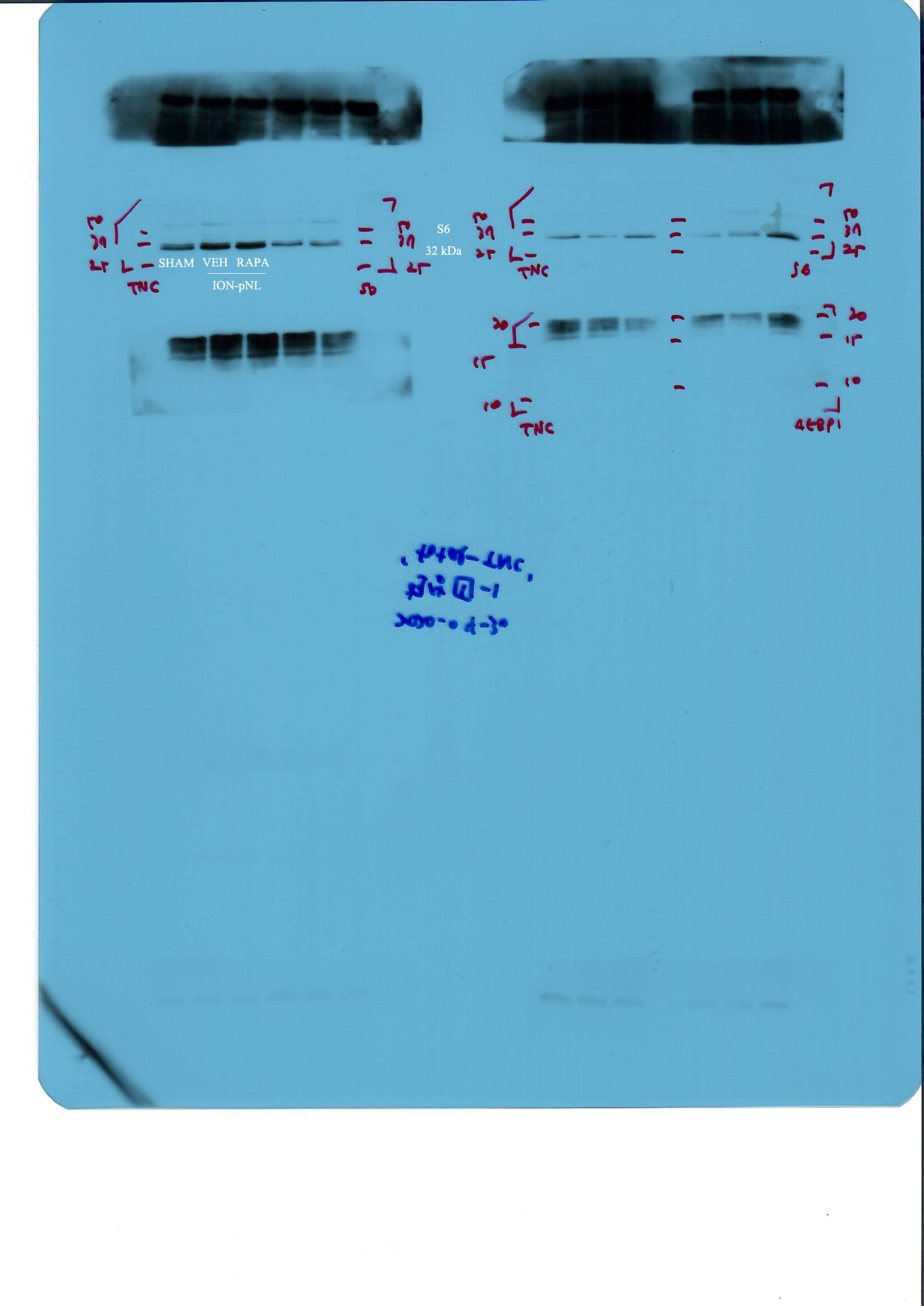

Supplement: Supplementary file 3 [file Data_Sheet_3_v1.ZIP › Figure 3/Figure 3A,C_S6.jpg]

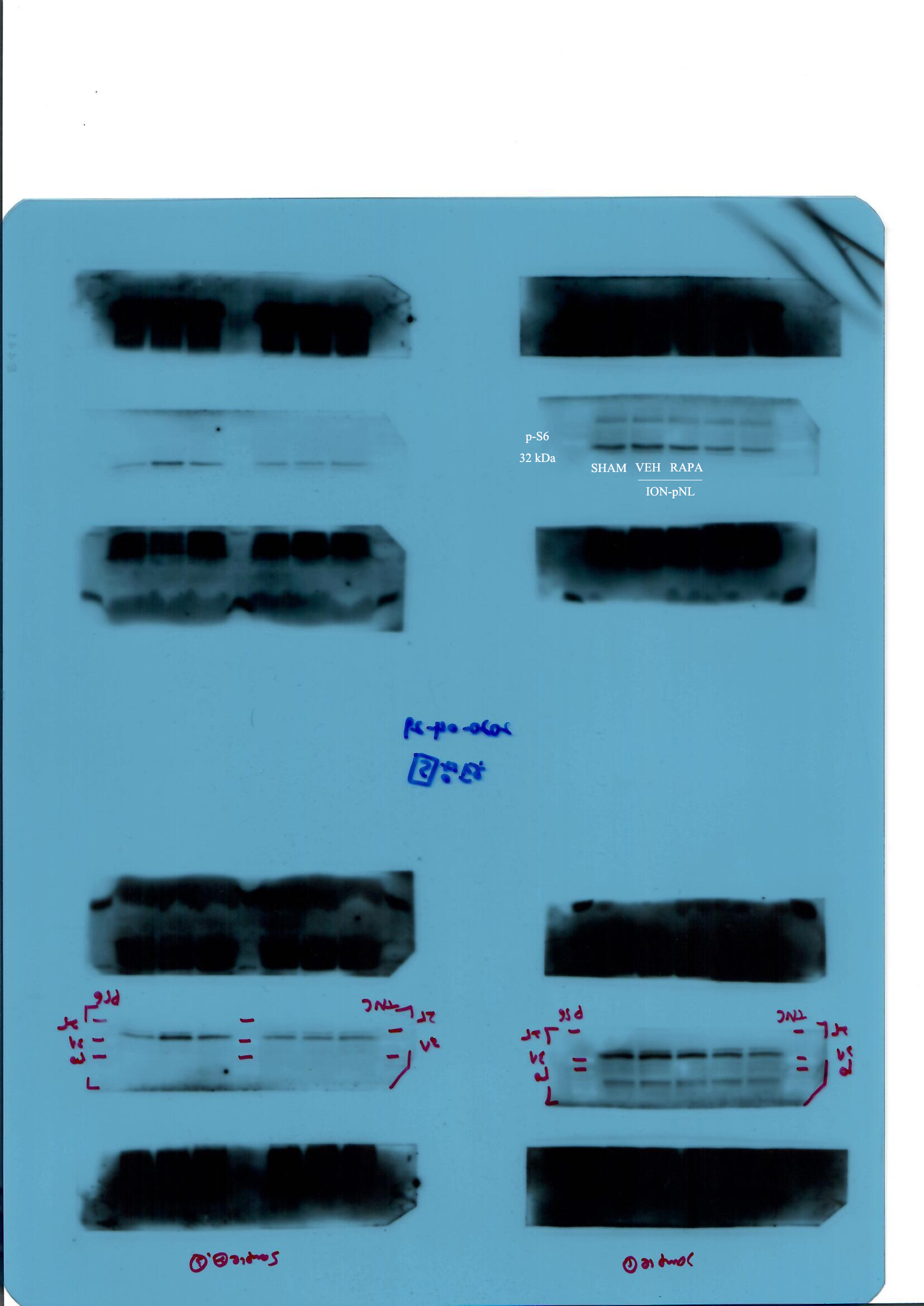

Supplement: Supplementary file 3 [file Data_Sheet_3_v1.ZIP › Figure 3/Figure 3A,C_p-S6.jpg]

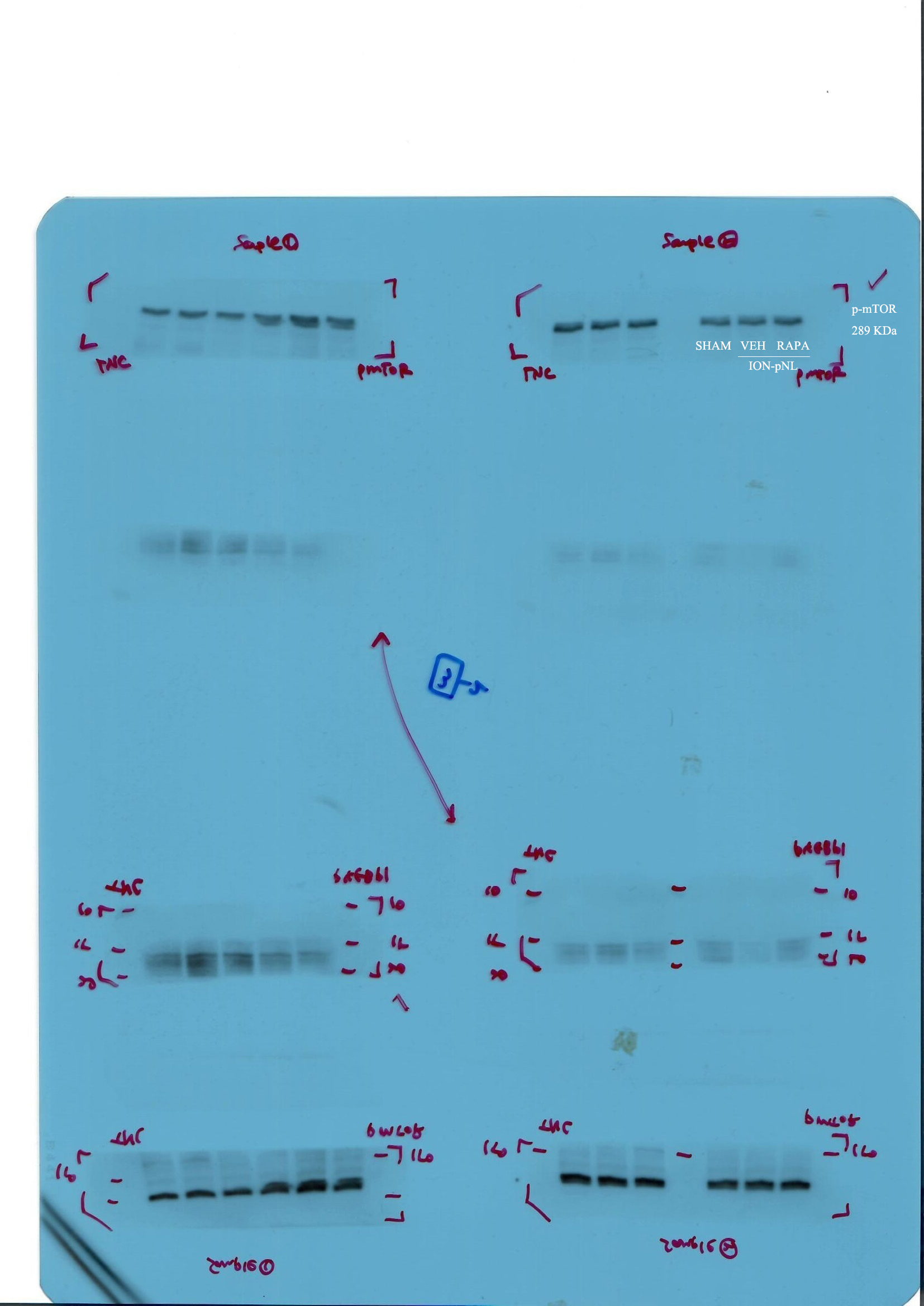

Supplement: Supplementary file 3 [file Data_Sheet_3_v1.ZIP › Figure 3/Figure 3A,B_p-mTOR.jpg]

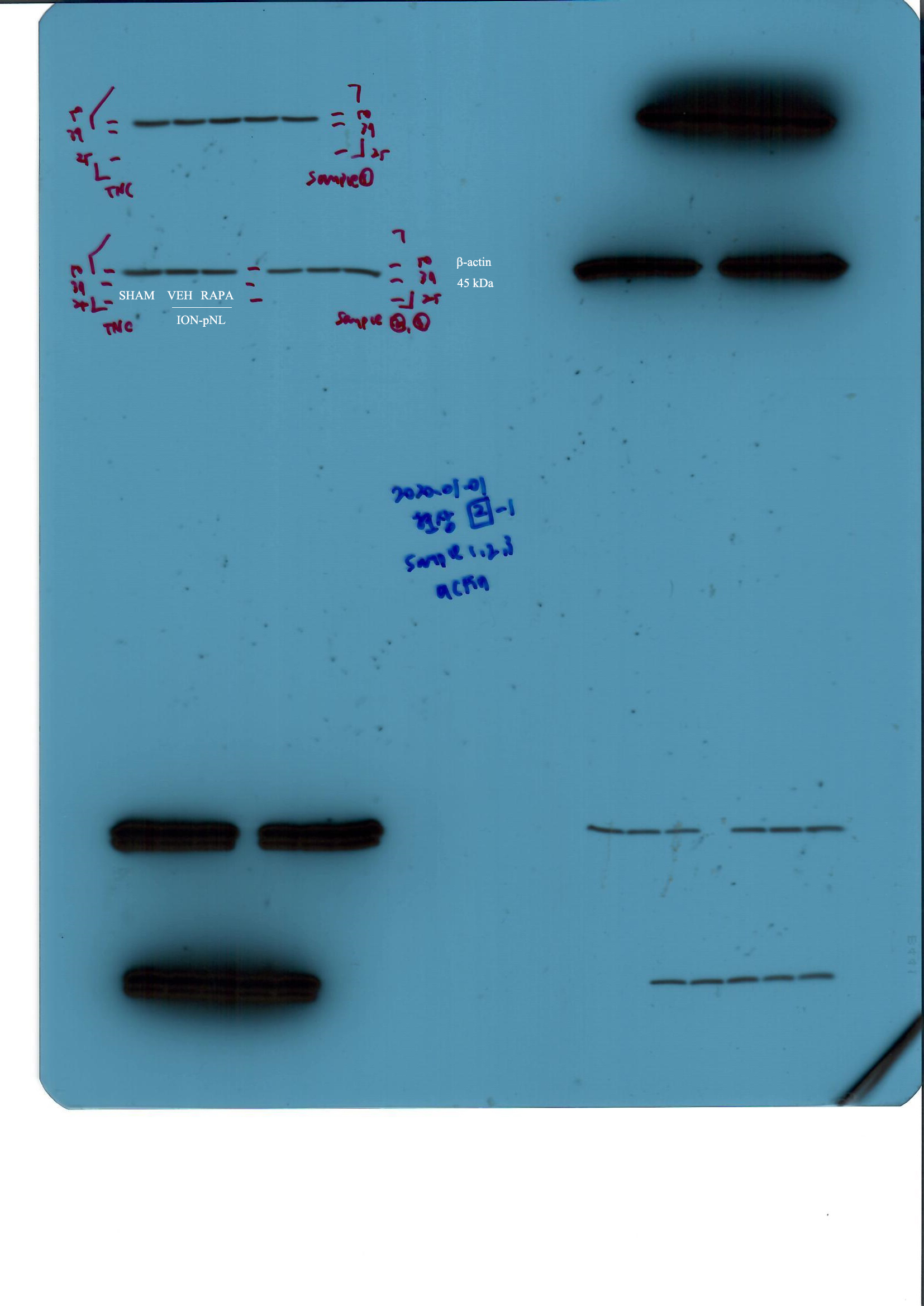

Supplement: Supplementary file 3 [file Data_Sheet_3_v1.ZIP › Figure 3/Figure 3A,C_actin of S6,p-S6.jpg]

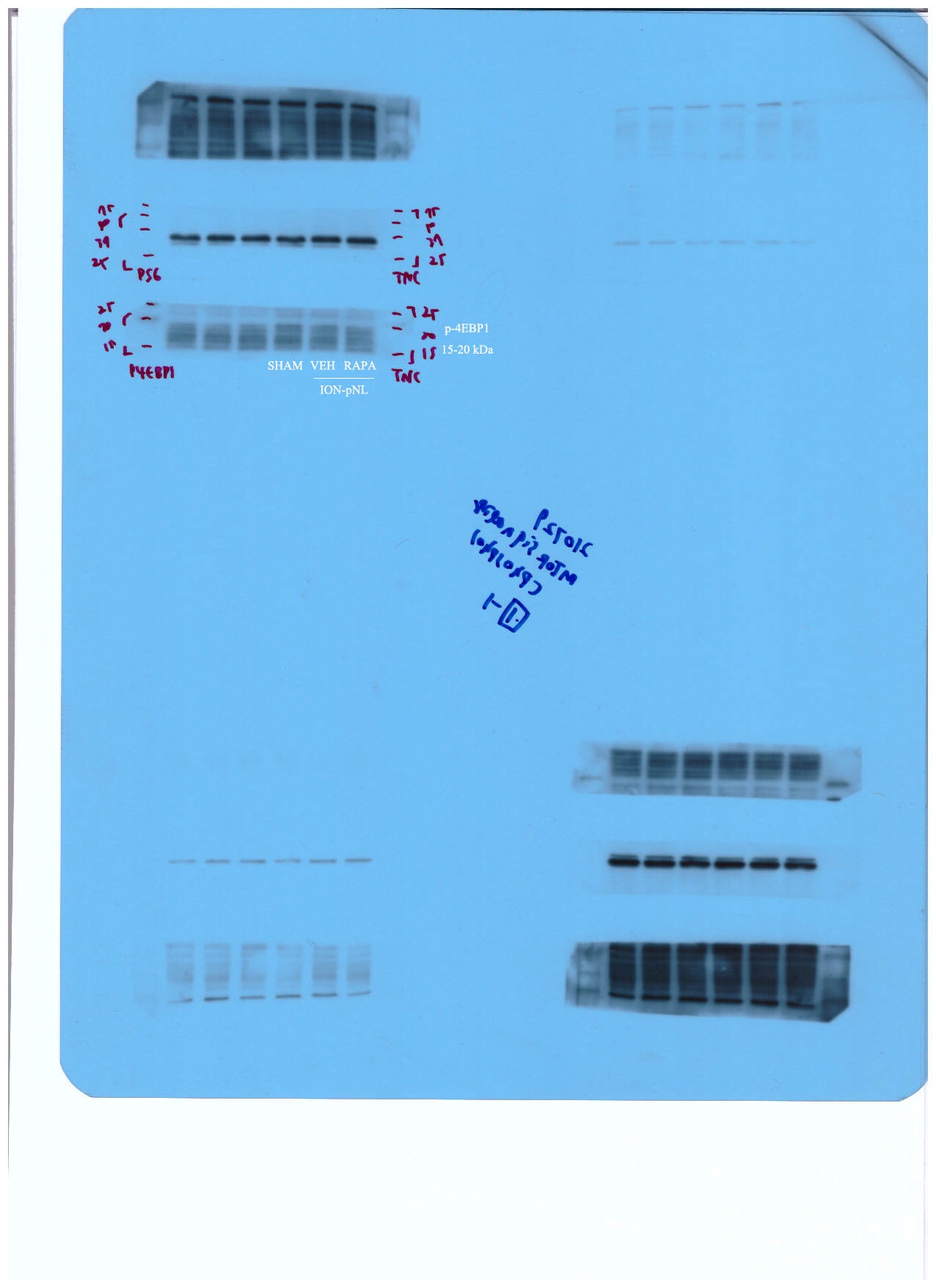

Supplement: Supplementary file 3 [file Data_Sheet_3_v1.ZIP › Figure 3/Figure 3A,D_p-4EBP1.jpg]

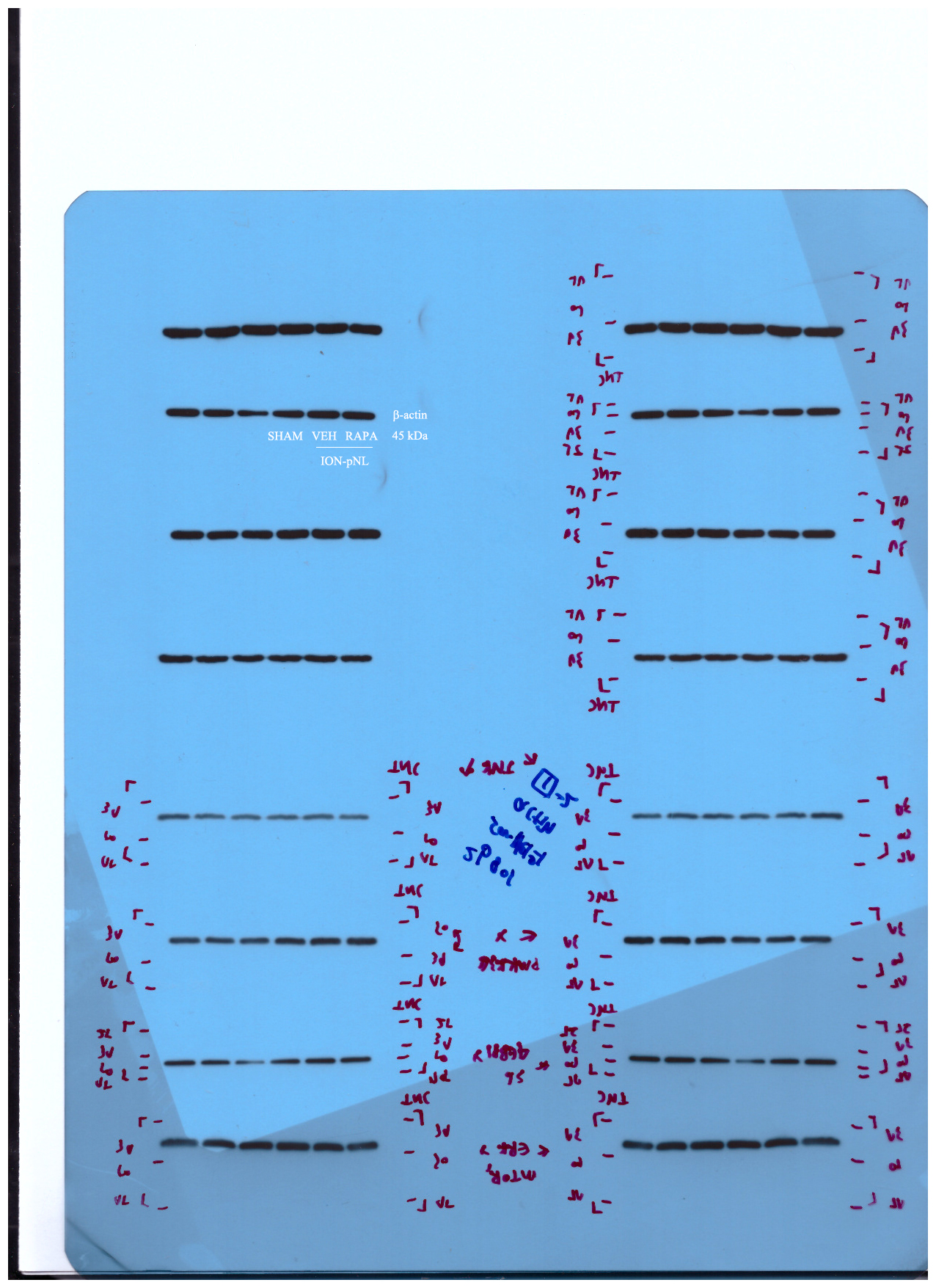

Supplement: Supplementary file 3 [file Data_Sheet_3_v1.ZIP › Figure 3/Figure 3A,D_actin of 4EBP1,p-4EBP1.jpg]

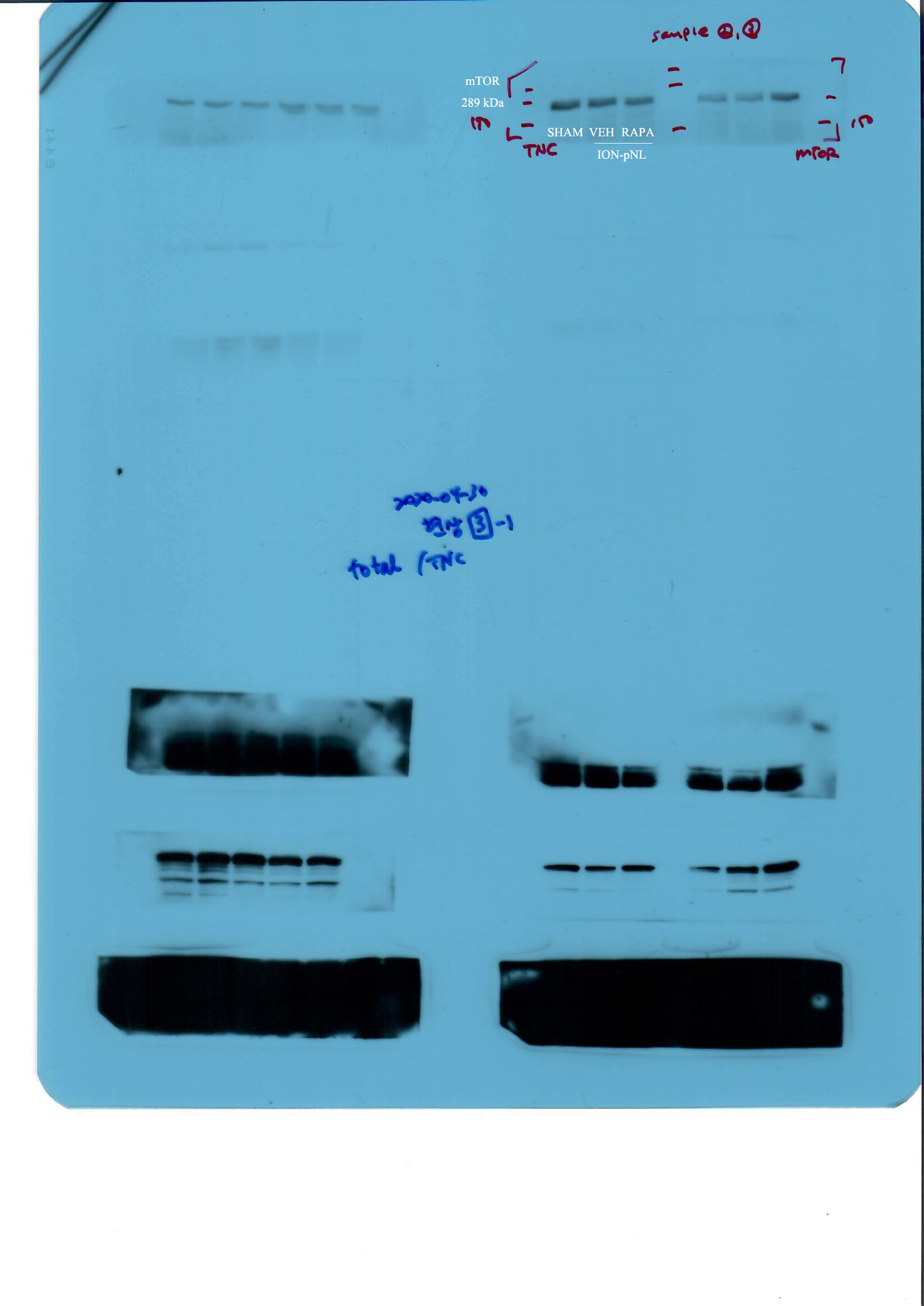

Supplement: Supplementary file 3 [file Data_Sheet_3_v1.ZIP › Figure 3/Figure 3A,B_mTOR.jpg]

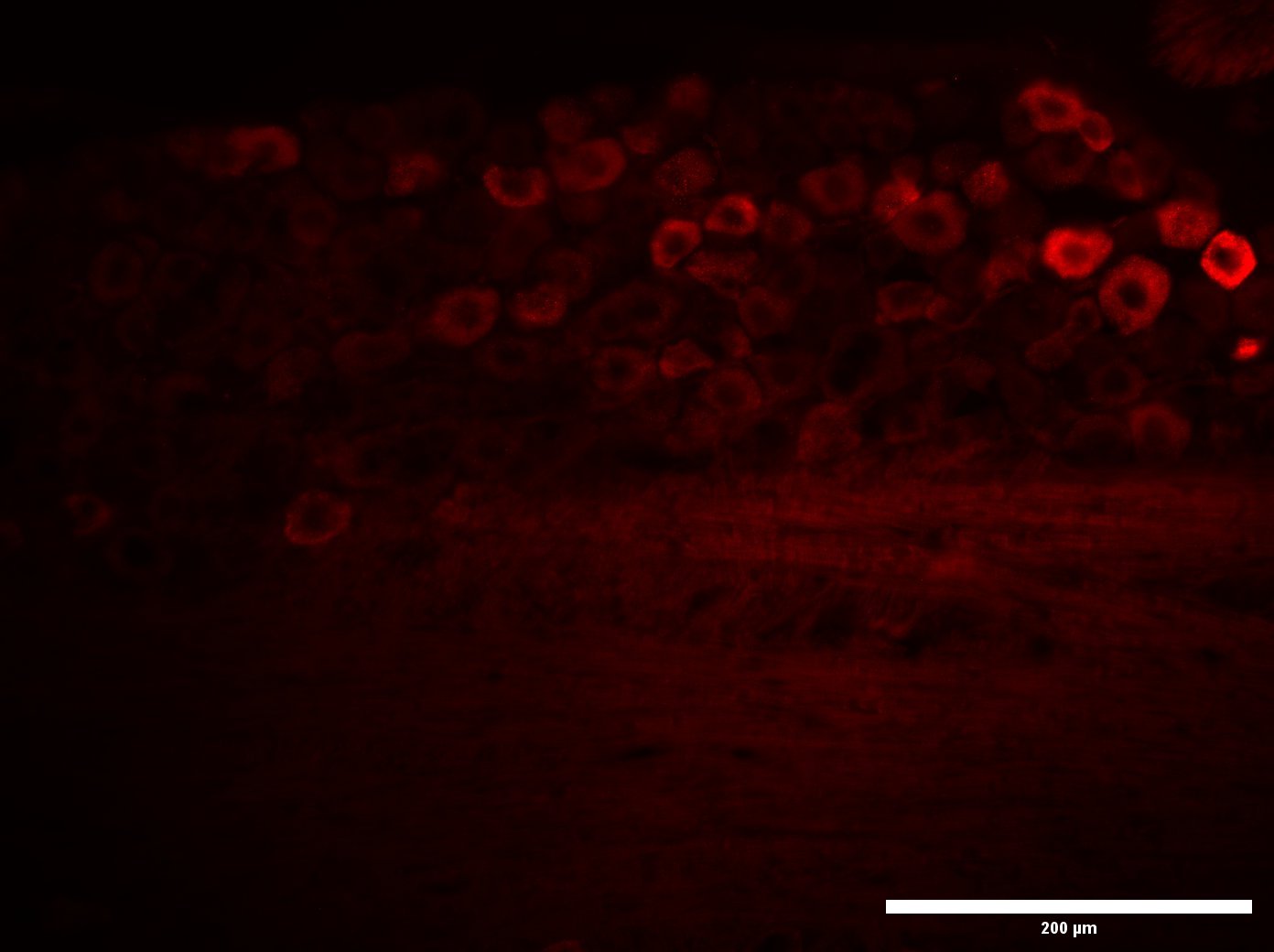

Supplement: Supplementary file 4 [file Data_Sheet_4_v1.ZIP › Figure 4/RAPA_scal bar.jpg]

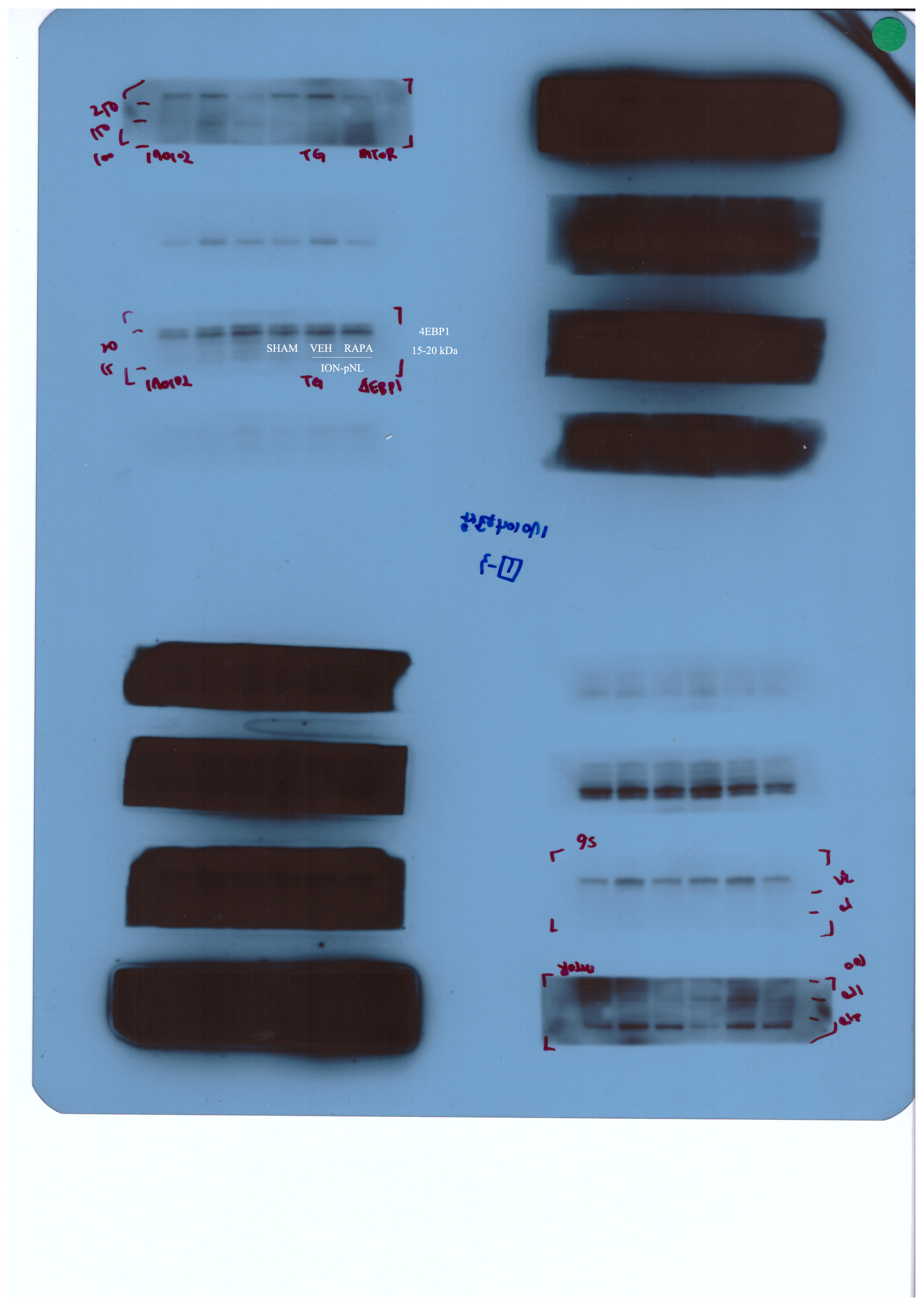

Supplement: Supplementary file 4 [file Data_Sheet_4_v1.ZIP › Figure 4/Figure 4A,D_4EBP1.jpg]

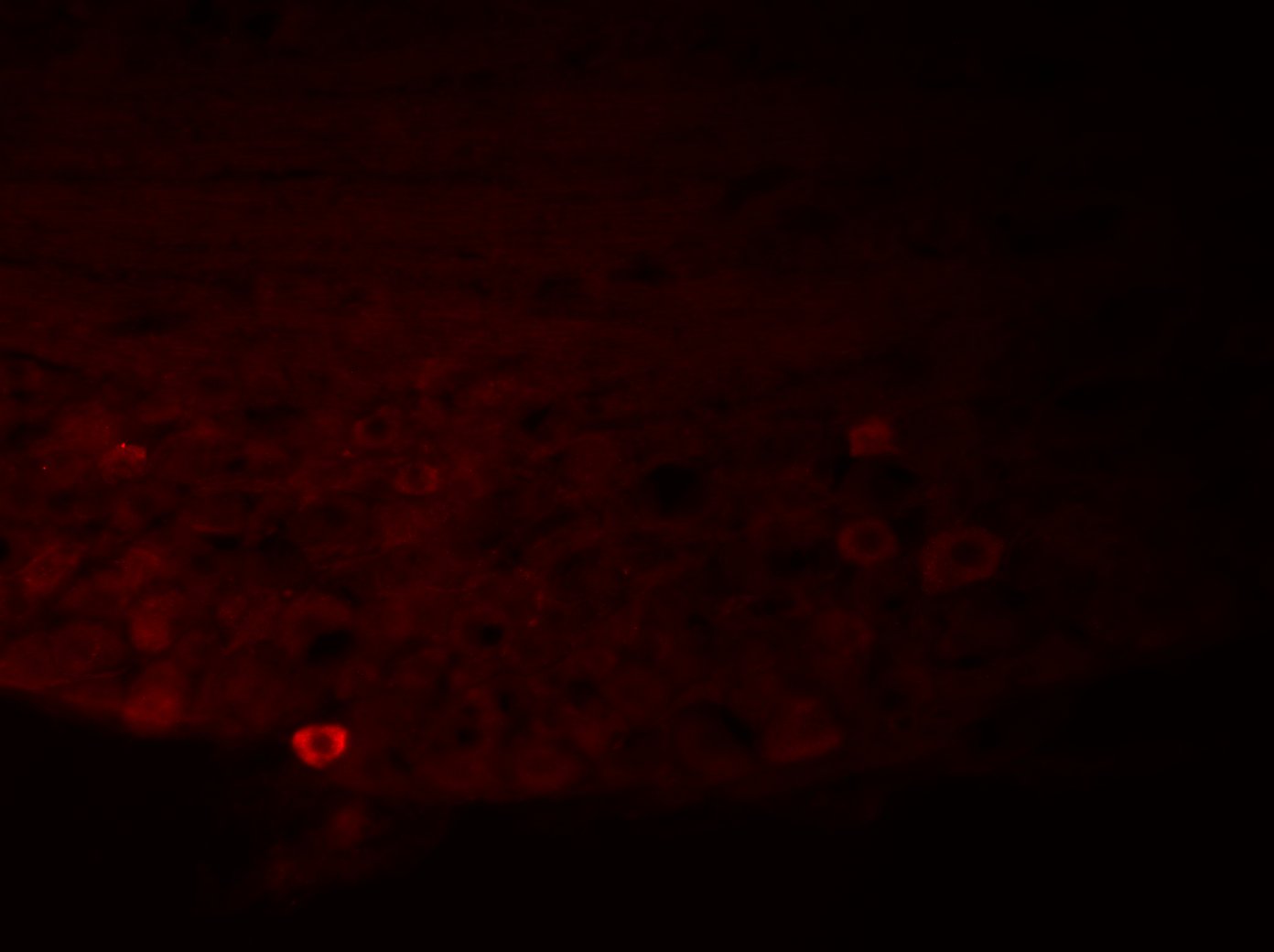

Supplement: Supplementary file 4 [file Data_Sheet_4_v1.ZIP › Figure 4/Figure 4E_SHAM.jpg]

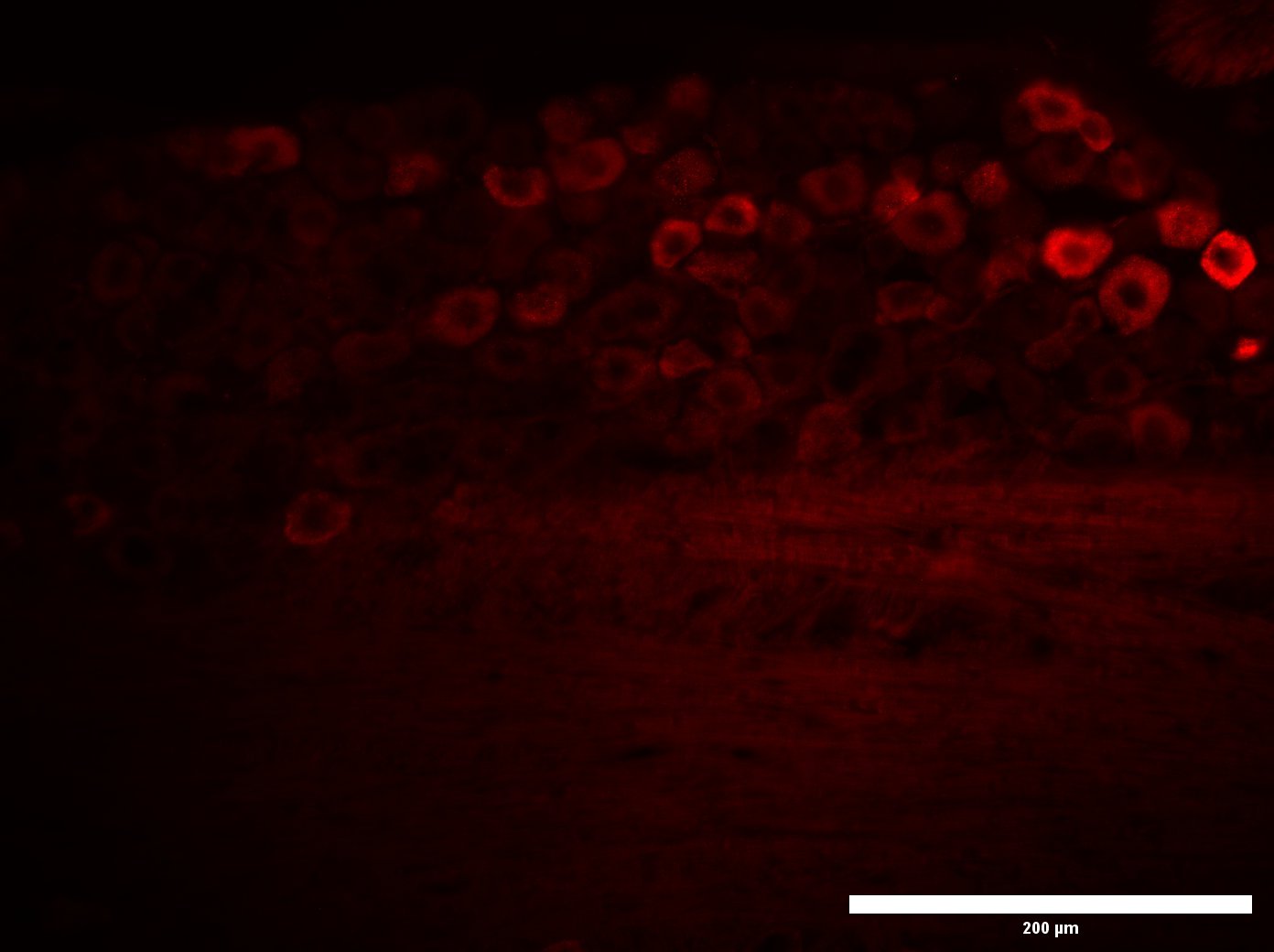

Supplement: Supplementary file 4 [file Data_Sheet_4_v1.ZIP › Figure 4/RAPA_scal bar_blod.jpg]

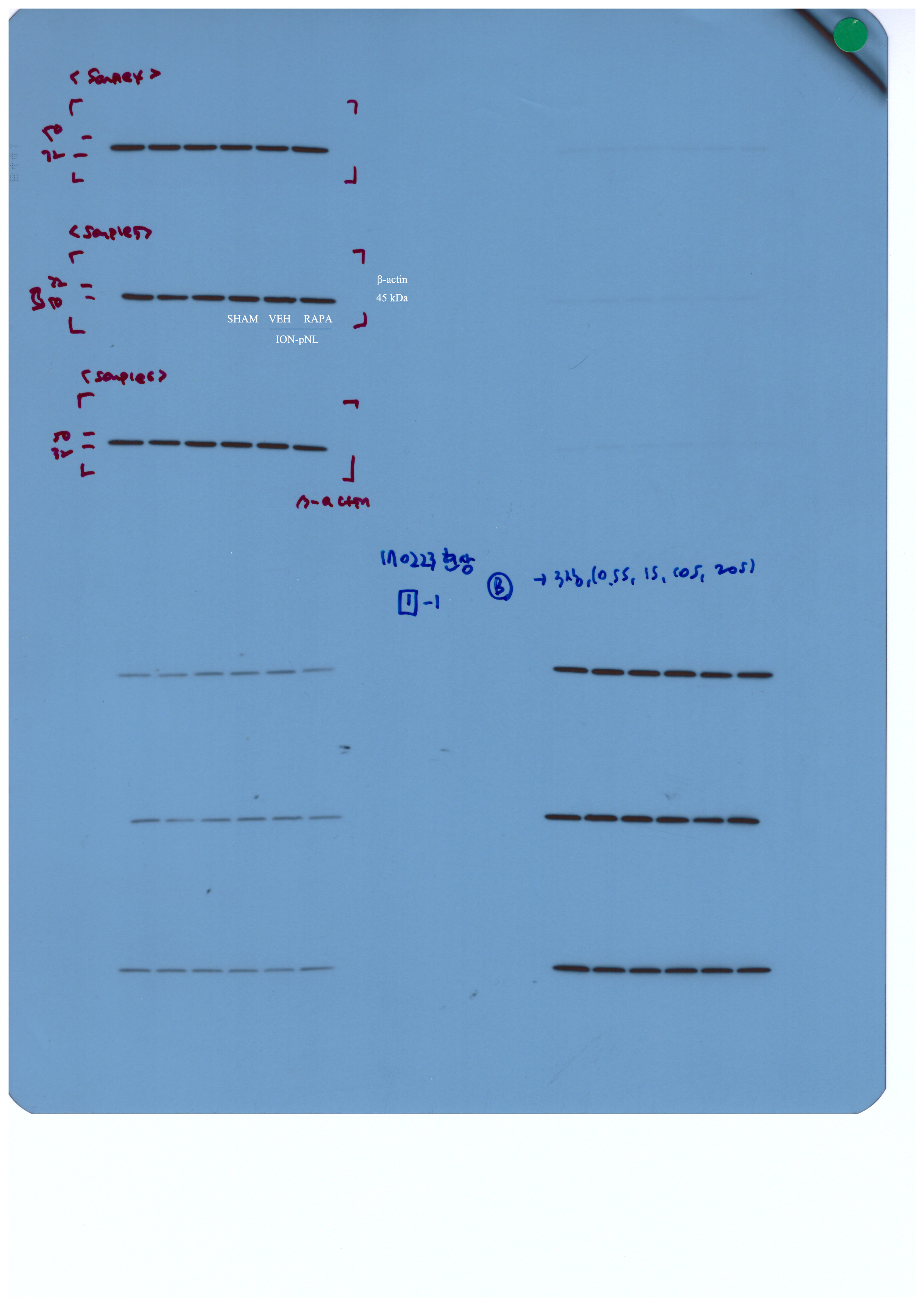

Supplement: Supplementary file 4 [file Data_Sheet_4_v1.ZIP › Figure 4/Figure 4A,B_actin of p-mTOR,mTOR.jpg]

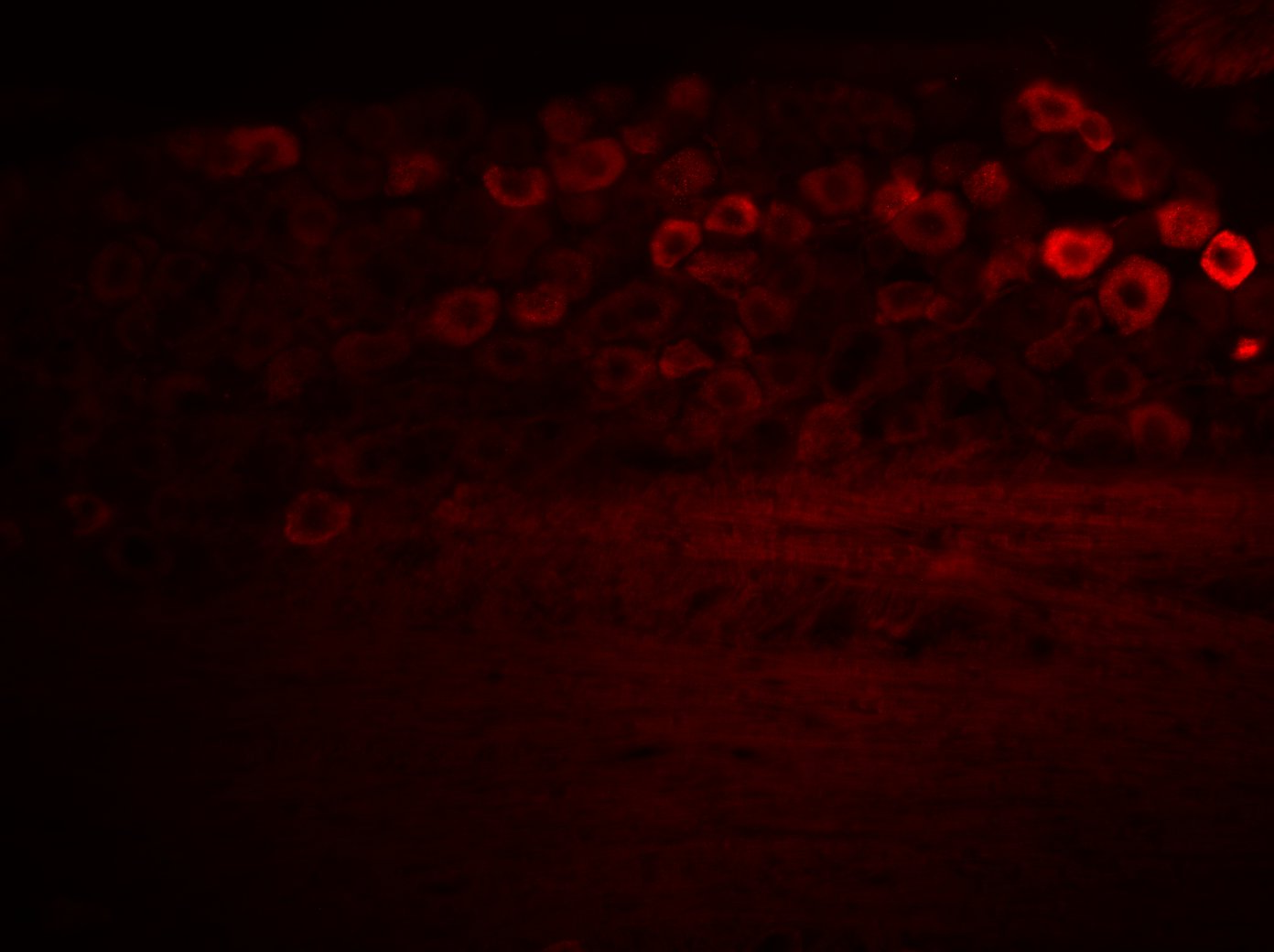

Supplement: Supplementary file 4 [file Data_Sheet_4_v1.ZIP › Figure 4/RAPA_scal bar.tif]

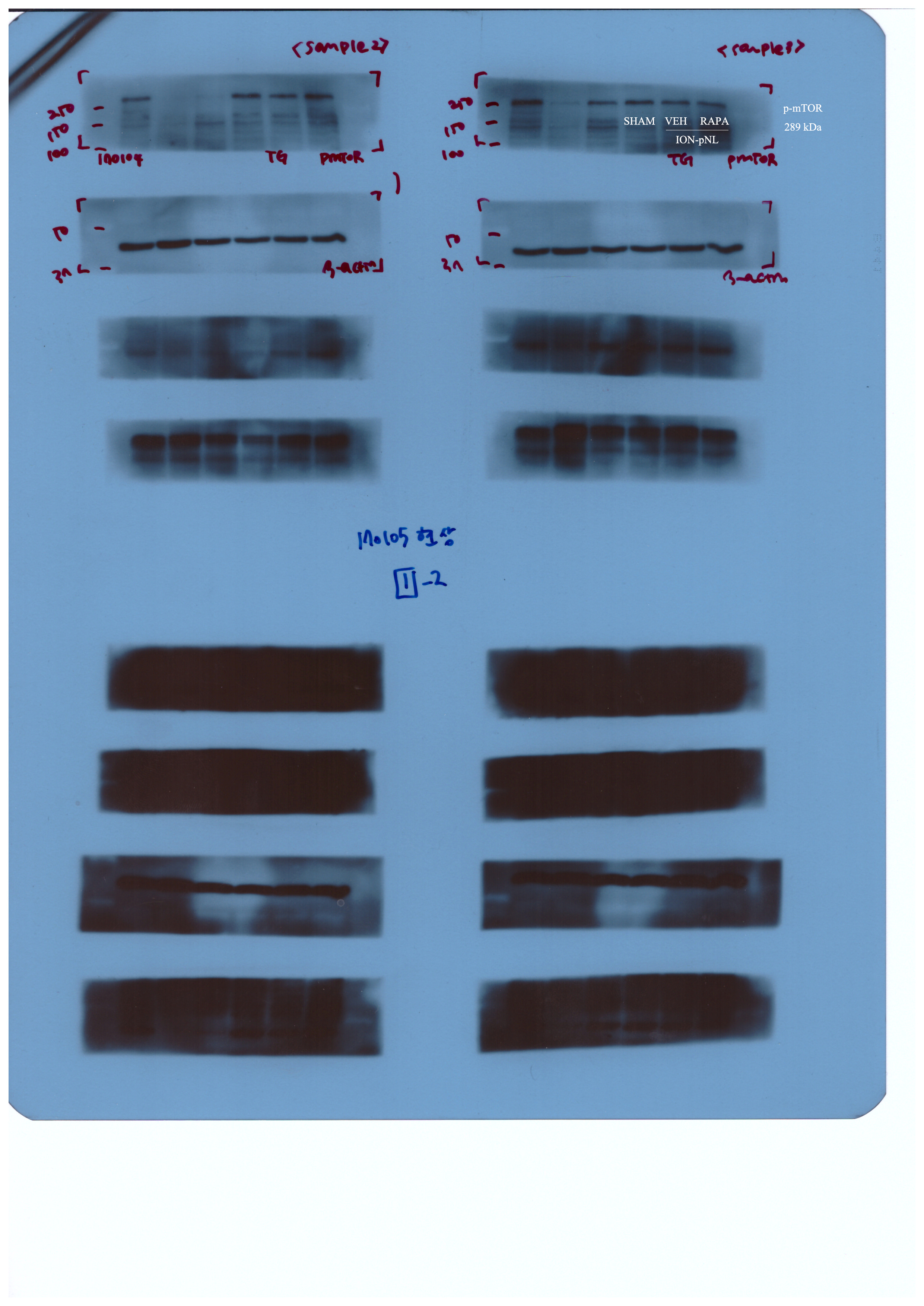

Supplement: Supplementary file 4 [file Data_Sheet_4_v1.ZIP › Figure 4/Figure 4A,B_p-mTOR.jpg]

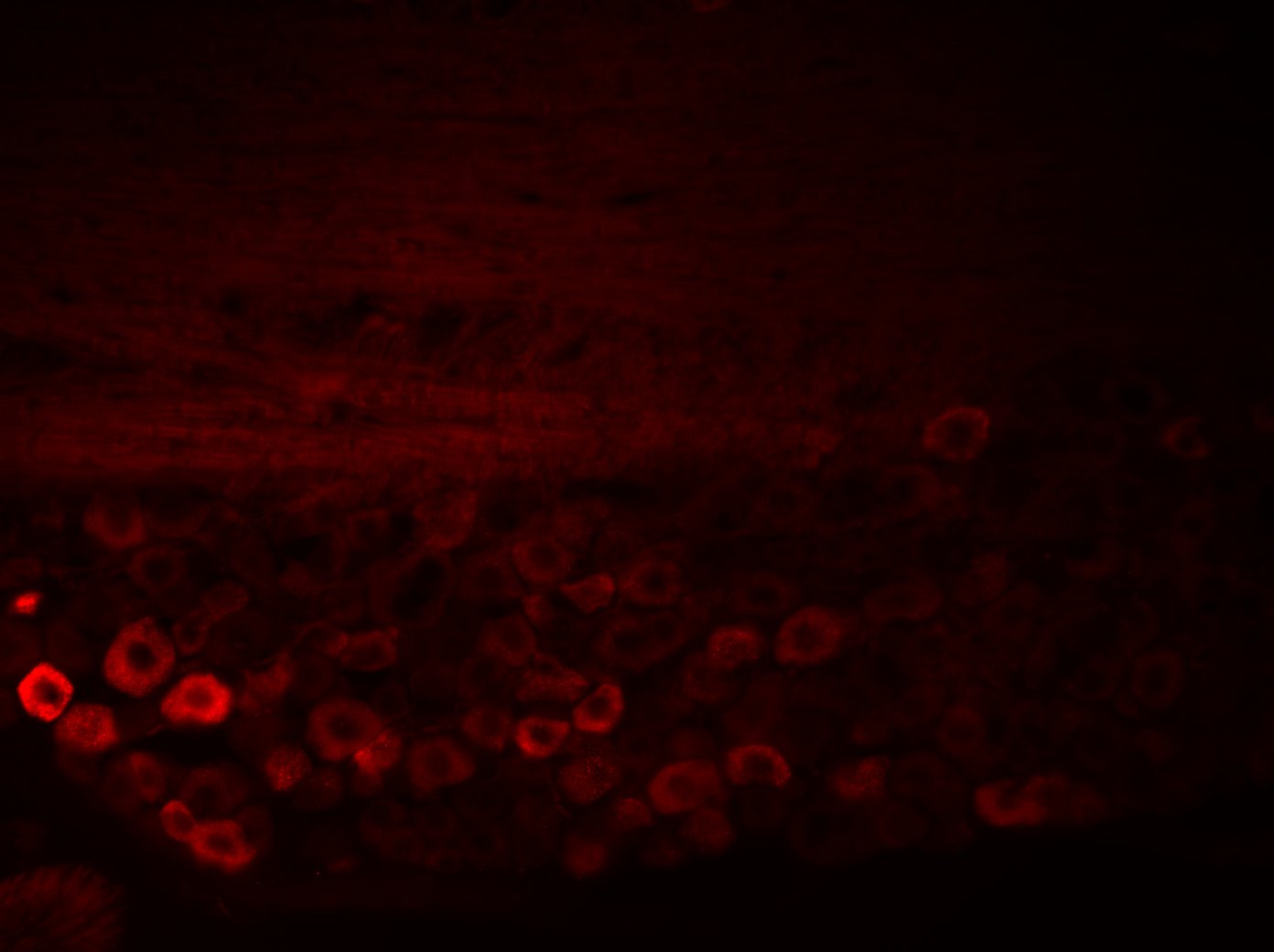

Supplement: Supplementary file 4 [file Data_Sheet_4_v1.ZIP › Figure 4/Figure 4E_RAPA.jpg]

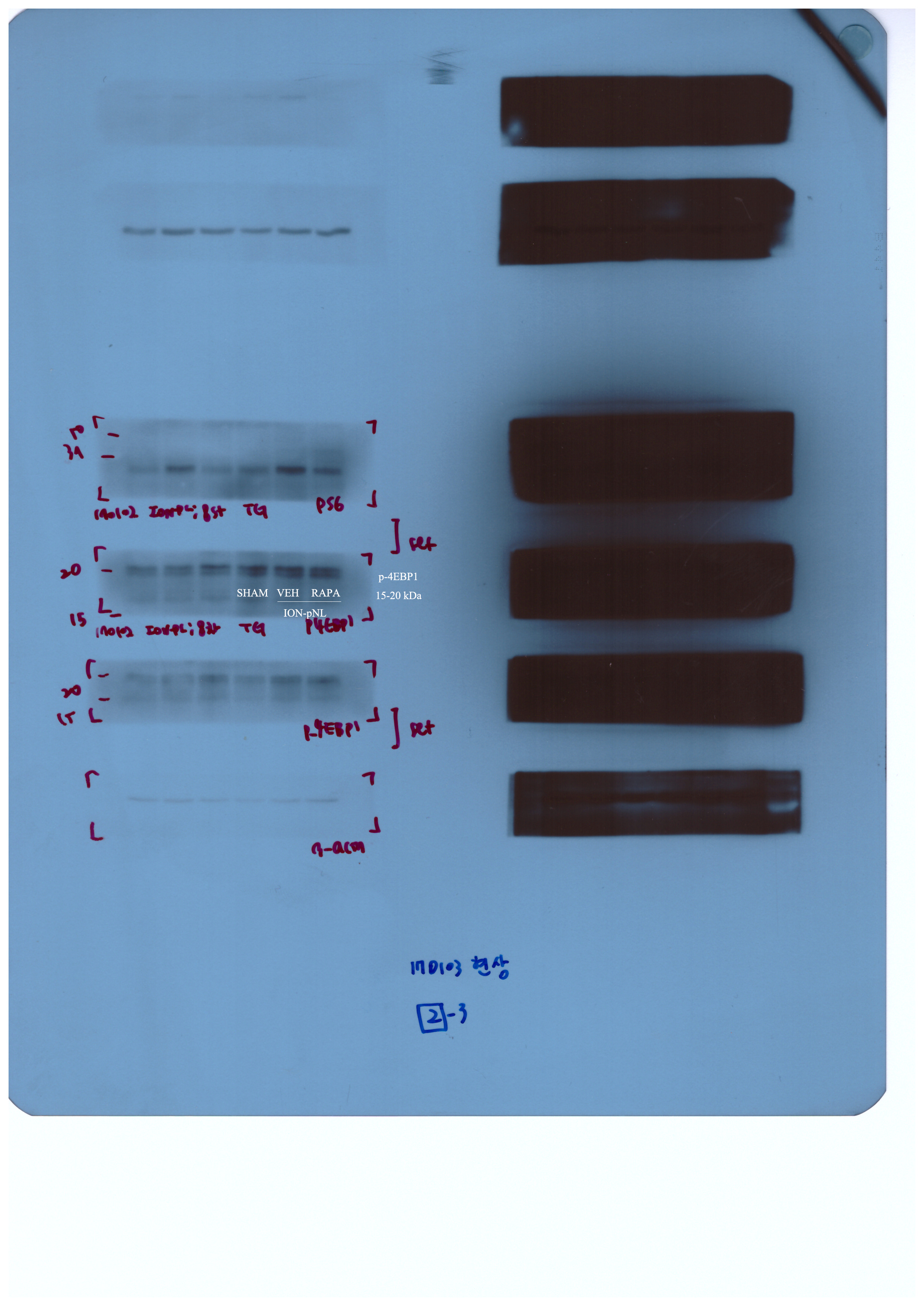

Supplement: Supplementary file 4 [file Data_Sheet_4_v1.ZIP › Figure 4/Figure 4A,D_p-4EBP1.jpg]

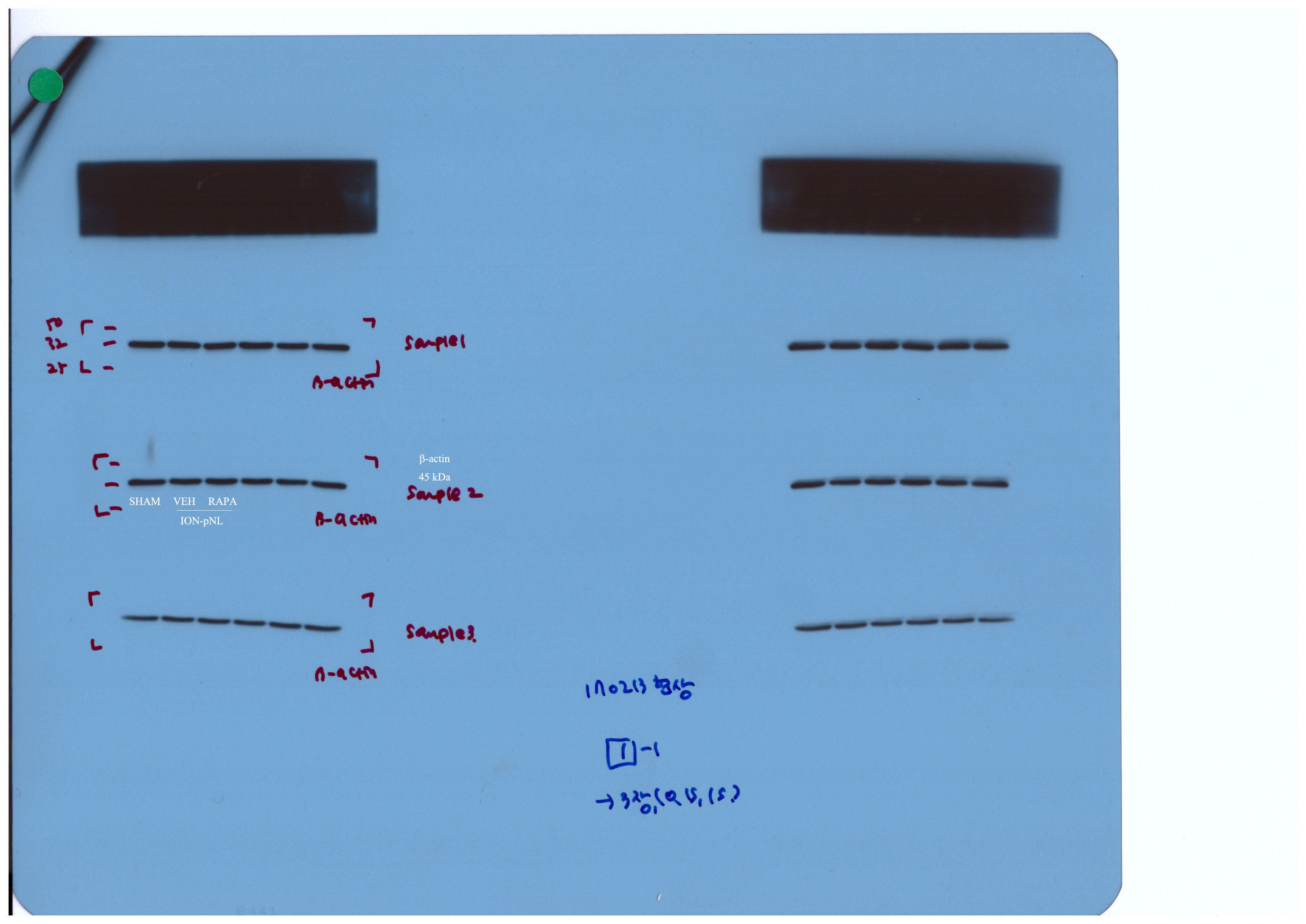

Supplement: Supplementary file 4 [file Data_Sheet_4_v1.ZIP › Figure 4/Figure 4A,D_actin of p-4EBP1, 4EBP1.jpg]

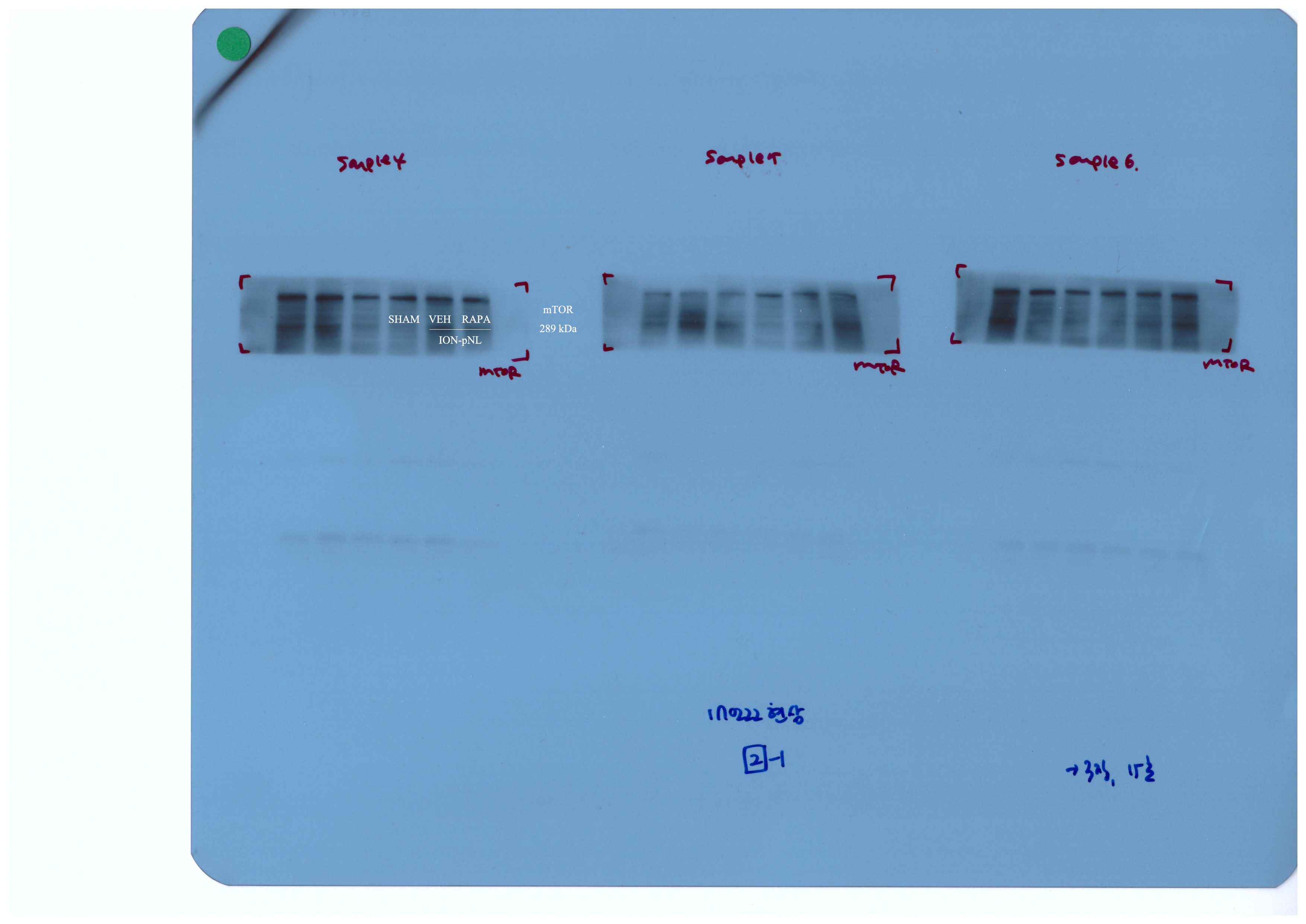

Supplement: Supplementary file 4 [file Data_Sheet_4_v1.ZIP › Figure 4/Figure 4A,B_mTOR.jpg]

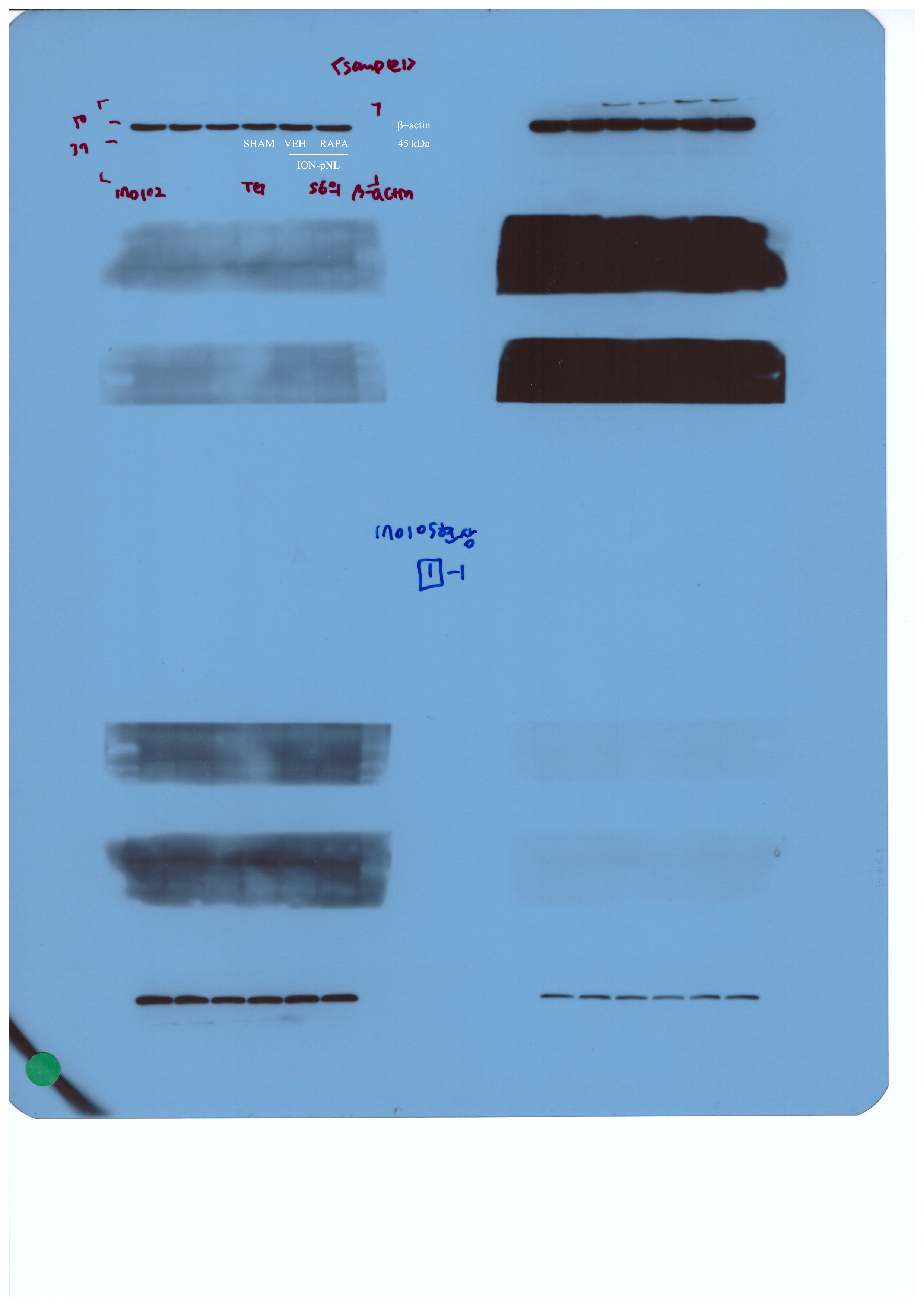

Supplement: Supplementary file 4 [file Data_Sheet_4_v1.ZIP › Figure 4/Figure 4A,B_actin of p-S6,S6.jpg]

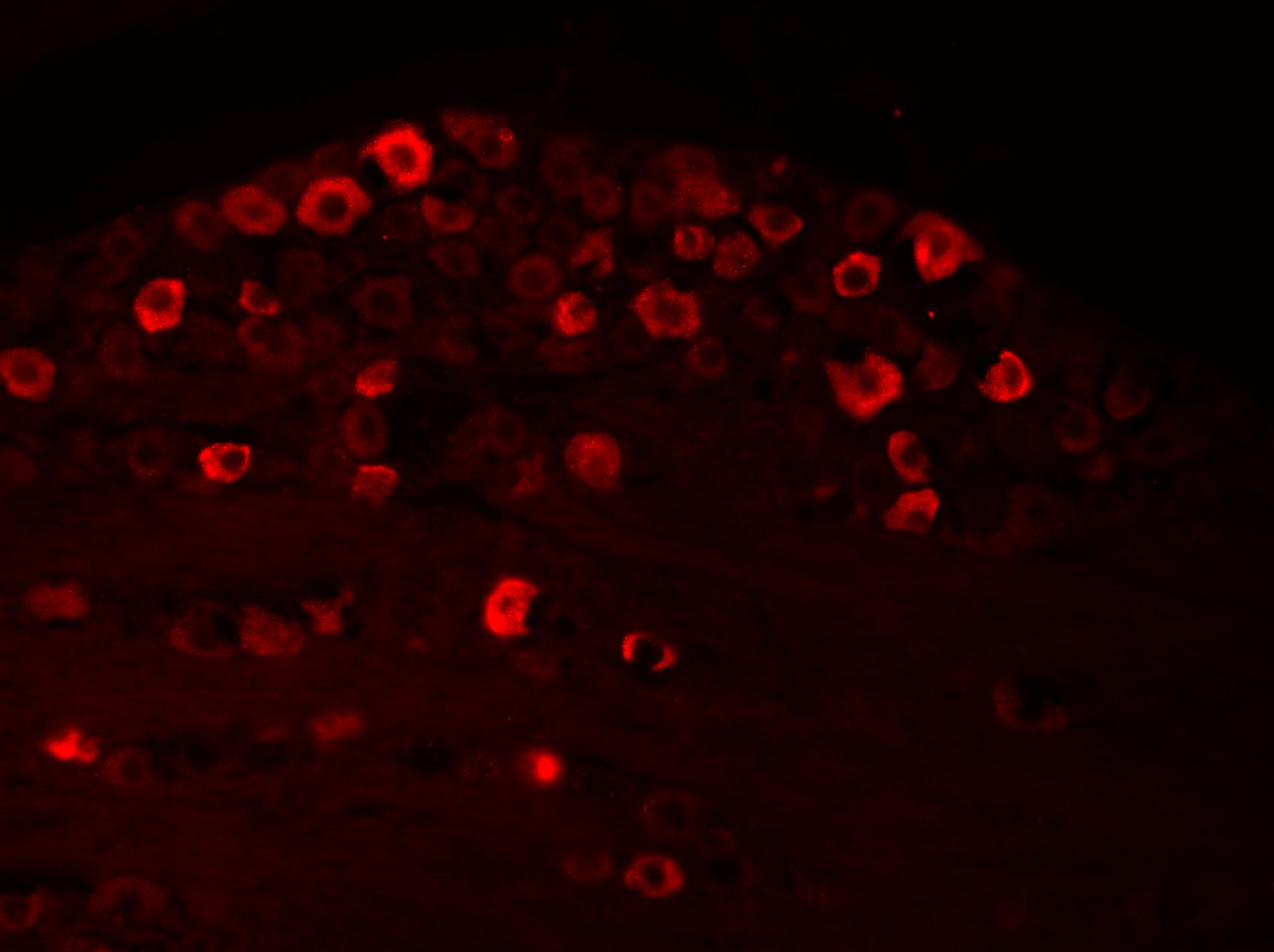

Supplement: Supplementary file 4 [file Data_Sheet_4_v1.ZIP › Figure 4/Figure 4E_VEH.jpg]

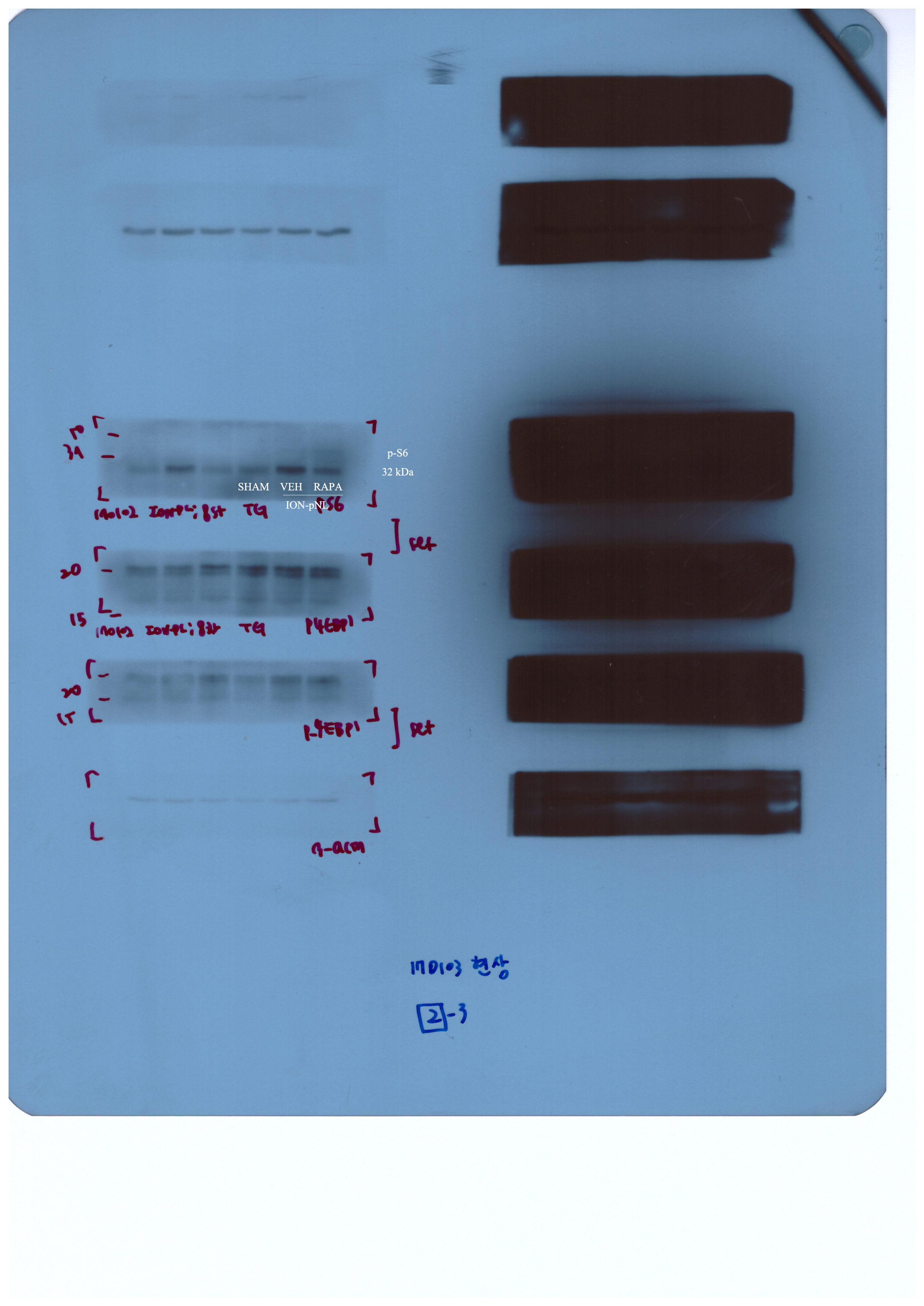

Supplement: Supplementary file 4 [file Data_Sheet_4_v1.ZIP › Figure 4/Figure 4A,B_p-S6.jpg]

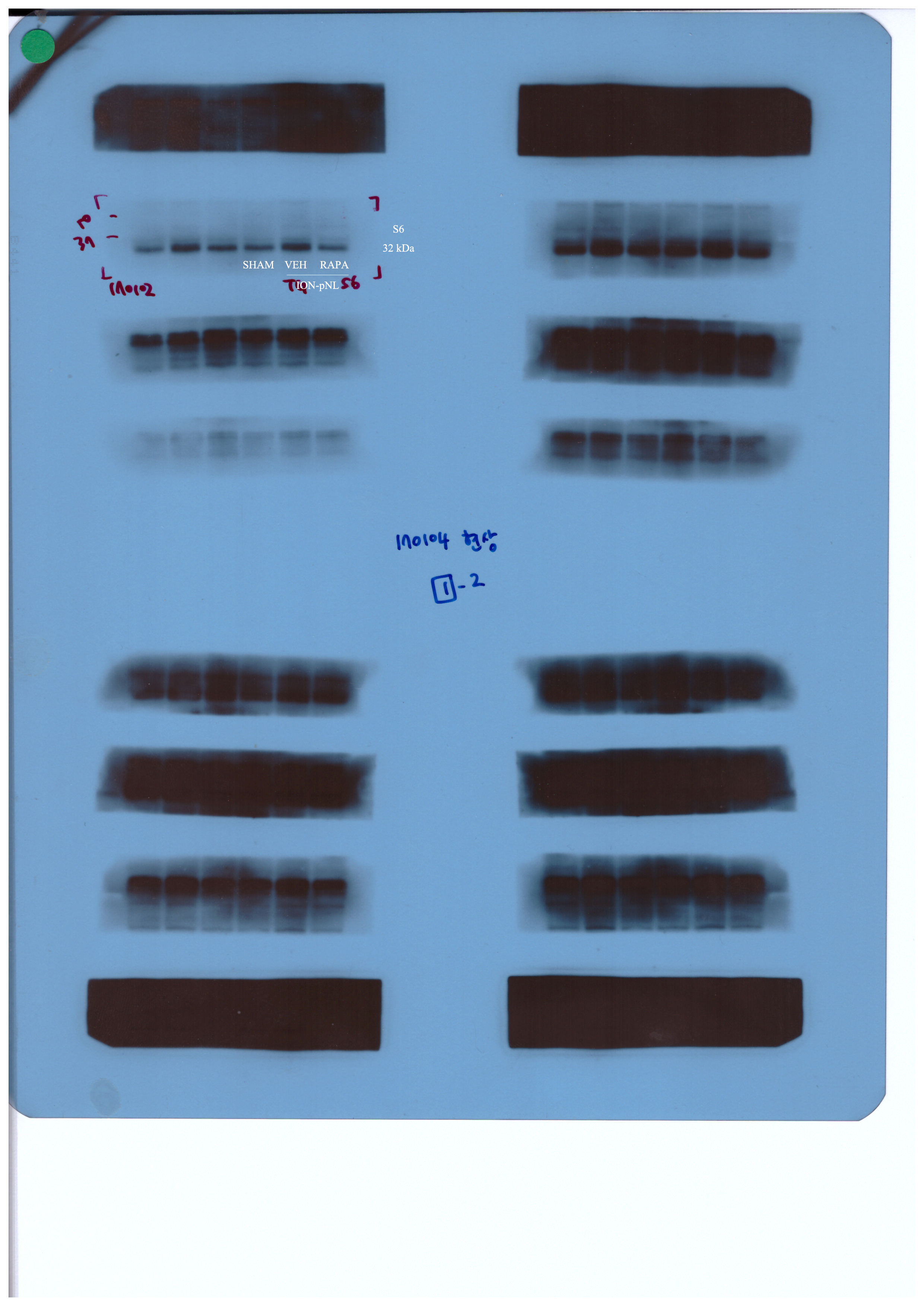

Supplement: Supplementary file 4 [file Data_Sheet_4_v1.ZIP › Figure 4/Figure 4A,B_S6.jpg]

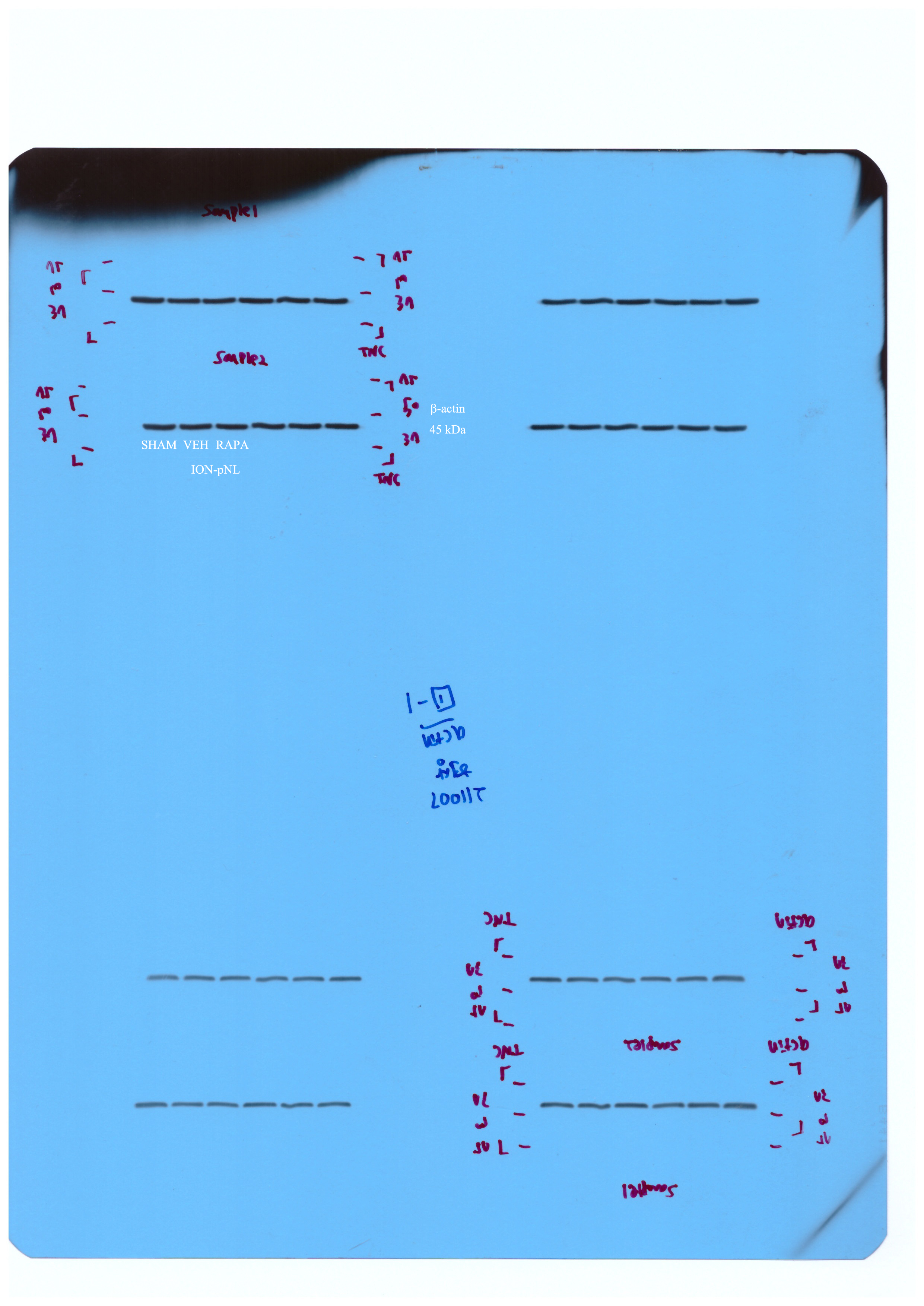

Supplement: Supplementary file 5 [file Data_Sheet_5_v1.ZIP › Figure 5/Figure 4A-C_actin of GFAP,Iba1.jpg]

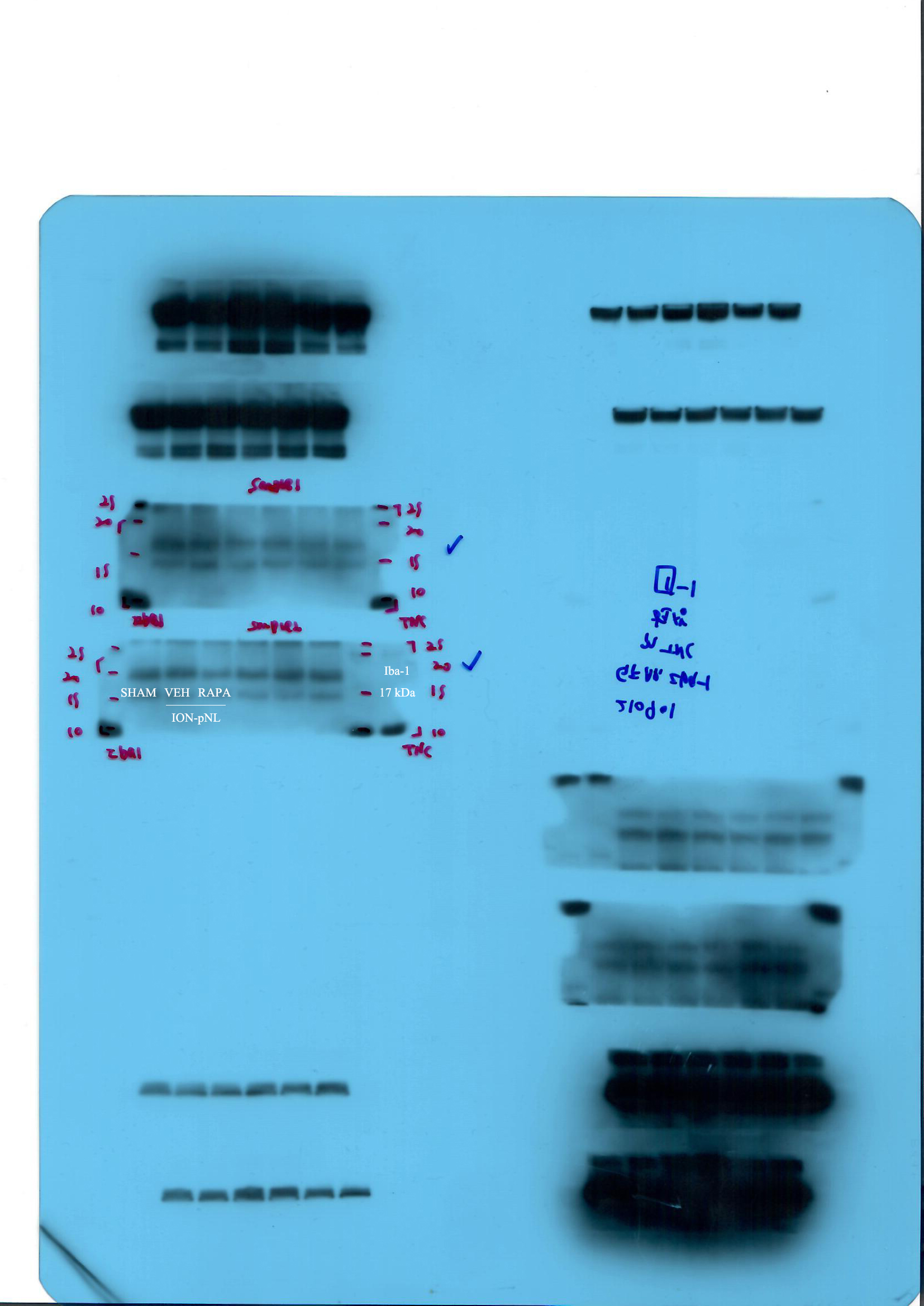

Supplement: Supplementary file 5 [file Data_Sheet_5_v1.ZIP › Figure 5/Figure 4A,C_Iba1.jpg]

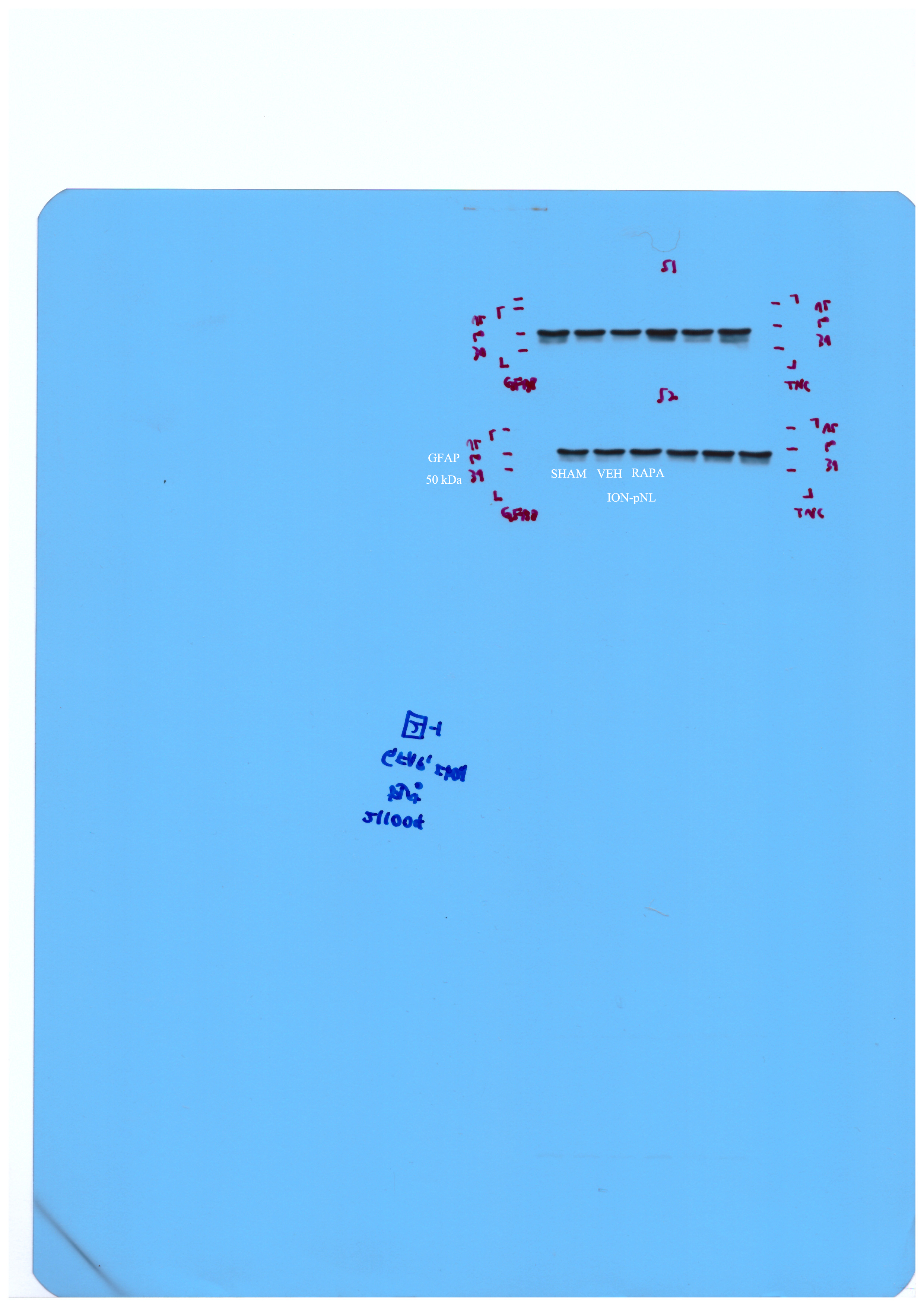

Supplement: Supplementary file 5 [file Data_Sheet_5_v1.ZIP › Figure 5/Figure 4A,B_GFAP.jpg]

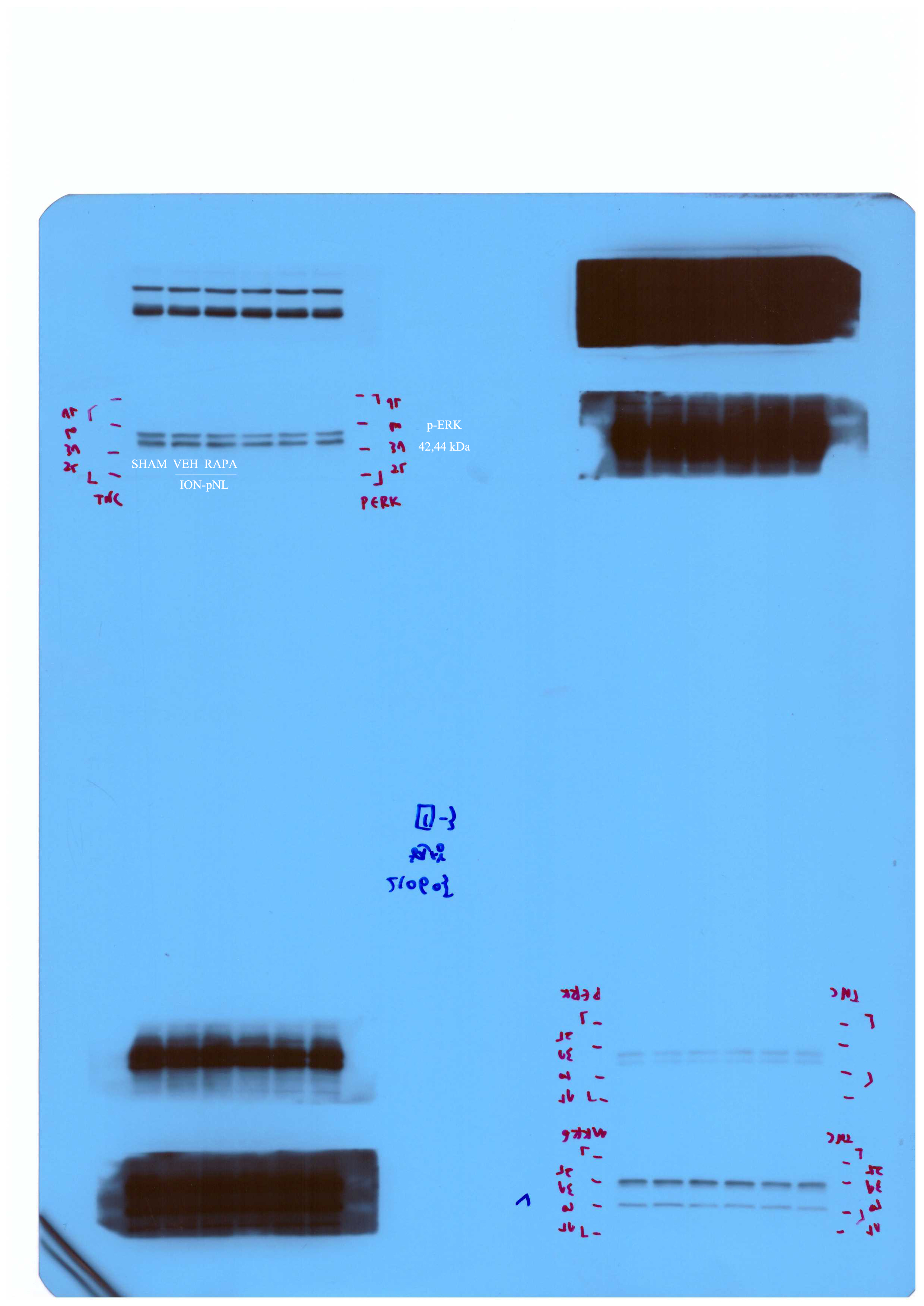

Supplement: Supplementary file 6 [file Data_Sheet_6_v1.ZIP › Figure 5/Figure 5A,C_p-ERK.jpg]

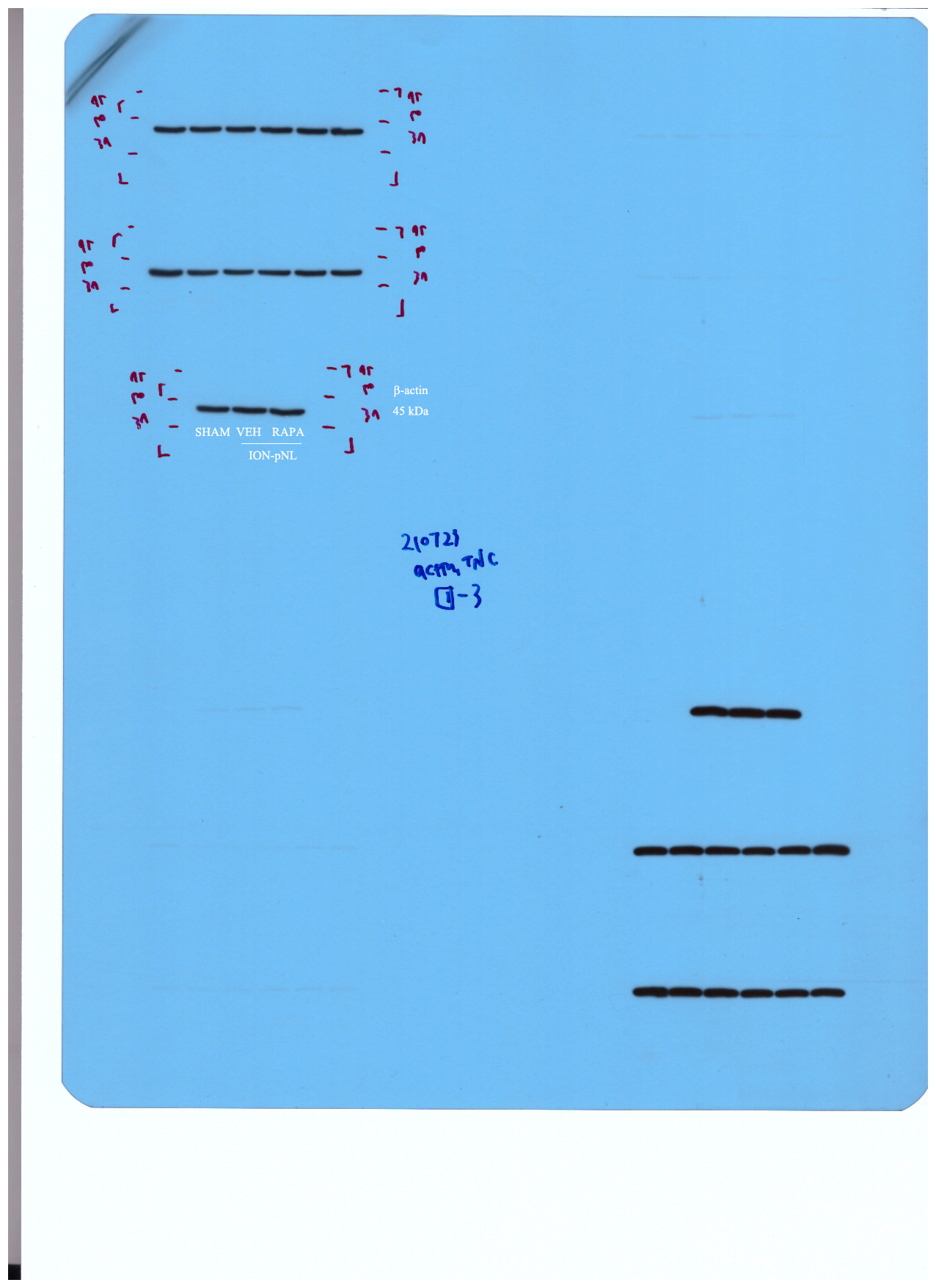

Supplement: Supplementary file 6 [file Data_Sheet_6_v1.ZIP › Figure 5/Figure 5A,D_actin of p38,p-p38.jpg]

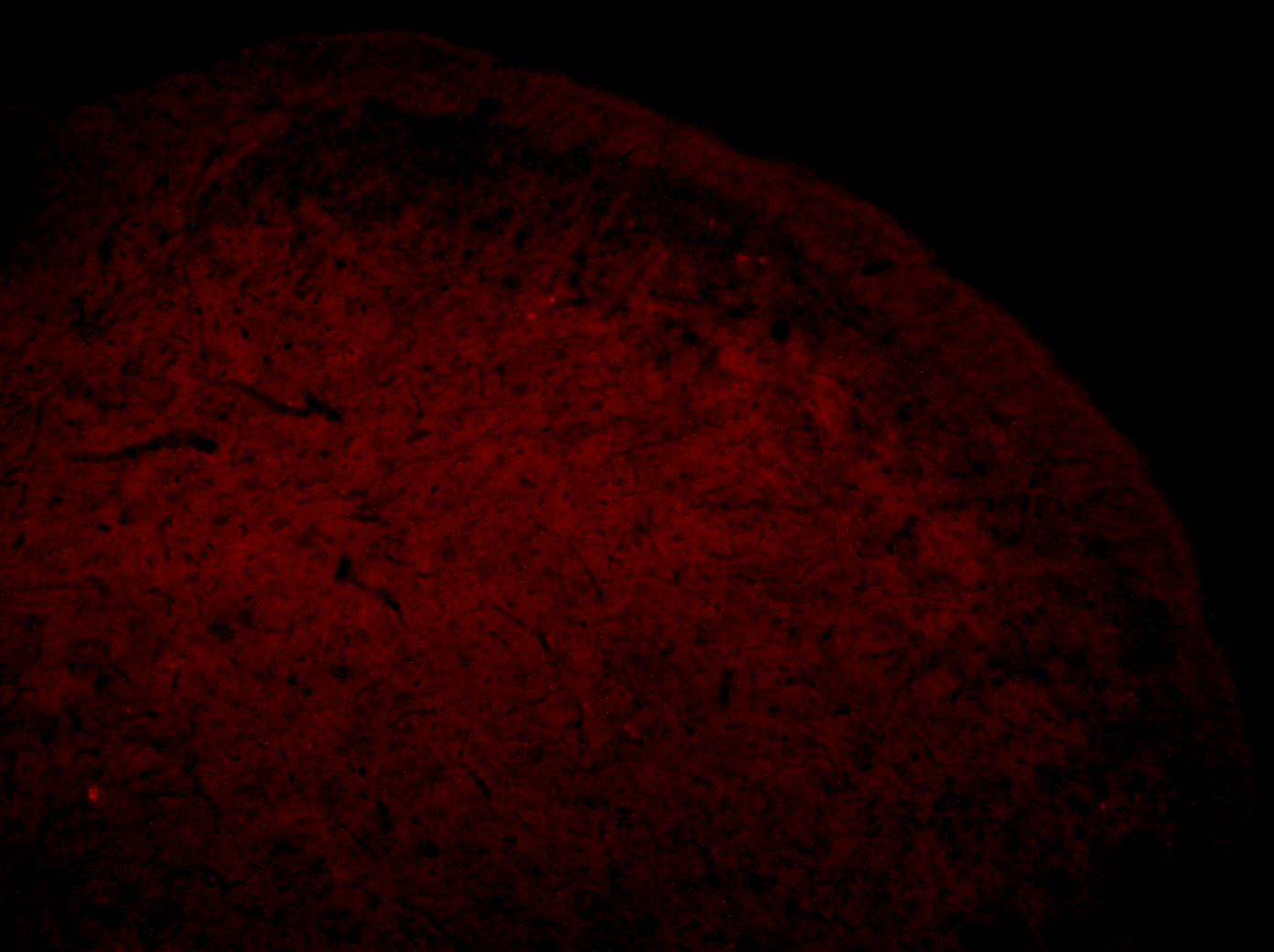

Supplement: Supplementary file 6 [file Data_Sheet_6_v1.ZIP › Figure 5/Figure 5E_SHAM.tif]

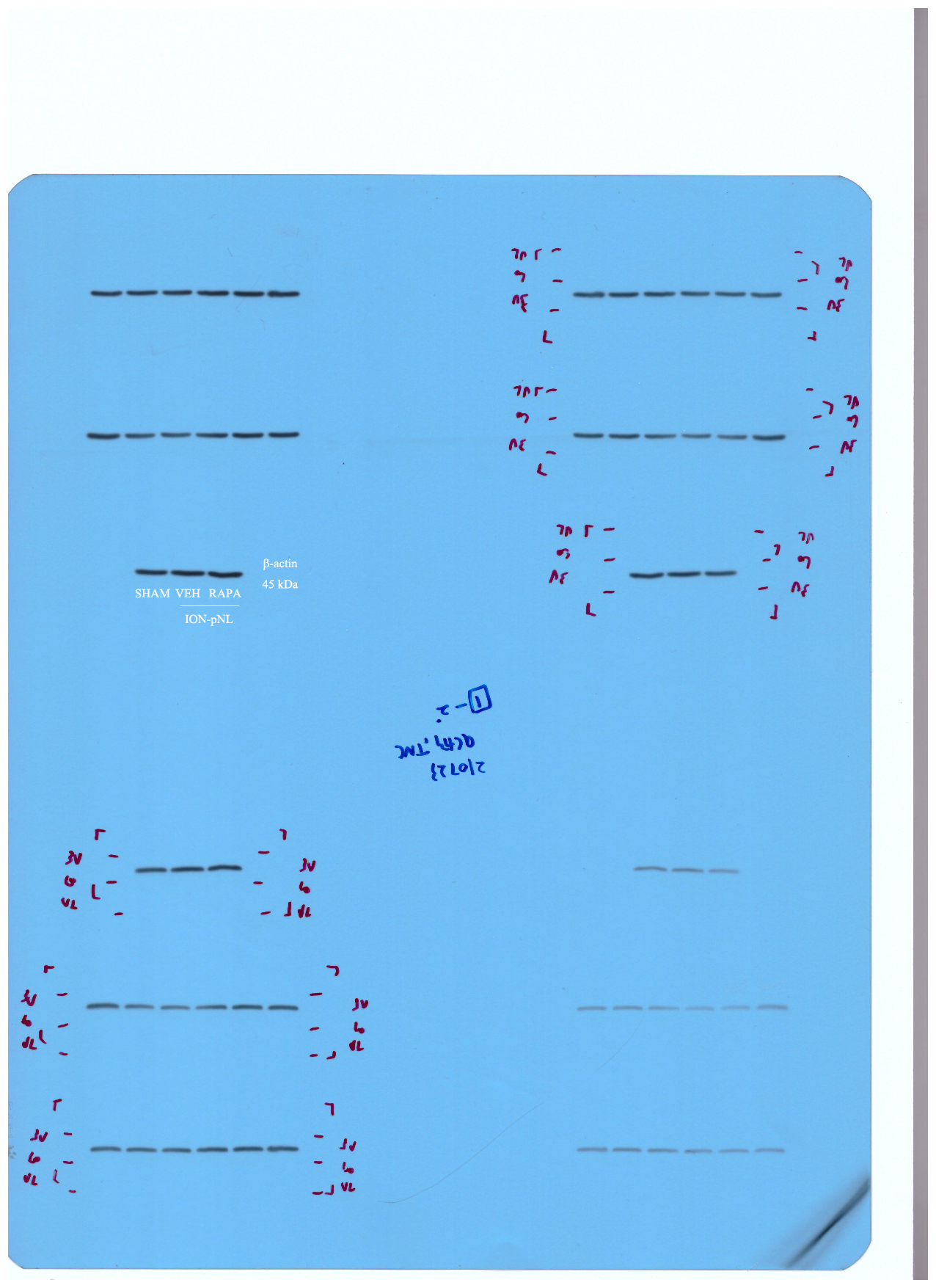

Supplement: Supplementary file 6 [file Data_Sheet_6_v1.ZIP › Figure 5/Figure 5A,C_actin of ERK,p-ERK.jpg]

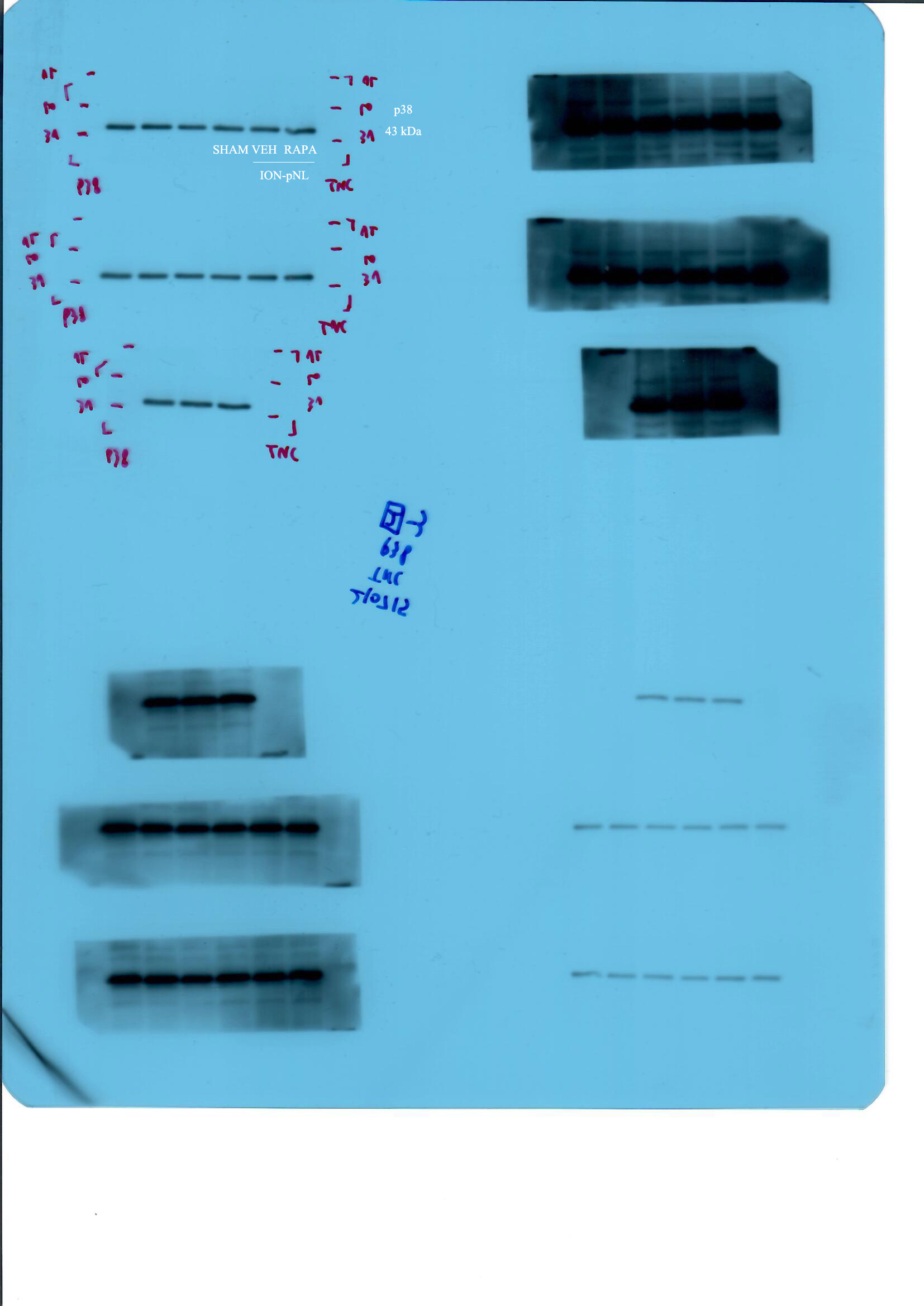

Supplement: Supplementary file 6 [file Data_Sheet_6_v1.ZIP › Figure 5/Figure 5A,D_p38.jpg]

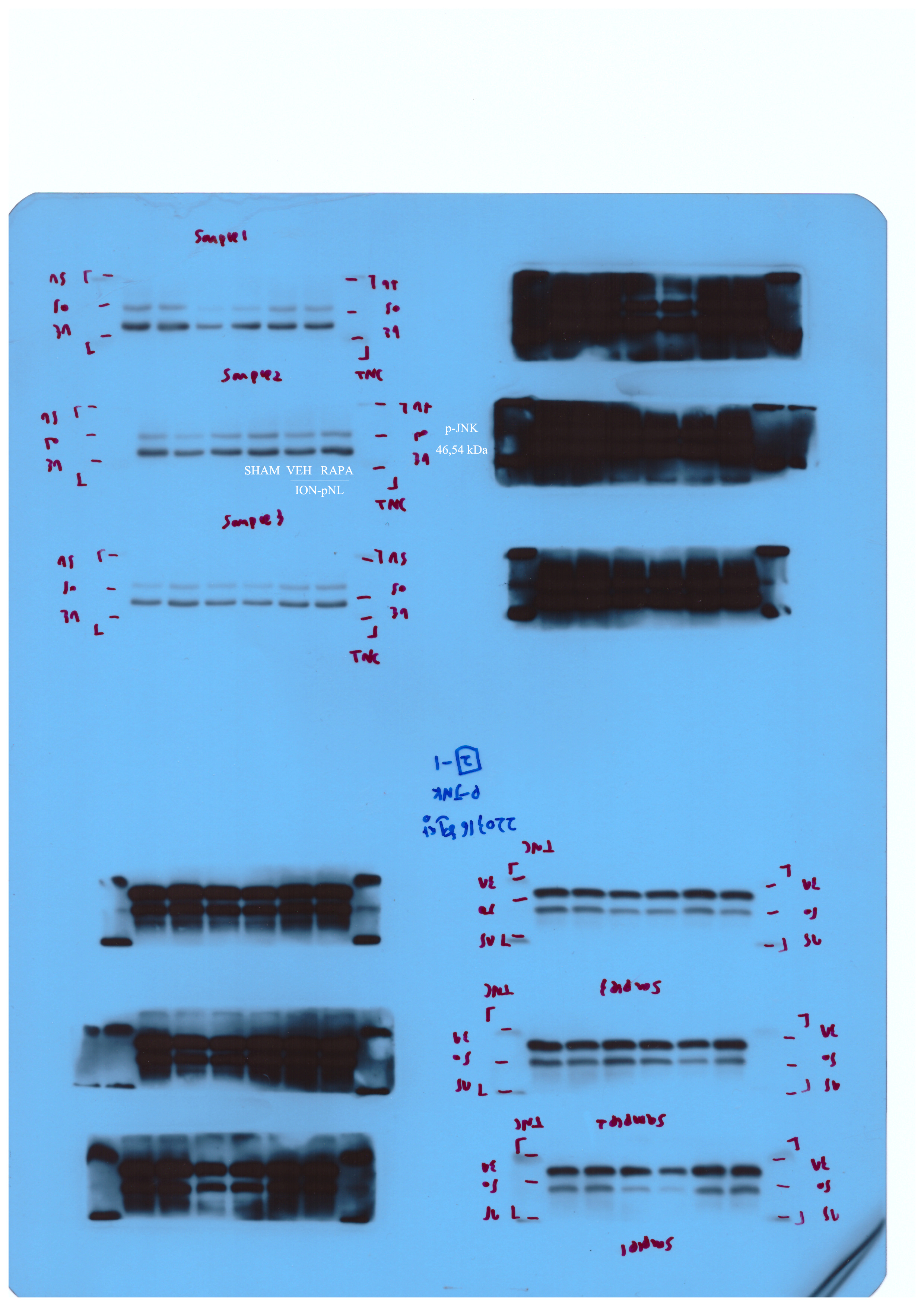

Supplement: Supplementary file 6 [file Data_Sheet_6_v1.ZIP › Figure 5/Figure 5A,B_p-JNK.jpg]

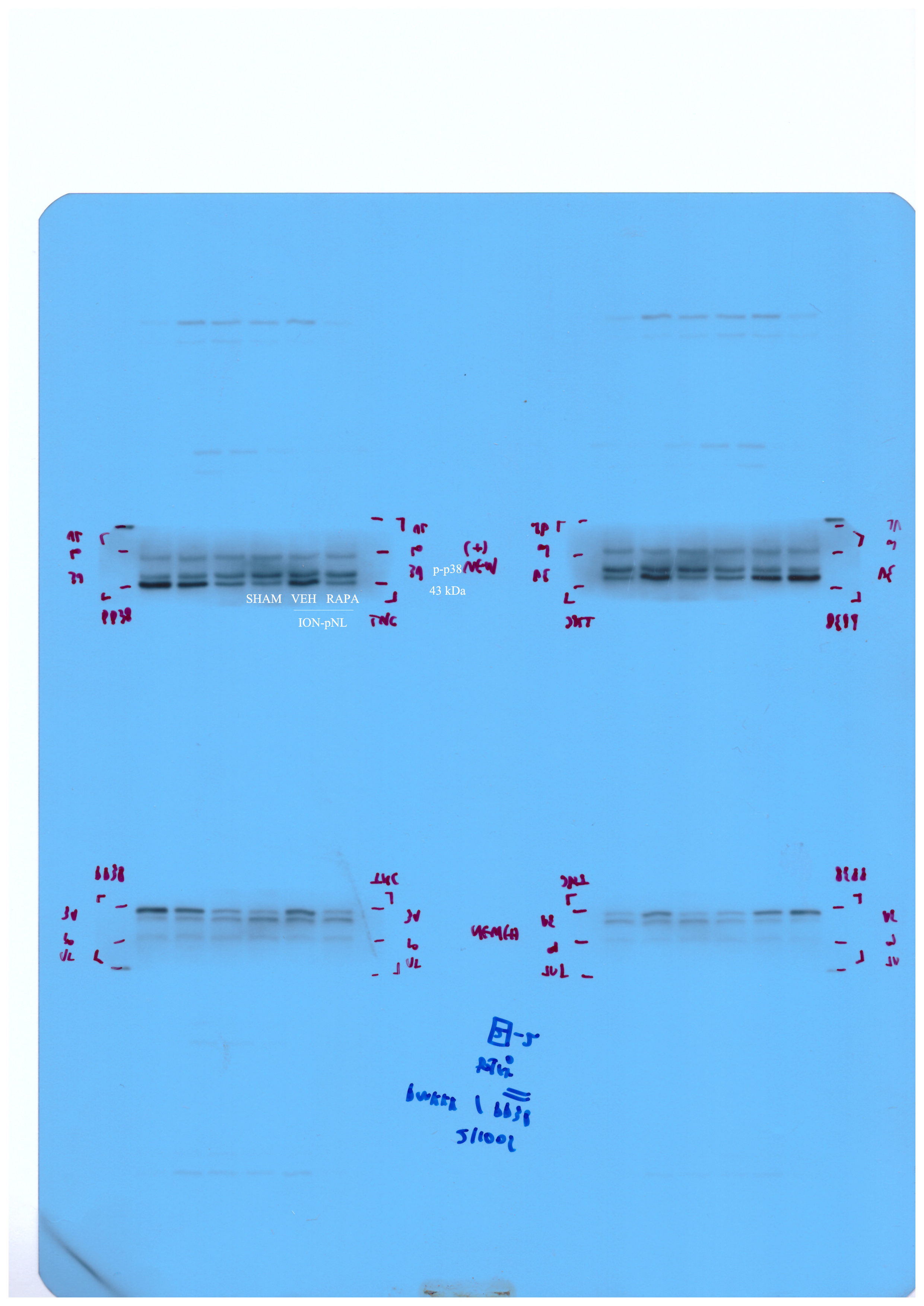

Supplement: Supplementary file 6 [file Data_Sheet_6_v1.ZIP › Figure 5/Figure 5A,D_p-p38.jpg]

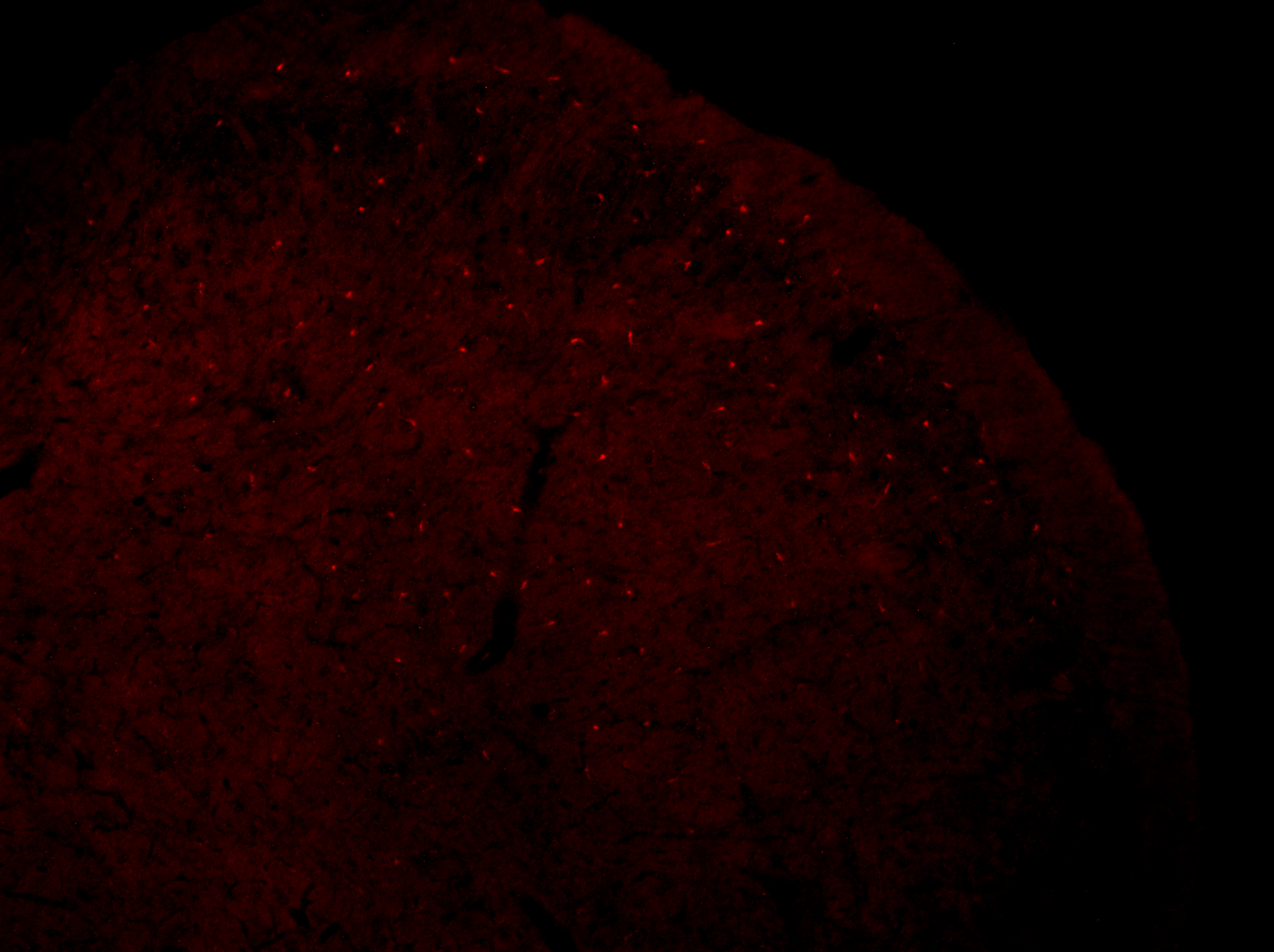

Supplement: Supplementary file 6 [file Data_Sheet_6_v1.ZIP › Figure 5/Figure 5E_RAPA.tif]

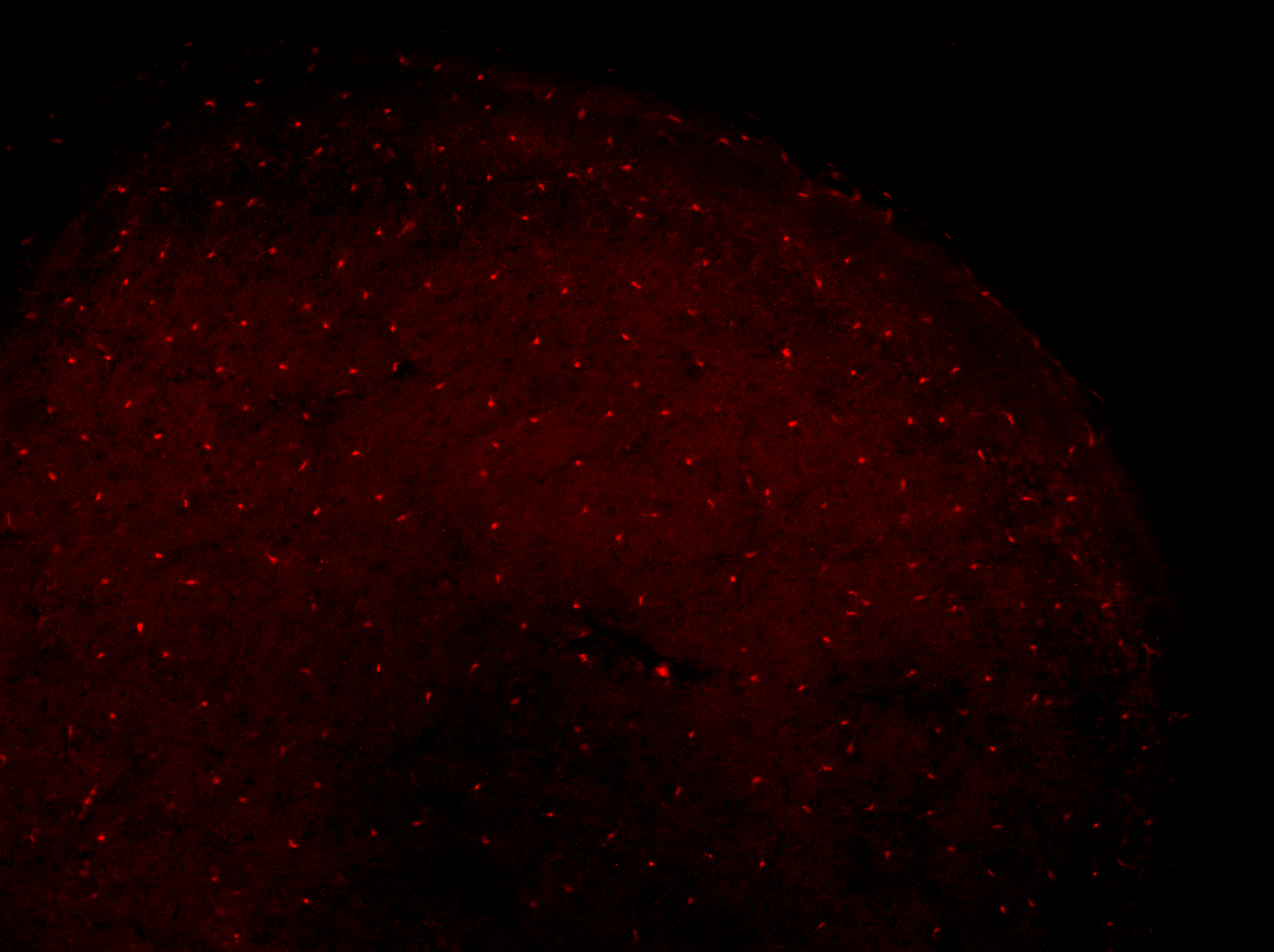

Supplement: Supplementary file 6 [file Data_Sheet_6_v1.ZIP › Figure 5/Figure 5E_VEH.tif]

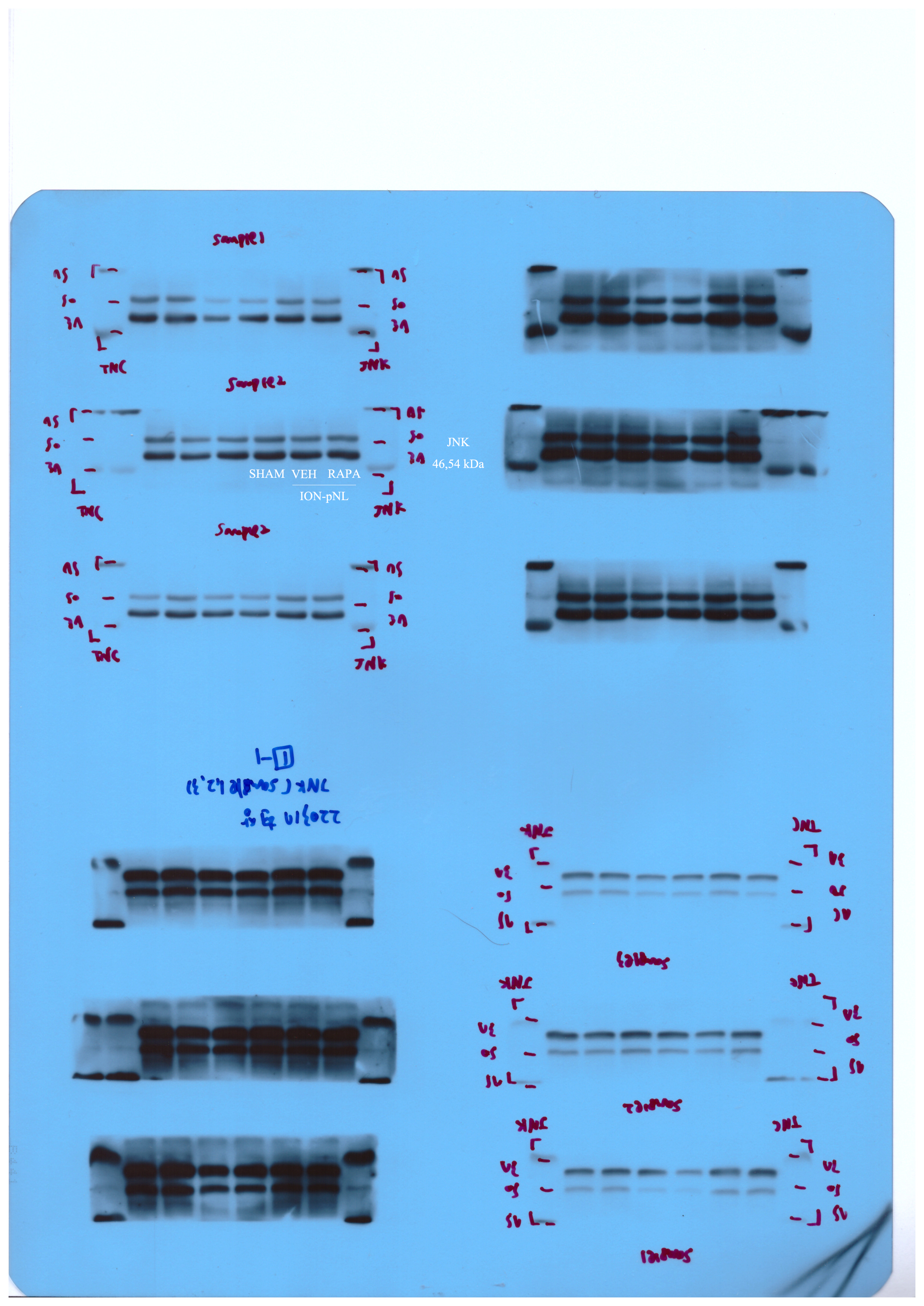

Supplement: Supplementary file 6 [file Data_Sheet_6_v1.ZIP › Figure 5/Figure 5A,B_JNK.jpg]

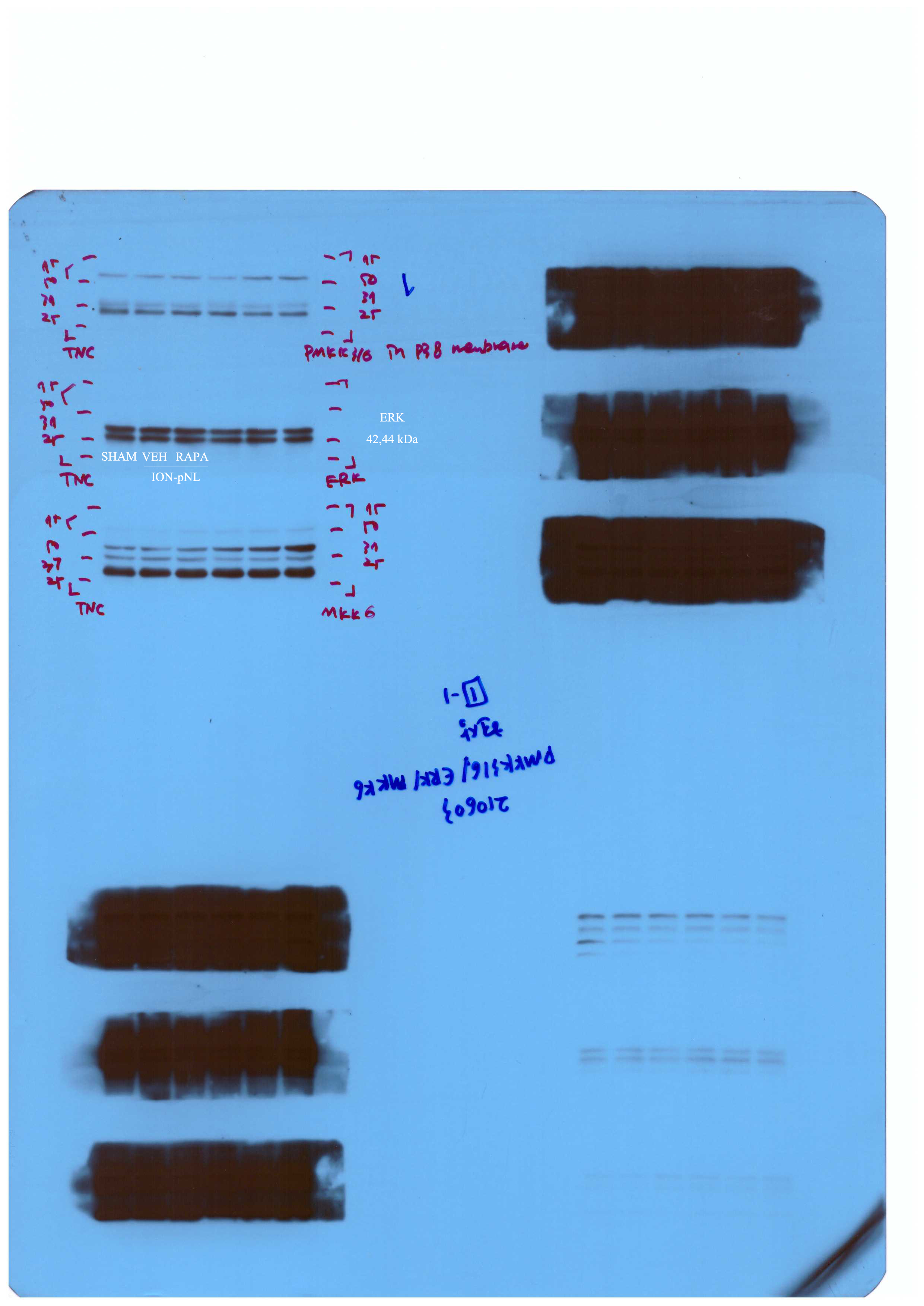

Supplement: Supplementary file 6 [file Data_Sheet_6_v1.ZIP › Figure 5/Figure 5A,C_ERK.jpg]

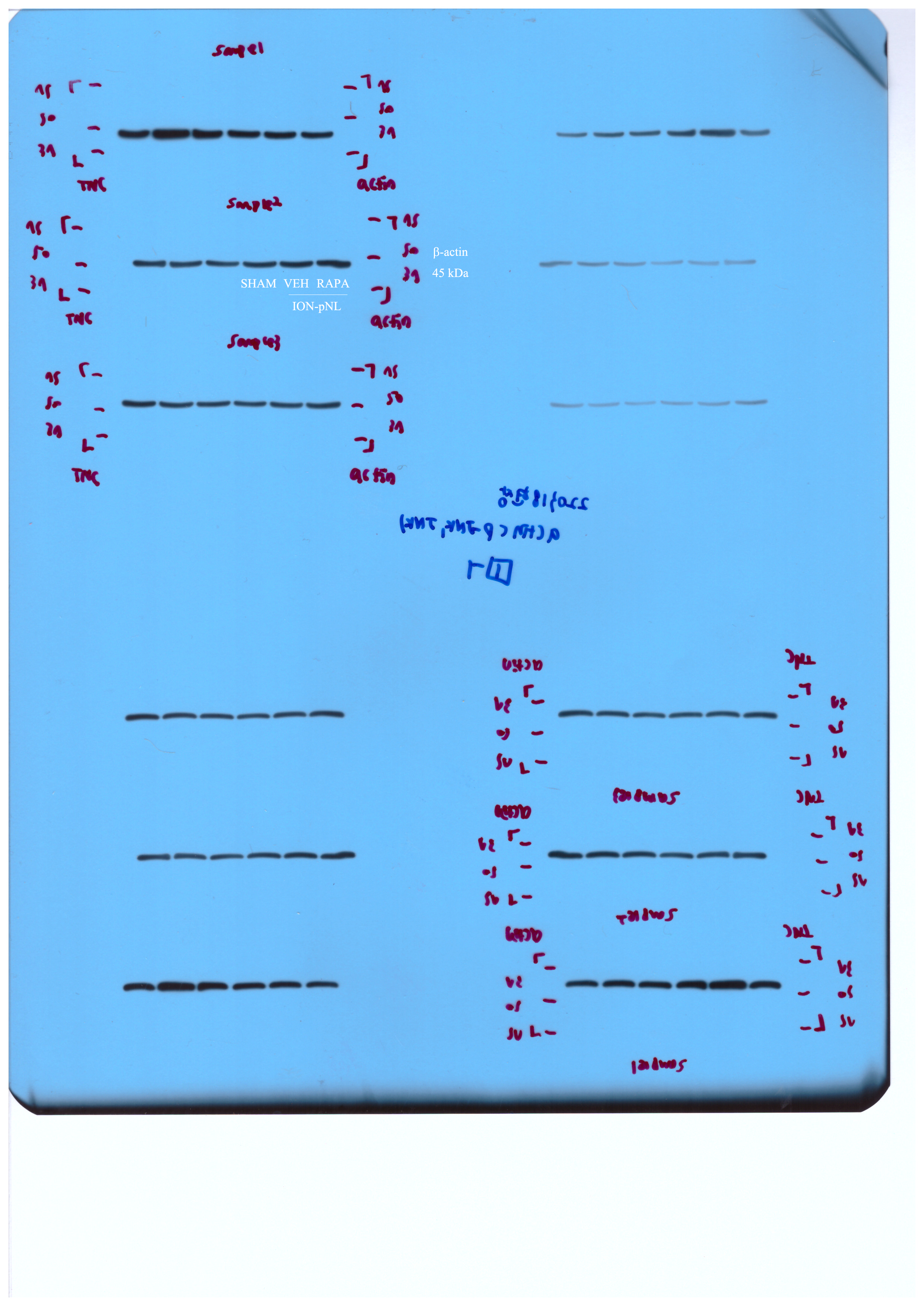

Supplement: Supplementary file 6 [file Data_Sheet_6_v1.ZIP › Figure 5/Figure 5A,B_actin of JNK.jpg]

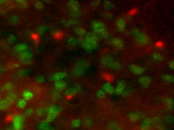

Supplement: Supplementary file 7 [file Data_Sheet_7_v1.ZIP › Figure 7/Figure 6F_overlay_enlarged data.tif]

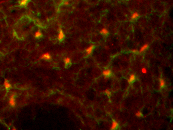

Supplement: Supplementary file 7 [file Data_Sheet_7_v1.ZIP › Figure 7/Figure 6I_overlay_enlarged data.tif]

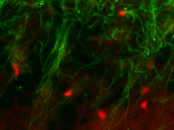

Supplement: Supplementary file 7 [file Data_Sheet_7_v1.ZIP › Figure 7/Figure 6C_overlay_enlarged data.tif]

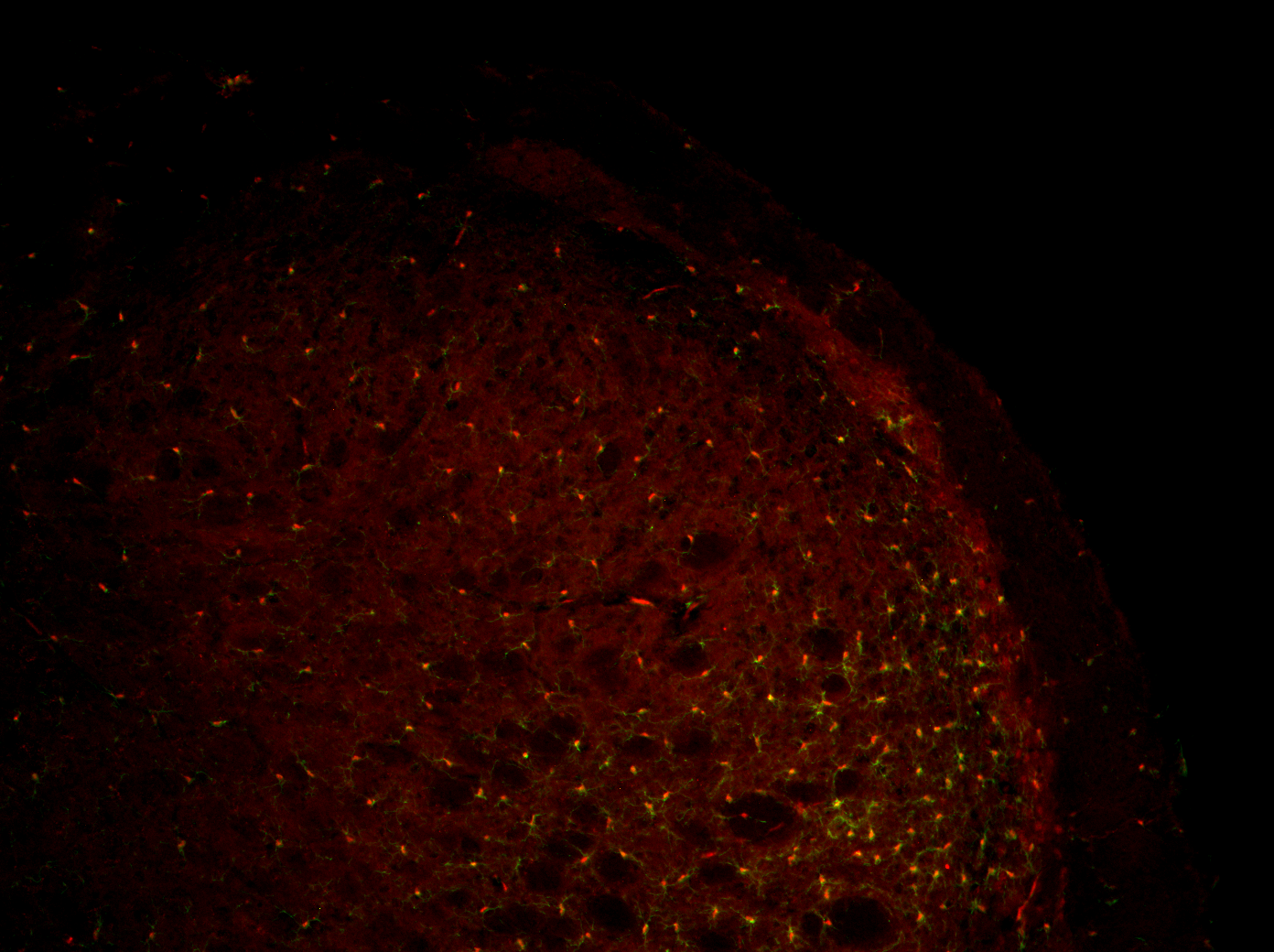

Supplement: Supplementary file 7 [file Data_Sheet_7_v1.ZIP › Figure 7/Figure 6I_overlay.tif]

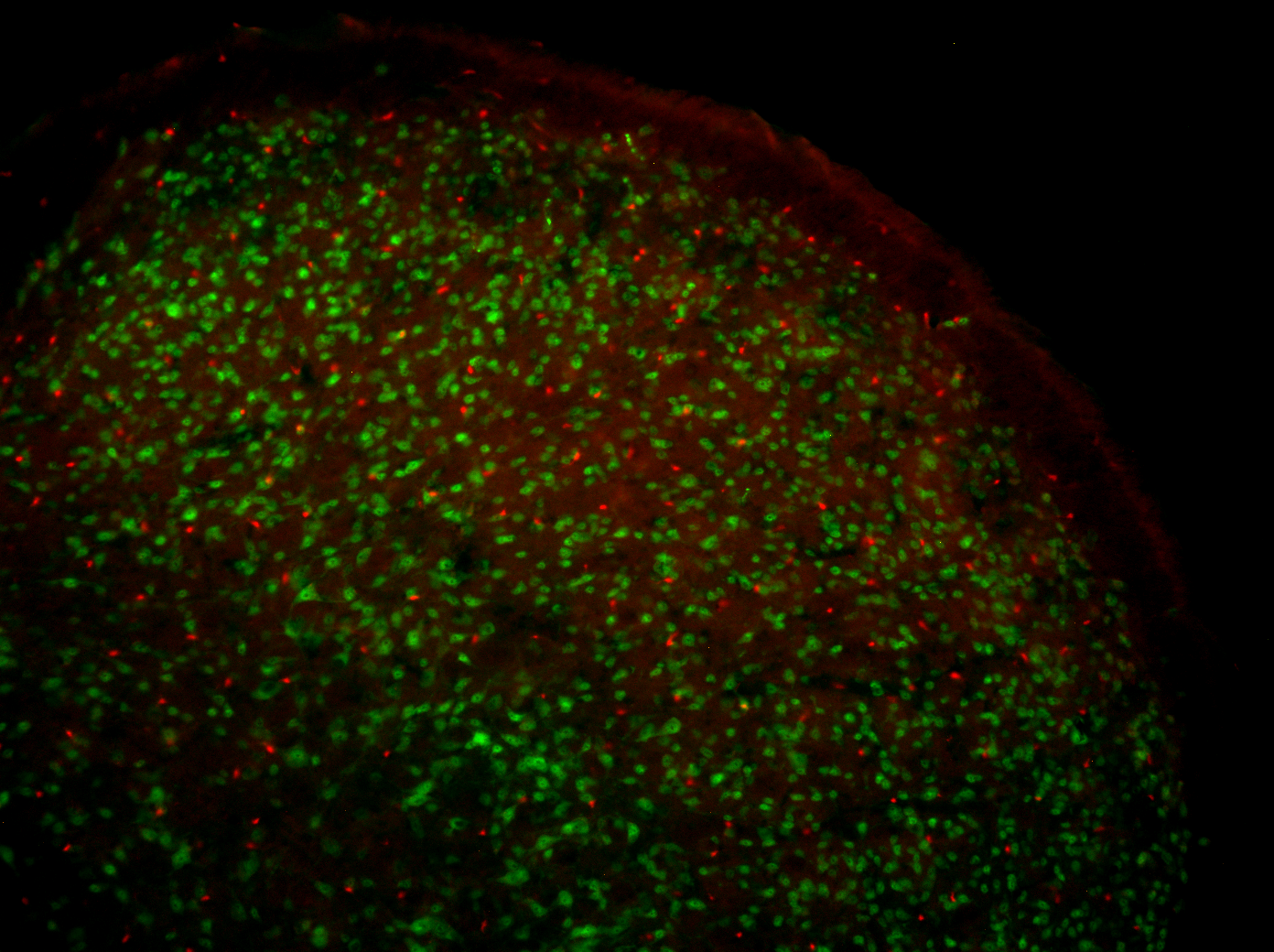

Supplement: Supplementary file 7 [file Data_Sheet_7_v1.ZIP › Figure 7/Figure 6F_overlay.tif]

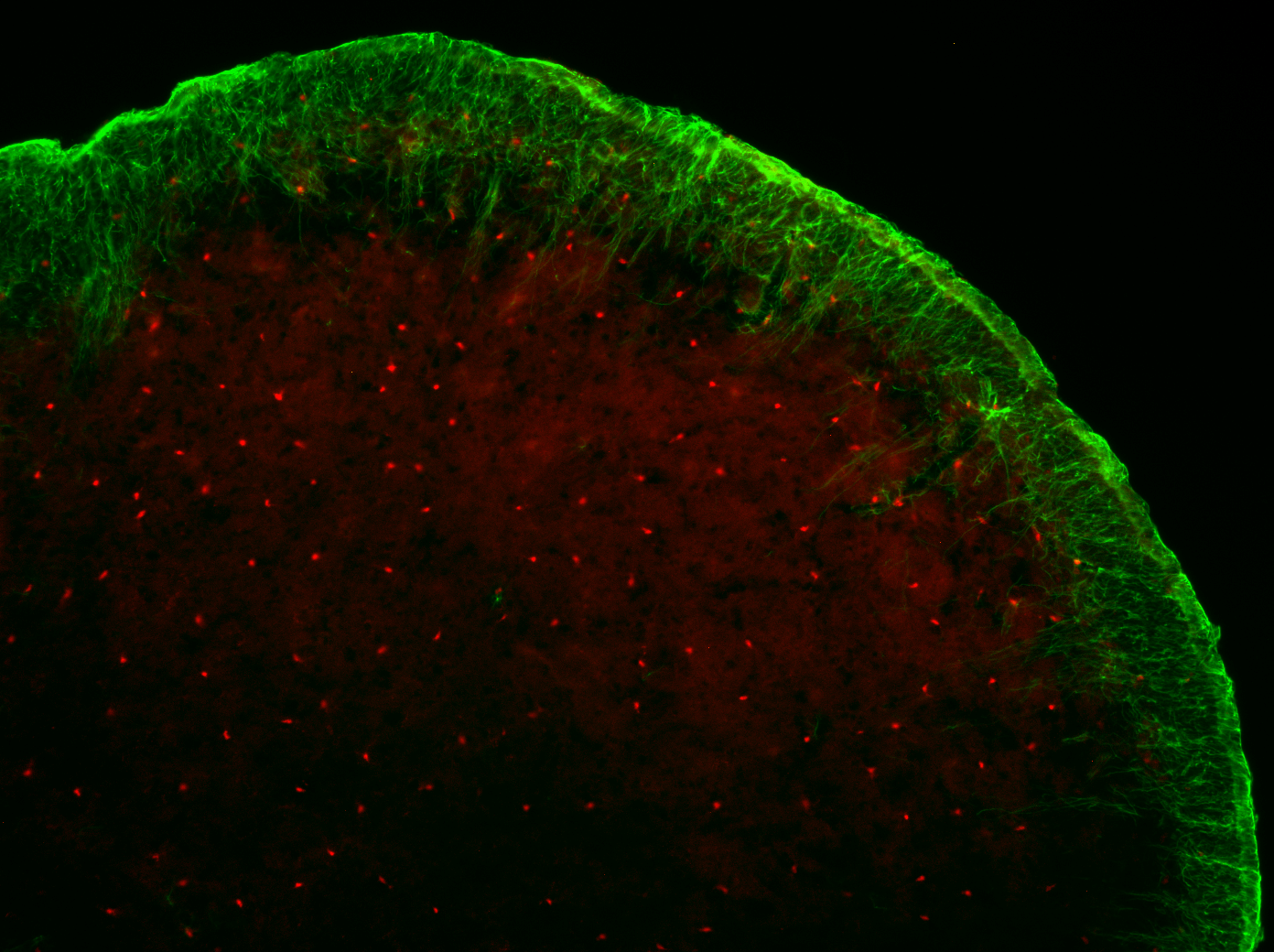

Supplement: Supplementary file 7 [file Data_Sheet_7_v1.ZIP › Figure 7/Figure 6C_overlay.tif]

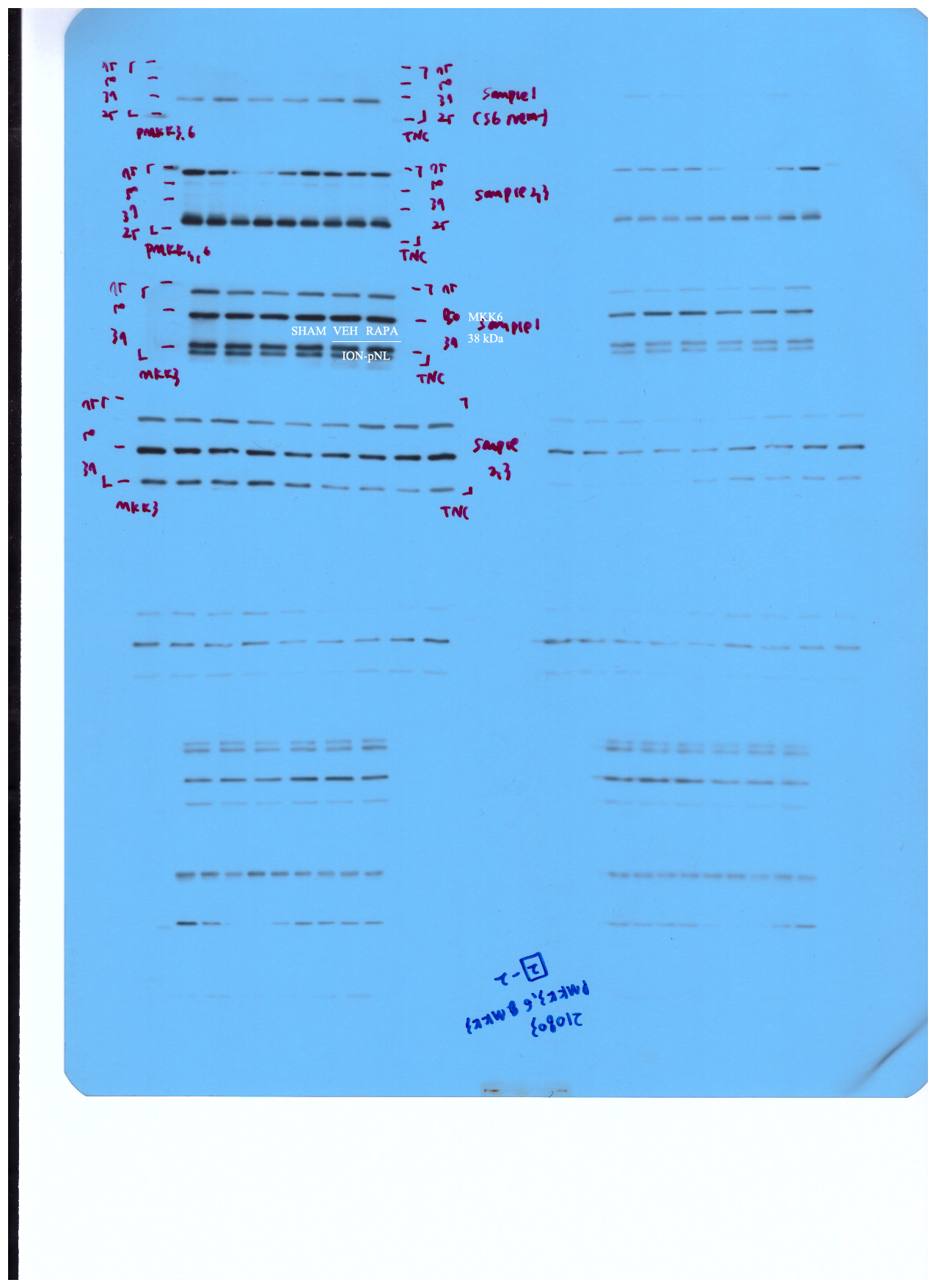

Supplement: Supplementary file 8 [file Data_Sheet_8_v1.ZIP › Figure 7/Figure 7A,B_MKK3.jpg]

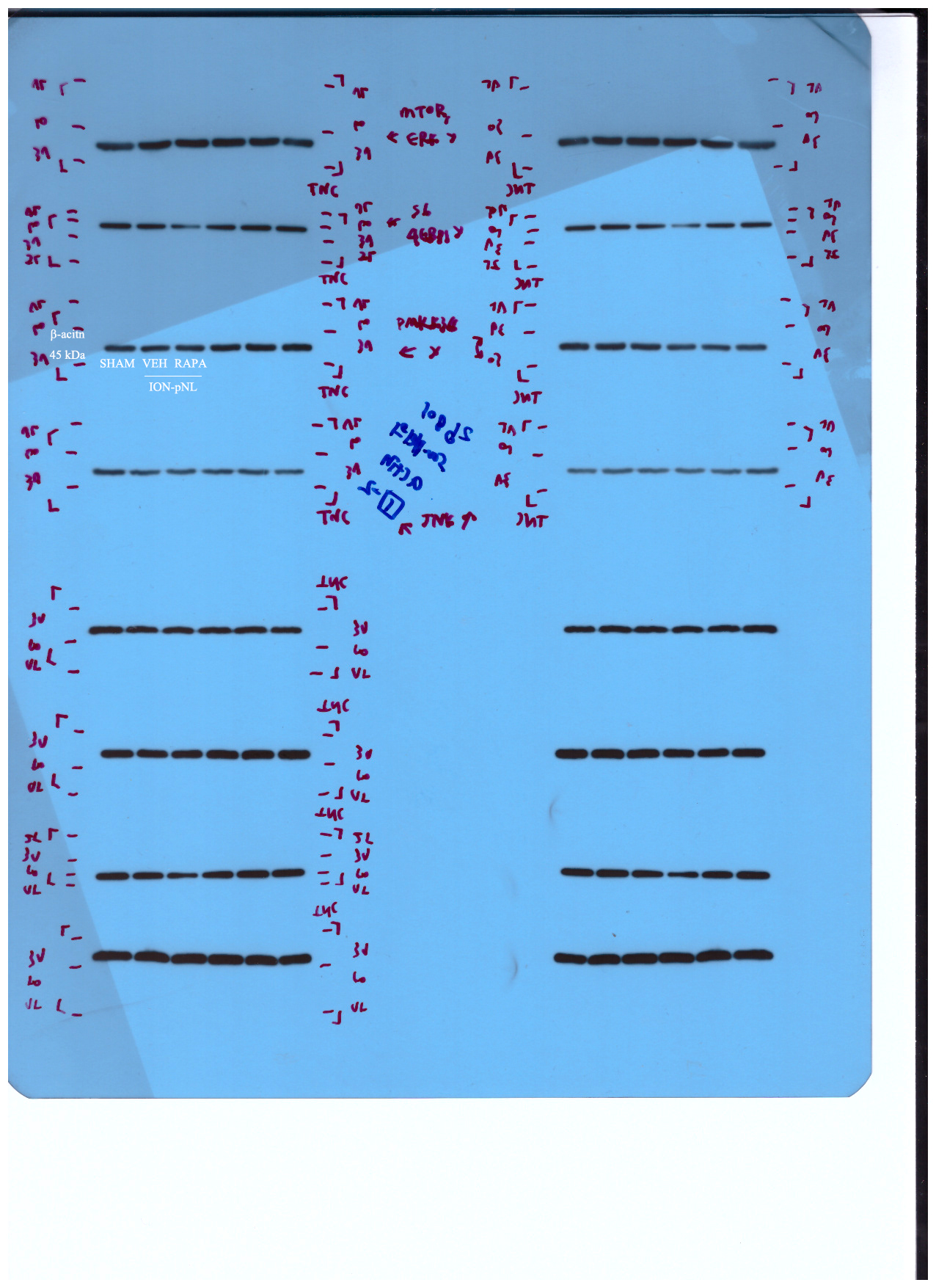

Supplement: Supplementary file 8 [file Data_Sheet_8_v1.ZIP › Figure 7/Figure 7A,B_actin of mkk3,6.jpg]

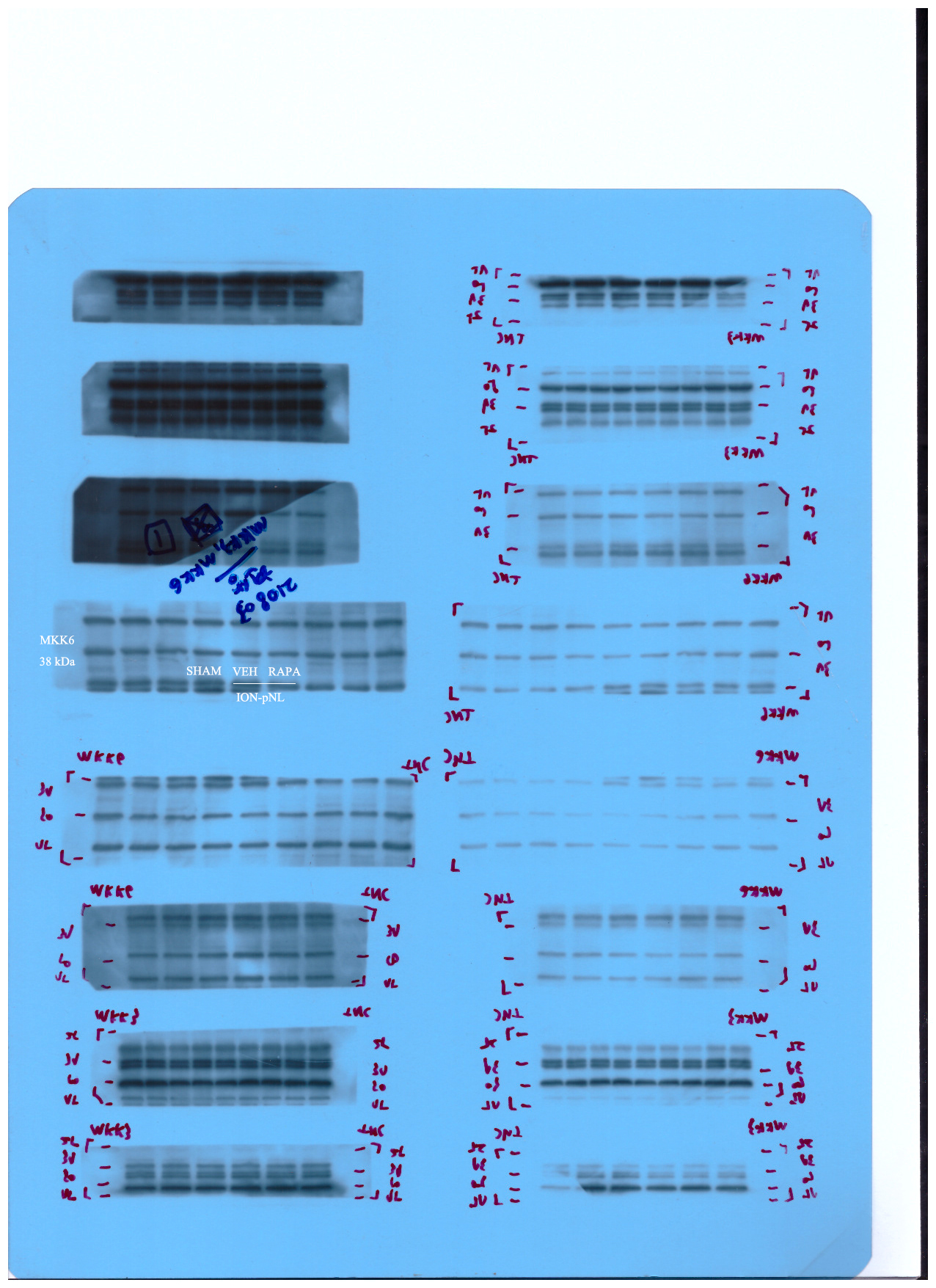

Supplement: Supplementary file 8 [file Data_Sheet_8_v1.ZIP › Figure 7/Figure 7A,B_MKK6.jpg]

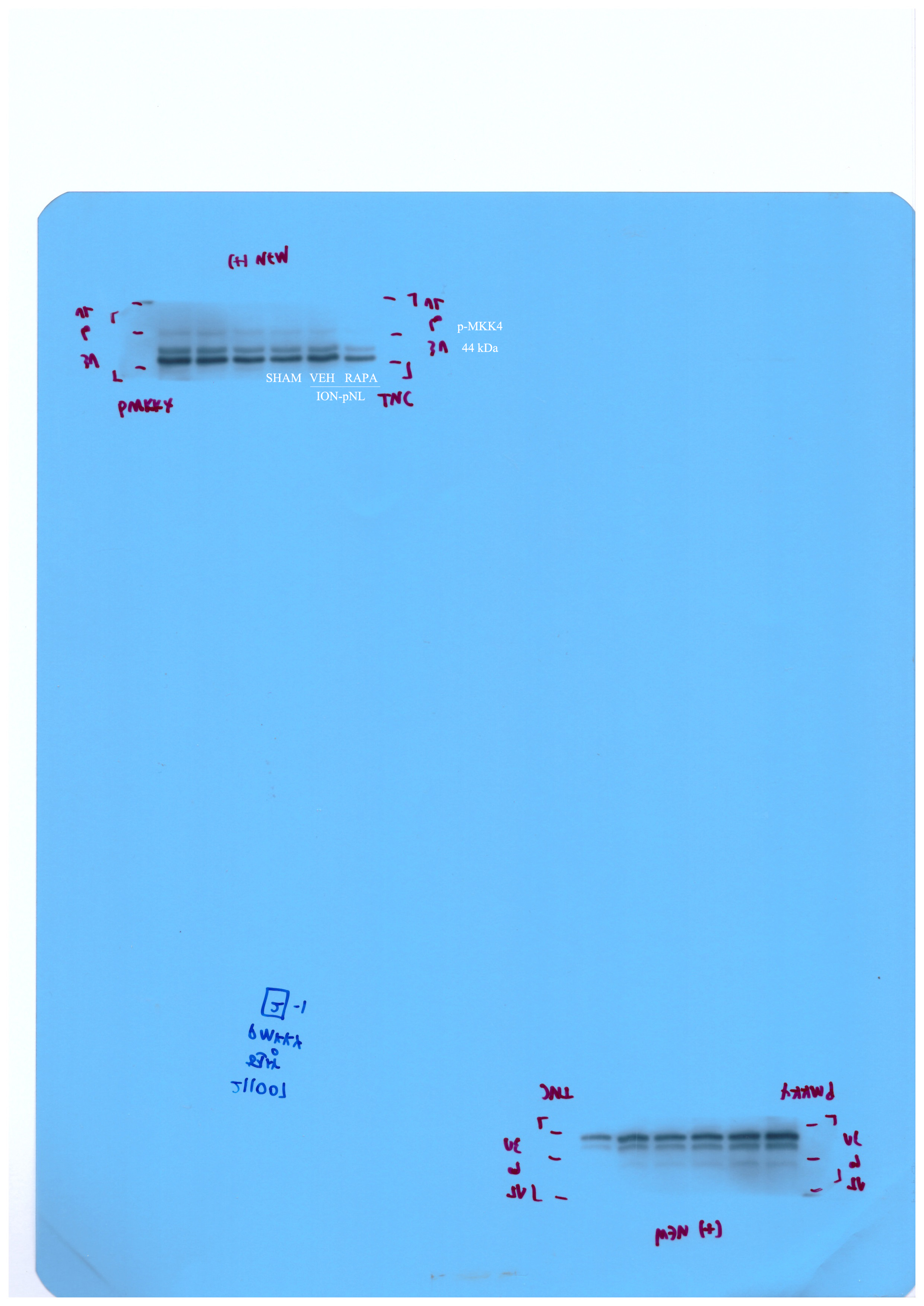

Supplement: Supplementary file 8 [file Data_Sheet_8_v1.ZIP › Figure 7/Figure 7A,C_p-MKK4.jpg]

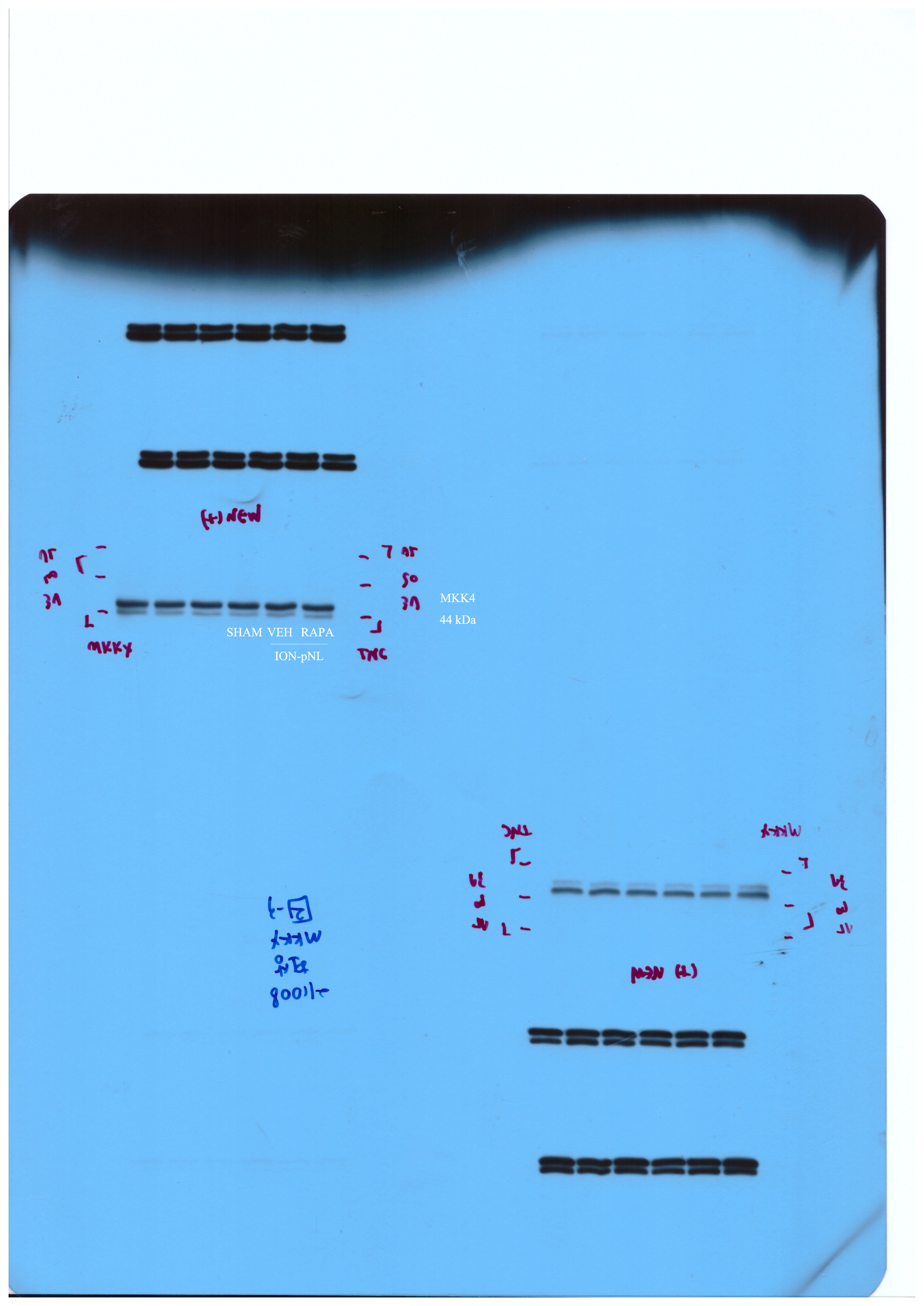

Supplement: Supplementary file 8 [file Data_Sheet_8_v1.ZIP › Figure 7/Figure 7A,C_MKK4.jpg]

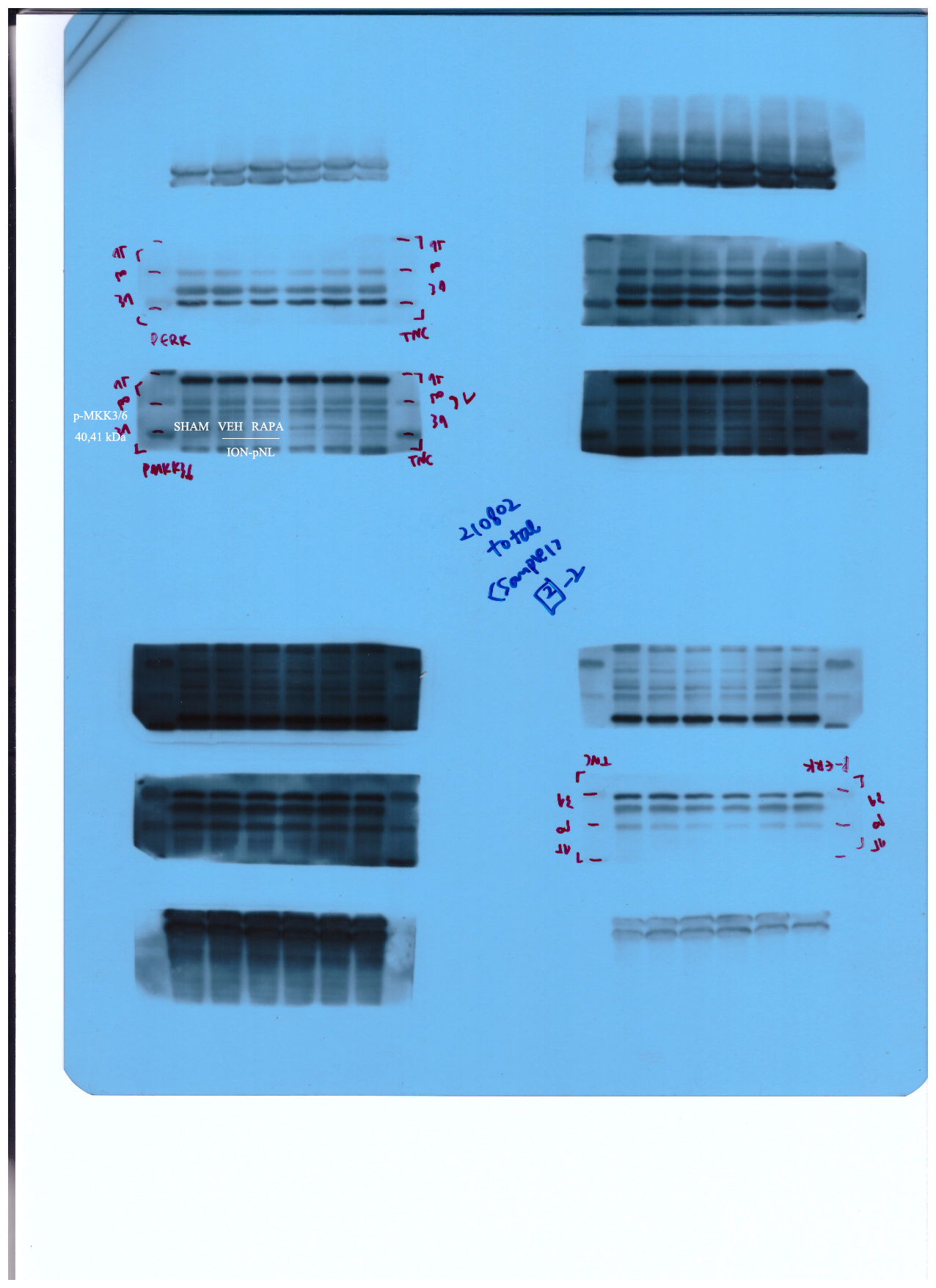

Supplement: Supplementary file 8 [file Data_Sheet_8_v1.ZIP › Figure 7/Figure 7A,B_p-MKK3,6.jpg]

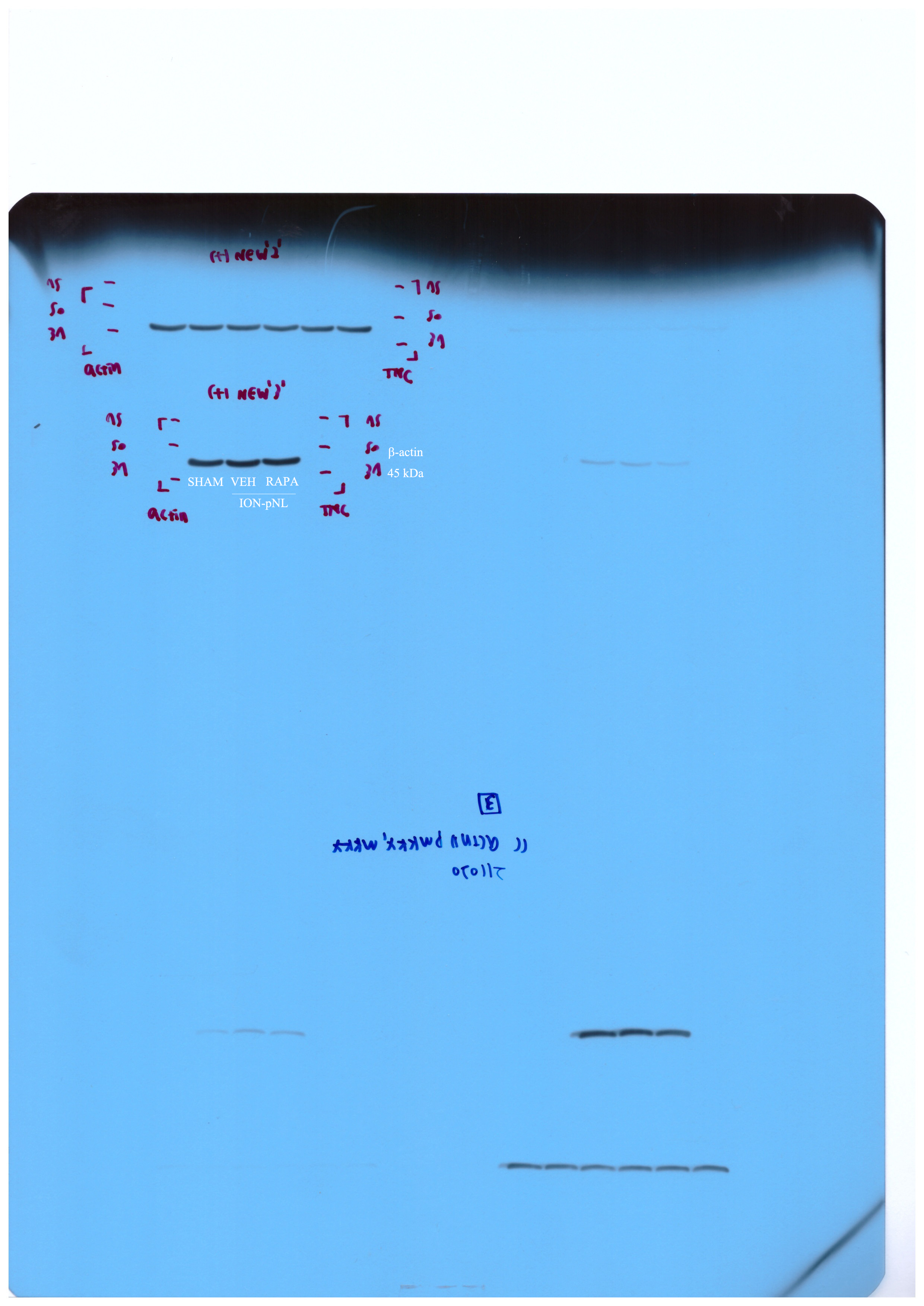

Supplement: Supplementary file 8 [file Data_Sheet_8_v1.ZIP › Figure 7/Figure 7A,C_actin of mkk4,p-mkk4.jpg]
